# DynamiSpectra: A Python Software Package and Web Platform for Molecular Dynamics Data Analysis in Computational Biology

**DOI:** 10.1021/acs.jcim.5c01270

**Published:** 2025-08-20

**Authors:** Iverson Conrado Bezerra, Jéssika de Oliveira Viana, Karen Cacilda Weber, Priscila Gubert

**Affiliations:** † Keizo Asami Institute, iLIKA, 28116 Federal University of Pernambuco, Recife, Pernambuco 50670-901, Brazil; ‡ Graduate Program in Biology Applied to Health, PPGBAS, Federal University of Pernambuco, Recife, Pernambuco 50670-901, Brazil; § Graduate Program in Pure and Applied Chemistry, POSQUIPA, Federal University of Western Bahia, Barreiras, Bahia 47808-021, Brazil; ∥ National Institute of Science and Technology on Molecular Sciences - INCT-CiMol, Department of Chemistry, Federal University of Paraíba, João Pessoa, Paraíba 58051-900, Brazil; ⊥ Chemistry Department, Federal University of Paraíba, João Pessoa, Paraíba 58051-900, Brazil

## Abstract

Molecular dynamics (MD) simulations generate extensive data sets that demand reliable and reproducible analysis tools. In this study, we present DynamiSpectra, a Python-based software package and web platform designed to automate the descriptive statistical analysis (mean and standard deviation) and visualization of MD trajectories. DynamiSpectra enables streamlined processing of GROMACS-generated files, supporting comparative analyses across multiple simulation replicas without requiring topology file handling or programming expertise. The package performs key structural and dynamic analyses, including RMSD, RMSF, radius of gyration, SASA, hydrogen bonds, salt bridges, secondary structure probability and fraction, principal component analysis, and ligand occupancy maps, producing high-quality graphical outputs with integrated descriptive statistical analysis. Additionally, it supports analyses such as protein–ligand contacts, protein–ligand minimum distance, protein–ligand hydrophobic contacts, inter-residue distance matrices, phi and psi angles, rotamers (χ1 and χ2), ligand dihedral angles, as well as analyses of pressure, temperature, and density within the system. Comparative tests performed against widely used MD analysis packages revealed that the results generated by DynamiSpectra were consistent with those tools. DynamiSpectra stands out for its ability to automate the analysis of multiple replicas, as well as the calculation of mean and standard deviation, which other packages often lack in automation. A use case involving β-amyloid peptide simulations at different temperatures illustrates the platform’s capabilities. Furthermore, the DynamiSpectra web interface enables users to upload data, generate interactive plots, and explore results without requiring local installation, thereby promoting accessible and reproducible MD analysis, being another essential distinguishing feature of the tool. The software is freely available via PyPI (https://pypi.org/project/DynamiSpectra/) and GitHub (https://github.com/Conradoou/DynamiSpectra/tree/main). The online web server can be accessed at: https://dynamispectra.onrender.com.

## Graphical abstract

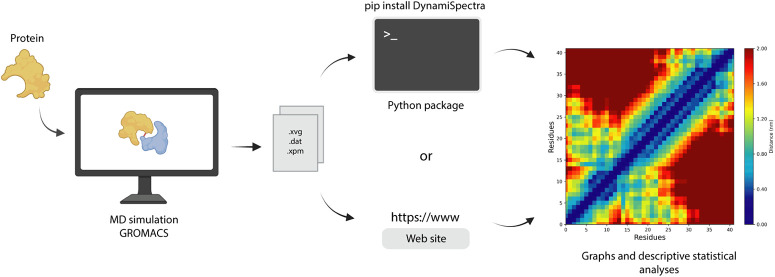

## 1 Introduction

Molecular dynamics (MD) is a computational technique used to predict the dynamic behavior of atomic motion in proteins or other molecular systems over time, based on physical models of interatomic interactions.^1^ The growing application of MD in the discovery of new drugs, investigation of the molecular behavior of proteins, determination of structural mechanisms of biomolecules, and nanomaterials is just a few of its numerous capabilities.^2,3^ However, a substantial expansion in the amount of data generated in simulations has sparked global interest in developing new algorithms and metrics for data analysis.^4^

The data generated by MD simulations are extensive, considering the number of molecular configurations stored along the trajectory and the number of simulated particles.^4^ Researchers can rely on several tools or packages for most of their analyses. The software AMBER,^5^ CHARMM,^6^ GROMACS,^7^ and NAMD,^8^ among others, are used to study the dynamics of molecular systems, generating vast amounts of data related to the simulations.^9^ However, implementing analysis algorithms can be difficult within existing software, requiring in-depth knowledge of its internal components.^10^

Experimental reproducibility is a central theme in science.^11^ In MD simulations, multiple replicas are highly recommended to increase the reproducibility and reliability of the results.^12^ Furthermore, studies suggest that multiple replicas are preferred over single or long simulations in terms of reproducibility and reliability.^12^ Therefore, there is a need to develop data analysis software that can integrate different replicas for the same system, providing the statistical insights necessary for researchers to draw conclusions and test hypotheses. Furthermore, it is essential to develop algorithms and tools for MD analysis that are accessible to users with technical limitations.

Offering user-friendly tools enables researchers without in-depth knowledge of computational science to perform complex analyses in a simple way. Therefore, with open-source code, we developed the DynamiSpectra package (https://conradoou.github.io/DynamiSpectra/), simplifying data analysis using the Python programming language (www.python.org). DynamiSpectra utilizes well-established Python libraries, including NumPy,^13^ SciPy,^14^ and Matplotlib,^15^ for numerical computations and data visualization. Additionally, we have developed a web tool that simplifies data analysis, offering enhanced features and an improved user experience. The DynamiSpectra web platform can be accessed at: https://dynamispectra.onrender.com. The package can be installed via PyPI using pip install DynamiSpectra, and its source code is publicly available on GitHub: https://github.com/Conradoou/DynamiSpectra and PyPI: https://pypi.org/project/DynamiSpectra/.

DynamiSpectra differs from existing analysis packages due to its descriptive statistical capability of handling replicas, enabling the analysis of means and standard deviations, and encouraging researchers to apply replicas in their research. Furthermore, compared to other available packages, DynamiSpectra does not require the processing of topology files, as it is performed directly within the simulation source software and then integrated into the package. Another essential feature is the ease of function calls with standardized directory paths between the different analyses, which facilitates and speeds up data analysis. DynamiSpectra does not require users to have programming skills. However, analyses in DynamiSpectra are currently limited to processing output files from GROMACS with the .xvg, .dat, and .xpm extensions, since it does not accept raw trajectory or topology input.

Herein, we present the basic structure and functionalities of DynamiSpectra. We simulated the β-amyloid peptide (Aβ) bound to a quinoline derivative to demonstrate the package’s functionality at various temperatures. DynamiSpectra supports descriptive statistical summarization (mean and standard deviation) across multiple independent simulation replicas, in addition to analyzing Root Mean Square Deviation (RMSD), Root Mean Square Fluctuation (RMSF), Radius of Gyration (Rg), Solvent Accessible Surface Area (SASA), Hydrogen Bonds (Hbond), Kernel Density Estimation (KDE), Salt Bridges, Principal Component Analysis (PCA), Secondary Structure Probability, Secondary Structure Fraction, and Ligand Occupancy Maps. Furthermore, it includes analyses of protein–ligand contacts, protein–ligand minimum distance, protein–ligand hydrophobic contacts, inter-residue distance matrices, phi and psi angles, rotamers (χ1 and χ2), ligand dihedral angles, as well as system properties such as pressure, temperature, and density.

## 2 Simulations Details

To demonstrate the applications of DynamiSpectra, we focused on studying the β-amyloid peptide (Aβ). The Aβ peptide plays a central role in the pathogenesis of Alzheimer’s disease, being involved in the formation of toxic aggregates, senile plaques, and damage to brain tissue.^16^ The structural dynamics, flexibility, and intermolecular interactions determine its aggregation potential.^17,18^ The Aβ peptide may be a potential target for developing new therapies.^19^

The Aβ peptide bound to a quinoline derivative was simulated using the GROMOS 54A7 force field using the GROMACS package 2023.5,^20,21^ with a dodecahedral box with dimensions of 7.28 × 7.28 × 5.14 nm. Water was used as a solvent with the SPC model.^22^ The system’s charges were neutralized by adding Na^+^.^23^ The system was equilibrated under constant pressure and temperature conditions for 100 ps, and the simulation was performed for 50 ns with an integration time of 2 fs. Simulation temperatures were performed at 300 K (simulation 1), 310 K (simulation 2), and 318 K (simulation 3), with temperature controlled by the V-rescale thermostat and pressure maintained at 1 bar using the Parrinello–Rahman barostat under isotropic conditions.

## 3 Overview of the Examples

In this session, we present 11 examples that illustrate the functionalities in the DynamiSpectra package. Our examples utilize GROMACS simulation data in the .xvg, .dat, and .xpm file extensions, along with the modularized codes included in the package, to analyze and extract the data necessary for visualization and calculations. Python 3.12 version is used to apply the examples, and all the required corresponding files for user reproduction can be accessed in the DynamiSpectra repository on GitHub: https://github.com/Conradoou/DynamiSpectra/tree/main/data. Although the DynamiSpectra package tests were performed with the Aβ peptide, it also supports the analysis of proteins, isolated ligands, and complexes, since the structure and extension of the files are the same as those available in the repository. The user can define the number of replicas through input arguments. Furthermore, the analysis can be generated with a single replica. The tutorial on using the DynamiSpectra Python package (Video S1) and web platform (Video S2) is available in the Supporting Information and in the documentation.

### 3.1 Example 1: Time-Dependent Profile Analyses

We developed a unified line plot framework to visualize time-dependent properties, including RMSD (Figure S1A), Rg (Figure S1B), salt bridges (Figure S1C), number of hydrogen bonds (Figure S1D), and SASA (Figure 1A). For properties like RMSF (Figure S1E), which represent per-residue fluctuations rather than temporal changes, the plots show the value as a function of residue position. Although each property is displayed in a separate graph, all plots share the same graphical structure for consistency and ease of comparison. The *x*-axis represents the simulation time (ns), while the *y*-axis displays the arithmetic mean of the property of interest across the replicas, with the corresponding standard deviation indicated (Figure 1A). For these analyses, DynamiSpectra automatically computes the mean and standard deviation across replicas and simulations; however, users may also choose to analyze a single replica without averaging. Importantly, DynamiSpectra imposes no restrictions on the number of replicas or simulations, allowing users to include as many as needed. Each line in the plots represents the temporal evolution of the selected metric from a single simulation, with optional customization of graph style available directly in the code cell.

**Figure 1:**
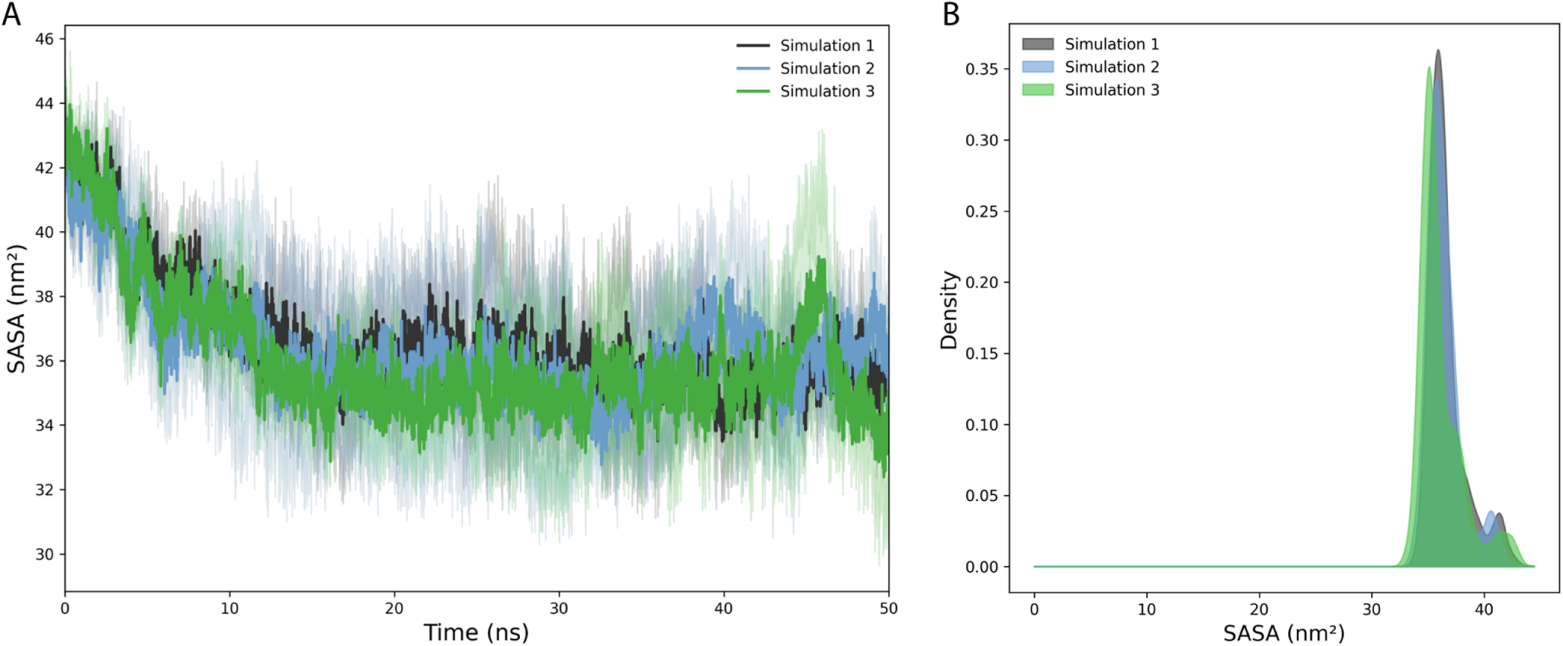
Time-dependent plot showing the peptide SASA throughout the MD simulation in panels. (A) SASA over time, and (B) Kernel Density Estimation plot of SASA. The similar graphical structure is applied to RMSD, RMSF, Hbonds, and salt bridge analysis.

Users can easily customize the plots without modifying the source code by using configuration dictionaries, such as rmsd_config, for example. This dictionary allows adjustments to simulation labels, plot colors, transparency of the standard deviation shading, figure dimensions, axis labels (xlabel and ylabel), and font sizes. The same customization options apply to the kernel density plot through the density_config dictionary.

### 3.2 Example 2: Kernel Density Estimation

Using Gaussian Kernel Density Estimation (KDE), we implemented a standardized method for generating density plots for all analysis types. As shown in Figure 1B, these plots visualize the probability distribution of each metric’s values across simulations. In this approach, each value from the simulation contributes to the overall distribution through a Gaussian kernel. The resulting probability density function *f̂*(*x*) is computed as in eq 1, where *n* is the number of observations, *x*_*i*_ are the observed values (e.g., SASA), and *h* is the bandwidth parameter controlling the smoothness of the curve.

1 $$\mathrm{f̂}\left(x\right)=\frac{1}{nh\sqrt{2\Pi }}\overset{n}{\underset{i=1}{\sum }}\exp\left(-\frac{{\left(x-x_{i}\right)}^{2}}{2h^{2}}\right)$$

The *x*-axis corresponds to the metric values range, while the *y*-axis shows the estimated probability density. As highlighted previously in Section 3.1, the kernel density plots can be customized directly within the code cell using the density_config dictionary.

### 3.3 Example 3: Secondary Structure Analysis: Probability and Fraction Over Time

We implemented two complementary visualization approaches to analyze secondary structure assignments along MD trajectories. In the first approach, a **boxplot-based visualization compares the distribution of secondary structure probabilities** across simulations (Figure 2A). For each frame *i*, the probability *P*_*i,s*_ of observing a specific secondary structure type *s* (e.g., α-helix, β-sheet) is calculated as ([Disp-formula eq2]):

2 $$P_{i,s}=\left(N_{i,s}\right)/\left(N_{\mathrm{total}}\right)\times 100$$

where *N*_*i,s*_ is the number of residues adopting structure *s* in frame *i*, and *N*_total_ is the total number of residues in the system. This yields the percentage of residues adopting each structural element per frame across the trajectory. The resulting box plots display the distribution of these probabilities with the *y*-axis representing the occurrence probability (%) and the *x*-axis categorizing structural components (α-helix, β-sheet, coil/loop, turn, bend, and 3-helix). Distinct colors are assigned to differentiate simulation groups, and the box plots show the distributions’ medians and quartiles.

**Figure 2:**
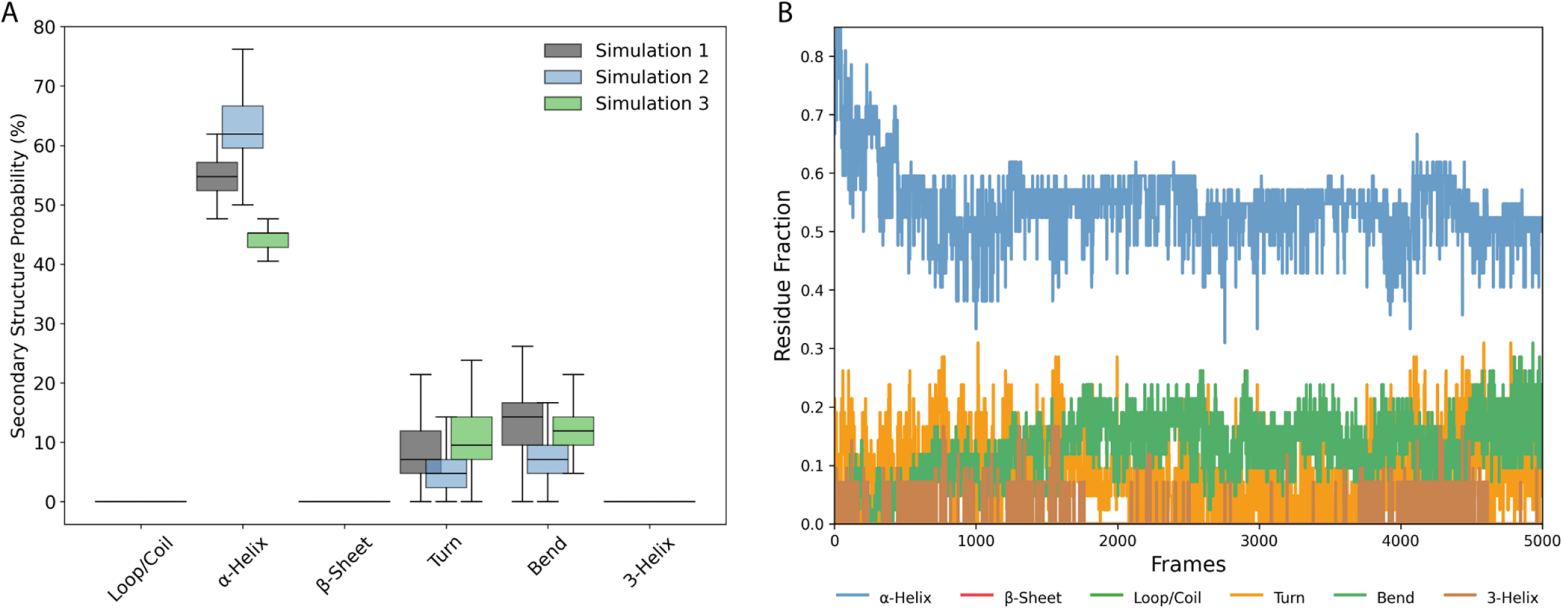
Secondary structure analysis of the peptide throughout the MD simulations. (A) Probability of each secondary structure element over time; (B) fractions of secondary structure elements averaged across the simulation time.

The second approach provides a **line plot representation of secondary structure fractions over time** (Figure 2B), focusing on the temporal evolution of these structural elements throughout the simulation. For each frame *i*, the fraction *F*_*i,s*_ of residues adopting a given secondary structure is calculated by ([Disp-formula eq3]):

3 $$F_{i,s}=\frac{N_{i,s}}{N_{\mathrm{total}}}$$

This metric tracks the proportion of the protein adopting each secondary structure type over time. Different colors highlight the structural components, as in the probability analysis. Both visualizations are highly flexible and customizable via the plot_config dictionary within the code cell, without requiring modification to the source code. This configuration allows users to define simulation group labels, select specific colors, adjust axis labels, control figure size and axis limits, and tune transparency. This flexibility ensures that the figures can be easily adapted for different presentation or publication requirements. In addition to graphical outputs, DynamiSpectra automatically generates a spreadsheet (.xlsx) containing the calculated secondary structure fractions for each frame and for each structural class, providing users with a detailed and accessible data set for further quantitative analysis.

### 3.4 Example 4: Principal Component Analysis

We developed a quantitative dot plot to analyze conformational dynamics via dimensionality reduction (Figure 3A). The *x*-axis represents the first principal component (PC1), while the *y*-axis corresponds to the second principal component (PC2). Each point in the plot is color-coded to reflect the progression of the simulation, illustrating its temporal evolution from start to finish. The pca_analysis function enables extensive user customization within the code cell, allowing modification of the plot title, figure size, colormap, marker size, as well as font sizes for axis labels and tick labels. This flexibility ensures that the scatter plot can be tailored to specific visualization preferences and presentation requirements.

**Figure 3:**
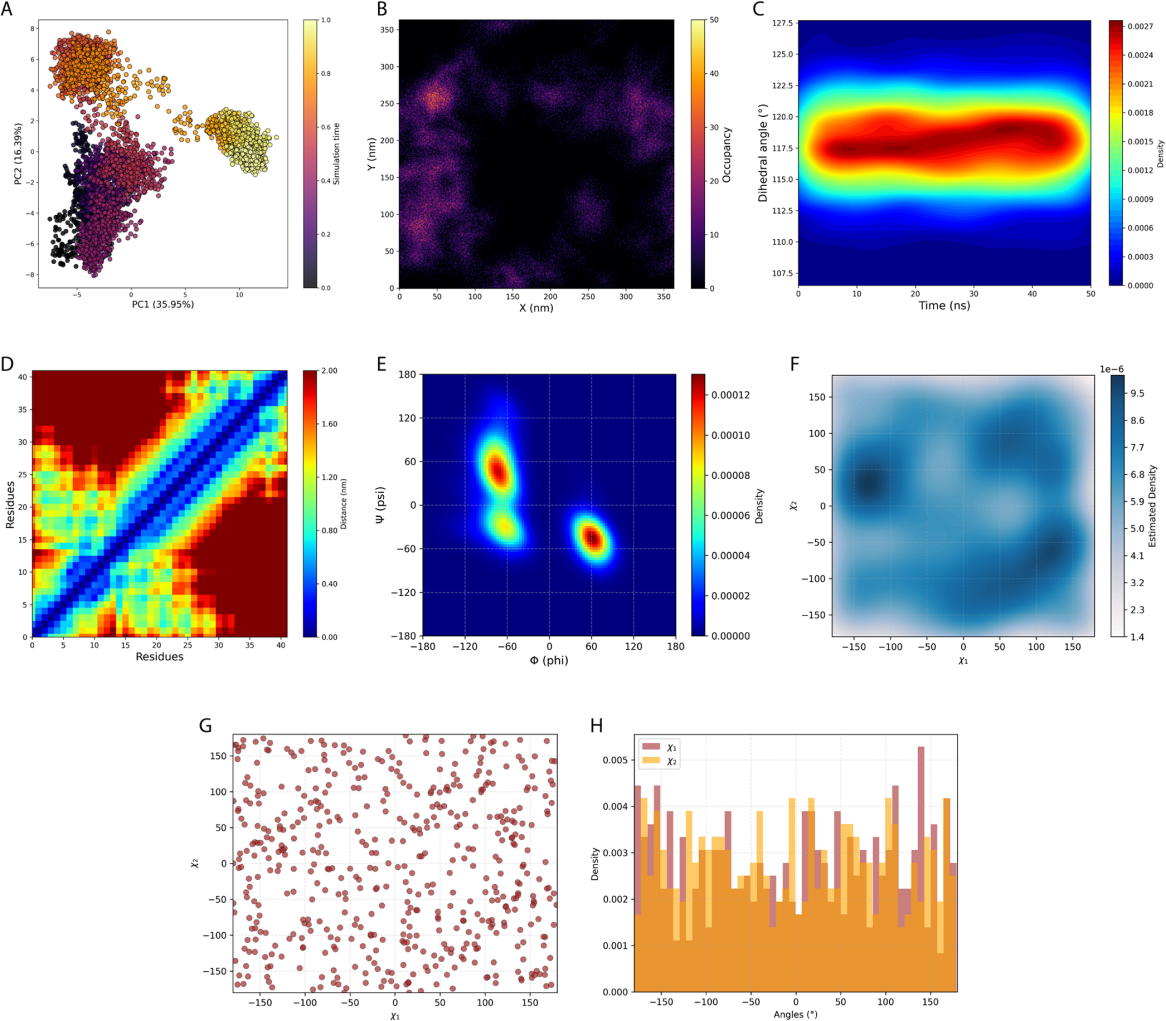
Structural and conformational analysis of the peptide–ligand system from the MD simulations. (A) Principal component analysis of the peptide atomic fluctuations; (B) density map of the ligand position throughout the simulation; (C) 2D KDE plot of the ligand angle distribution; (D) inter-residue distance matrix; (E) Ramachandran plot showing φ and ψ backbone angles; (F) 2D KDE plot of the average χ1 and χ2 Leu34 rotamers; (G) dot plot of the average χ1 and χ2 Leu34 rotamers; and (H) histogram of the average χ1 and χ2 Leu34 rotamers.

### 3.5 Example 5: Protein–Ligand Contacts and Minimum Distances

We implemented a comprehensive set of analyses to investigate protein–ligand interactions, focusing on three key metrics: protein–ligand contacts, protein–ligand hydrophobic contacts, and protein–ligand minimum distances. These analyses enable the evaluation of how the ligand interacts with the protein throughout MD simulations, providing insights into both the frequency and nature of these interactions.^24^

The protein–ligand contacts analysis (Figure S2A) quantifies the number of atomic contacts between the ligand and the protein along the simulation trajectory. The contact_config dictionary allows users to customize the visualization by modifying the labels for the simulation groups, the line colors, the transparency of the shaded area representing the standard deviation, as well as the axis labels and font sizes. The protein–ligand hydrophobic contacts analysis (Figure S2B) focuses specifically on the hydrophobic interactions between the ligand and the protein. For this analysis, the user must define which hydrophobic residues within the protein are likely to interact with the ligand during the simulation. The analysis then selects and quantifies only the contacts formed between the ligand and these specified hydrophobic residues, providing a detailed understanding of the contribution of hydrophobic contacts to ligand binding. The contact_config dictionary provides full control over visualization settings.

The protein–ligand minimum distances analysis (Figure S2C) monitors the shortest distance between any atom of the ligand and any atom of the protein throughout the trajectory, providing insights into ligand stability, binding persistence, and potential dissociation events.^25^ Visualization customization is handled through the distance_config dictionary, offering the same flexibility as contact_config for adjusting labels, colors, transparency, and axis formatting.

These analyses are designed to accommodate any number of simulations and replicas, providing a flexible and customizable approach to explore protein–ligand interactions through clear and informative graphical representations. By default, the analysis reports the arithmetic mean and standard deviation across replicas; however, users may also choose to analyze a single simulation if desired. There are no limitations on the number of replicas or simulation groups that can be analyzed.

### 3.6 Example 6: Ligand Occupancy Map

We implemented a quantitative heatmap to analyze the relative density distribution of ligands (Figure 3B). Ligand atomic positions are projected onto the *XY* plane by averaging coordinates along the *Z*-axis across frames. The *X* and *Y* axes represent the physical spatial coordinates of the simulation box (nm), corresponding precisely to the box dimensions along the *X* and *Y* directions, respectively, consistent with the Cartesian coordinate system used in MD.^26^ These labels reflect actual spatial positions, providing a meaningful physical context to the density map. The color scale indicates relative ligand density, with warmer colors highlighting regions of higher occupancy.

The ligand_density_analysis function allows users to customize the colormap, axis labels, title, figure size, and font sizes directly within the code cell, enabling flexible and user-friendly visualization without modifying source code.

### 3.7 Example 7: Ligand Dihedral Angle Monitoring

This analysis generates three distinct plots to characterize ligand dihedral angle dynamics during simulations. The analyses can be performed using multiple replicas and simulations, but it is also possible to analyze a single simulation. The line plot displays the circular mean dihedral angle over time (Figure S3A). The 2D KDE heatmaps illustrate the temporal distribution of dihedral angles, showing the circular mean for the simulations (Figure 3C). The use of the circular mean is essential to accurately average angular data due to its periodic nature (0° is equivalent to 360°).^27,28^ Additionally, the density distribution plot depicts the overall distribution of dihedral angles across the trajectory, with density on the *y*-axis and angle values on the *x*-axis, highlighting conformational preferences regardless of time (Figure S3B).

Users are required to define the relevant dihedral atoms in a GROMACS index (.ndx) file to perform this analysis and generate the .xvg file. Customization of the plots can be performed directly in the code cell through the dictionaries time_config, density_config, and kde2d_config without requiring changes in the source code.

### 3.8 Example 8: Inter-Residue Distance Matrix

The inter-residue distance matrix provides a detailed visualization of pairwise distances between residues within the protein (Figure 3D). Both the *x*- and *y*-axes correspond to the residue indices along the protein sequence, and each cell within the matrix represents the spatial distance between a specific pair of residues. The resulting matrix is displayed as a 2D heatmap, where the color intensity encodes the magnitude of the distances: lighter colors indicate residues in closer spatial proximity, whereas darker regions correspond to residue pairs separated by greater distances. This visualization enables the identification of compact structural regions, stable contacts, flexible segments, and potential conformational rearrangements during the simulation.

The analysis operates on .xpm files generated by GROMACS, which contain the pairwise distance data in matrix form. Users can adjust plot attributes directly within the analysis code cell through the config dictionary, allowing customization of *x* and *y*-axis labels, font sizes, figure size, plot title, and the colormap for optimal interpretability and presentation.

### 3.9 Example 9: Phi and Psi Angles

We implemented a detailed analytical framework to assess backbone dihedral angles φ (phi) and ψ (psi) from MD simulations. This analysis captures the conformational preferences and flexibility of protein backbones by evaluating the distribution of these angles over time, using data extracted from .xvg files. The results enable the identification of predominant conformational states, the detection of transitions between structural regions, and the visualization of rare or transient conformations.

For simulations with multiple replicas, DynamiSpectra generates individual 2D KDE plots for each replica and a combined plot by concatenating all replicas within the same simulation group. Concatenation, rather than averaging, ensures a more robust and statistically representative depiction of the whole conformational space sampled,^29^ offering a clearer overview of φ and ψ angle distributions and enhancing the analysis of protein flexibility and structural preferences.

The resulting plots display φ on the *x*-axis and ψ on the *y*-axis, with color intensity representing the density of occurrences (Figure 3E). DynamiSpectra automatically generates and saves individual plots for each replica and a combined plot concatenating all replicas from the same simulation group, offering flexibility for detailed per-replica evaluation or global assessment of conformational space. The user can fully customize these visual outputs through the phipsi_config dictionary within the code cell.

### 3.10 Example 10: Rotamers (χ1 and χ2) Angles

DynamiSpectra provides a comprehensive framework for analyzing protein side chain rotameric states by examining the χ1 and χ2 dihedral angles during MD simulations. The analysis uses input .xvg files and allows the user to include either a single replica or multiple replicas per simulation. When multiple replicas are provided, DynamiSpectra calculates the circular mean of the angles to give a meaningful representation that accounts for the periodic nature of dihedral angles.

The module generates different plots to characterize rotamer behavior: 2D KDE heatmaps that highlight regions of high density in χ1/χ2 space for detailed identification of dominant rotameric states and transitions (Figure 3F), dot plots that depict χ1 versus χ2 angles for each frame as scatterplots to reveal preferred rotameric clusters and conformational variability (Figure 3G), and histograms showing the frequency distribution of χ1 and χ2 angles sampled at each frame (Figure 3H).

Users can specify a particular simulation time window for analysis by adjusting the time_window function in the code cell, allowing for a focused investigation of specific intervals. All graphical outputs are highly customizable through the function dihedral_kde_and_dotplot, which enables modifications to the colormap, figure size, font sizes, axis labels, and other visual elements.

### 3.11 Example 11: Thermodynamic Properties Monitoring

DynamiSpectra offers dedicated modules for analyzing and visualizing temperature (Figure 4), pressure (Figure S4A), and density (Figure S4B) profiles throughout MD simulations. These analyses help evaluate the system’s equilibration, stability, and overall behavior of thermostats and barostats over time. The resulting plots display each property as a function of simulation time, providing a clear overview of system stability across replicas and simulations. For each property, DynamiSpectra also generates KDE plots, which complement the time-dependent plots by depicting the distribution of observed values, facilitating the identification of fluctuations, stability, or rare events.

**Figure 4:**
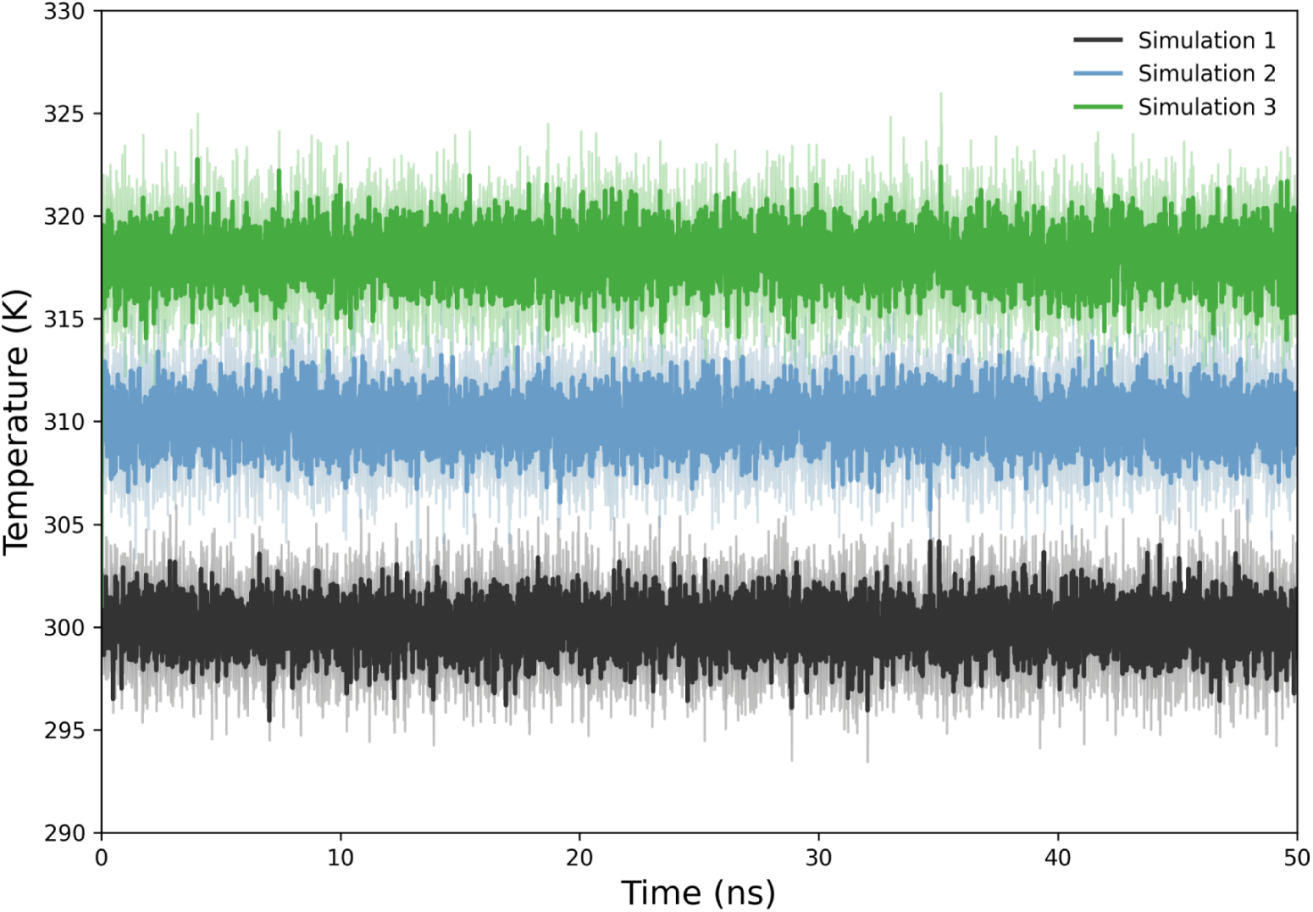
Temperature profile of the system throughout the MD simulation, showing the stability of the temperature over time.

Visualization settings can be flexibly adjusted through dedicated configuration dictionaries within the analysis code cells (e.g., pressure_config, temp_config, and density_config). These analyses accept .xvg files, which GROMACS typically generates during simulations or through its energy analysis tools. Plots are generated using the arithmetic mean and standard deviation across multiple replicas and simulations; however, users may also choose to analyze a single replica from a single simulation.

Since DynamiSpectra was developed with a strong focus on supporting GROMACS outputs, specifically files with .xvg, .dat, and .xpm extensions that follow GROMACS conventions, it currently does not guarantee compatibility with output files from other MD softwares. This limitation arises from the fact that the current file parsers are specifically designed for the structure, headers, and formatting conventions of GROMACS-generated data.

Nonetheless, we acknowledge that other analysis tools, such as CPPTRAJ (from the AMBER suite), can produce output files (e.g., .dat) that are potentially compatible with DynamiSpectra. Although these file types are not officially supported at this stage for some types of analysis, preliminary tests indicate that the software can successfully process them, provided they adhere to a format consistent with the expected time-value structure.

To enhance interoperability and broaden usability, we plan to develop in the future a flexible data processing layer capable of handling generic time-series outputs generated by various MD analysis tools, including MDAnalysis^10^ and MDTraj.^30^ This addition will enable DynamiSpectra to recognize automatically and parse tabular data, provided the file structure is consistent with standard formats. These improvements are part of our planned future updates and are expected to substantially expand DynamiSpectra’s applicability across a wide range of simulation platforms.

## 4 Cross-Verification and Advantages with Established Tools

The DynamiSpectra package provides an automated and simplified approach to visualizing and analyzing MD outputs, distinguishing it from traditional tools such as MDplot and GRACE (xmgrace).

MDplot^31^ is a reliable and widely used package for the visualization of MD outputs. It offers built-in support for handling multiple simulations, allowing for the calculation and visualization of means and standard deviations. For this reason, MDplot was selected as a reference tool to validate the outputs of DynamiSpectra. An important distinction, however, is that MDplot operates within the R environment (https://www.r-project.org/), while DynamiSpectra is fully implemented in Python, providing flexibility for users depending on their preferred programming ecosystem. Similarly, xmgrace (https://plasma-gate.weizmann.ac.il/Grace/) is a robust tool for generating scientific plots, commonly used in molecular dynamics for visualizing time-dependent properties. However, xmgrace provides only basic data manipulation functions, not performing advanced data analysis or processing.

DynamiSpectra differs by fully automating these steps. Operating directly on GROMACS .xvg outputs, it allows for the simultaneous processing of multiple replicas, automatically calculating descriptive statistical parameters, and generating consolidated plots easily customizable. As a validation example, analyses were cross-verified among DynamiSpectra, MDplot, and xmgrace (Figure S5). The results were identical in all tools, confirming DynamiSpectra’s reliability and its consistency with established methodologies.

In addition, by working directly with GROMACS-native .xvg, .xpm, and .dat files, DynamiSpectra minimizes methodological divergence and potential user error arising from reformatting or reprocessing data externally. This ensures consistency, reduces user workload, and delivers publication-ready outputs through a fully integrated workflow. Furthermore, DynamiSpectra is highly user-friendly, as its documentation provides all the necessary code cell commands explicitly, allowing users to simply adjust directory paths to their data sets and easily customize the graphical interface according to their preferences.

Several tools have been developed for analyzing molecular dynamics simulations, including mdciao,^32^ CHAPERONg,^33^ MD-TASK,^34^ and QwikMD.^35^ These applications are primarily focused on processing trajectory and topology files (e.g., .xtc, .trr, .pdb, .gro) and analyzing atomic interactions, structural fluctuations, and related properties. In contrast, DynamiSpectra was explicitly designed to operate on postprocessed outputs from GROMACS, streamlining the analysis of multiple replicates through automated mean and standard deviation treatment and generation of high-quality plots, without the need for trajectory files. This differentiates DynamiSpectra by offering a more accessible, focused, and automated solution for users working directly with GROMACS output files.

## 5 Web Interface for Accessible Analysis

Despite the growing advancement of computational biology, many data analysis tools require prior and robust knowledge in programming or interaction with command lines, a barrier for many researcher.^36^ Therefore, DynamiSpectra has a graphical interface building with the Flask framework that facilitates researchers in the automated analysis of data in an easy way. User-friendly tools are essential in computational biology, enabling analyses to be performed even without prior experience in various programming languages. It is available at the following link: https://dynamispectra.onrender.com.

The DynamiSpectra Web site, in the initial section, offers a clear discussion of the tool’s potential. In the analysis session, the user can upload files in the .xvg extension, and generate the general graph in interactive mode in the Analyses page with the possibility of analyzing independent simulations, and the mean and standard deviation for replicas. The graphs allow manipulation of the axes, zoom and navigation, graph displacement, download, selection of specific intervals, among others. In addition, the graphs enable the display of numerical values corresponding to the x and y axes of the plot lines through tooltips, facilitating and accelerating the user’s analysis.

This approach promotes the democratization of MD analysis, enabling research groups worldwide, even those without computational support, to explore and visualize complex data in an accessible and reproducible manner. The web system was designed for future expansion and improvement of the tool, enabling the inclusion of new analyses and using the same codes as the DynamiSpectra package. The web platform can also be helpful for scientific and educational research purposes. In addition, the results obtained via the web platform are consistent with those obtained with the application of the Python package, offering reliability. As a web application, the DynamiSpectra web tool is compatible with multiple platforms (Windows, macOS, Linux) and can be accessed directly through the browser, without needing local installation.

Furthermore, to assist users in interpreting the results, the DynamiSpectra web platform provides an integrated floating panel for each analysis type. These floating panels deliver concise, literature-supported guidelines on how to interpret the trends observed in these plots. Full details are provided in Table S1.

## 6 Conclusions

DynamiSpectra is a software package for descriptive statistical analysis and graph visualizations from MD simulation data. Using test simulations of Aβ peptide bound to quinoline derivatives at different temperatures, we demonstrate the generation of several graphs that cover relevant structural and dynamic aspects.

In analyzing structural elements, we highlight the evaluation of structural deviations, local flexibility, structural compaction, solvent exposure, hydrogen bond formation, salt bridge, secondary structure content, ligand spatial density, protein–ligand contacts, hydrophobic contacts, minimum distances, inter-residue distance matrices, backbone dihedral angles (φ and ψ), rotameric states (χ1 and χ2), ligand dihedral angles, as well as system-level properties such as pressure, temperature, and density. The package allows both isolated and comparative analyses across any number of simulation replicas or independent simulations, facilitating and encouraging the use of multiple replicas in MD studies to ensure robustness and reproducibility of results.

DynamiSpectra has a web graphical interface under development using the modularized scripting system of the Python package. The web tool enables the generation and descriptive statistical analysis of data, producing graphs that can be manipulated using tools and tooltips, while also ensuring ease of use for users with no prior programming experience. Thus, DynamiSpectra offers a comprehensive and customizable platform for visually exploring and interpreting molecular simulation data, focusing on promoting reproducible and informative analyses, and facilitating and democratizing MD analysis.

## Supplementary Material









## Data Availability

All tools and data used in this study are available in the open-source DynamiSpectra software package. The source code is hosted on GitHub at https://github.com/Conradoou/DynamiSpectra. DynamiSpectra is also available on PyPI at https://pypi.org/project/DynamiSpectra/and can be installed using the command pip install DynamiSpectra. Example simulation data used for testing the software is available in the GitHub repository at https://github.com/Conradoou/DynamiSpectra /tree/main/data. Full API documentation, additional usage examples, and automated tests can be found on the official documentation Web site: https://conradoou.github.io/DynamiSpectra/. Unit tests can be performed using pytest, and the expected test results are available in the GitHub repository to guide users during the testing process: https://github.com/Conradoou/DynamiSpectra/tree/main/tests/Test%20results. The Web site platform: https://dynamispectra.onrender.com/.

## Notes

Interpretation guidelines from DynamiSpectra web interface; line plots of structural properties including RMSD, Rg, salt bridges, hydrogen bonds, RMSF; analysis of peptide–ligand contacts and hydrophobic interactions; ligand angle analysis; system pressure and density profiles; validation of RMSD calculations across analysis tools (PDF)
Complete documentation of the DynamiSpectra package including installation instructions, usage guidelines, and detailed description of analysis modules (PDF)
Demonstrating the usage of the DynamiSpectra package (MP4)
Demonstrating the usage of the Web site interface (MP4)
