# Supplementary material for: DynamiSpectra: A Python Software Package and Web Platform for Molecular Dynamics Data Analysis in Computational Biology
Source: J Chem Inf Model. 2025 Aug 20;65(17):8901–10. doi: 10.1021/acs.jcim.5c01270 (PMC12421658; doi:10.1021/acs.jcim.5c01270)
Supplement: Supplementary file 1 [file ci5c01270_si_001.pdf]

Supporting Information

DynamiSpectra: a Python software package and web platform for molecular dynamics data analysis in computational biology

Iverson Conrado Bezerra[1,2], Jéssika de Oliveira Viana[4], Karen Cacilda Weber[4,5], Priscila Gubert[1,2,3]*

Affiliations:

[1] Keizo Asami Institute, iLIKA, Federal University of Pernambuco, Recife, Brazil.

[2] Graduate Program in Biology Applied to Health, PPGBAS, Federal University of Pernambuco, Recife, Brazil.

[3] Graduate Program in Pure and Applied Chemistry, POSQUIPA, Federal University of Western Bahia, Bahia, Brazil.

[4] National Institute of Science and Technology on Molecular Sciences - INCT-CiMol, Department of Chemistry, Federal University of Paraíba, Paraíba, Brazil.

[5] Chemistry Department, Federal University of Paraíba, João Pessoa, Brazil.

* Corresponding author
E-mail address: priscila.gubert@ufpe.br (Priscila Gubert).
ORCID: https://orcid.org/0000-0002-3598-8050
Universidade Federal de Pernambuco. Instituto Keizo Asami (iLIKA).
Av. Prof. Moraes Rego, 1235 - Cidade Universitária, 50670-901, Recife - PE, Brazil.
+ 55 (81) 2126-8484

Table S1. Interpretation Guidelines Displayed via "Show Interpretation" Panels in the DynamiSpectra Web Interface.

Analysis	Interpretation Guideline	References
RMSD	Plateau < 0.3 nm = structural convergence; > 0.4 nm = unfolding or structural drift	10.1073/pnas.1811364115
RMSF	High RMSF > 0.3 nm suggests flexible loops/termini; core residues usually show lower values.	10.1016/j.bpj.2009.11.011
Rg	Stable Rg indicates compact structure; increasing Rg suggests expansion or unfolding.	10.3390/ma17235686
SASA	Stable SASA = structural equilibrium; rising SASA may indicate unfolding or hydration changes.	10.3389/fnmol.2022.822863
Hbond	Stable H-bonds number indicates structural stability; decreasing count may reflect unfolding or disruption of tertiary structure.	10.1073/pnas.2319094121
Salt bridge	Persistence reflects stability; disappearance suggests conformational rearrangement.	10.1002/pro.2789

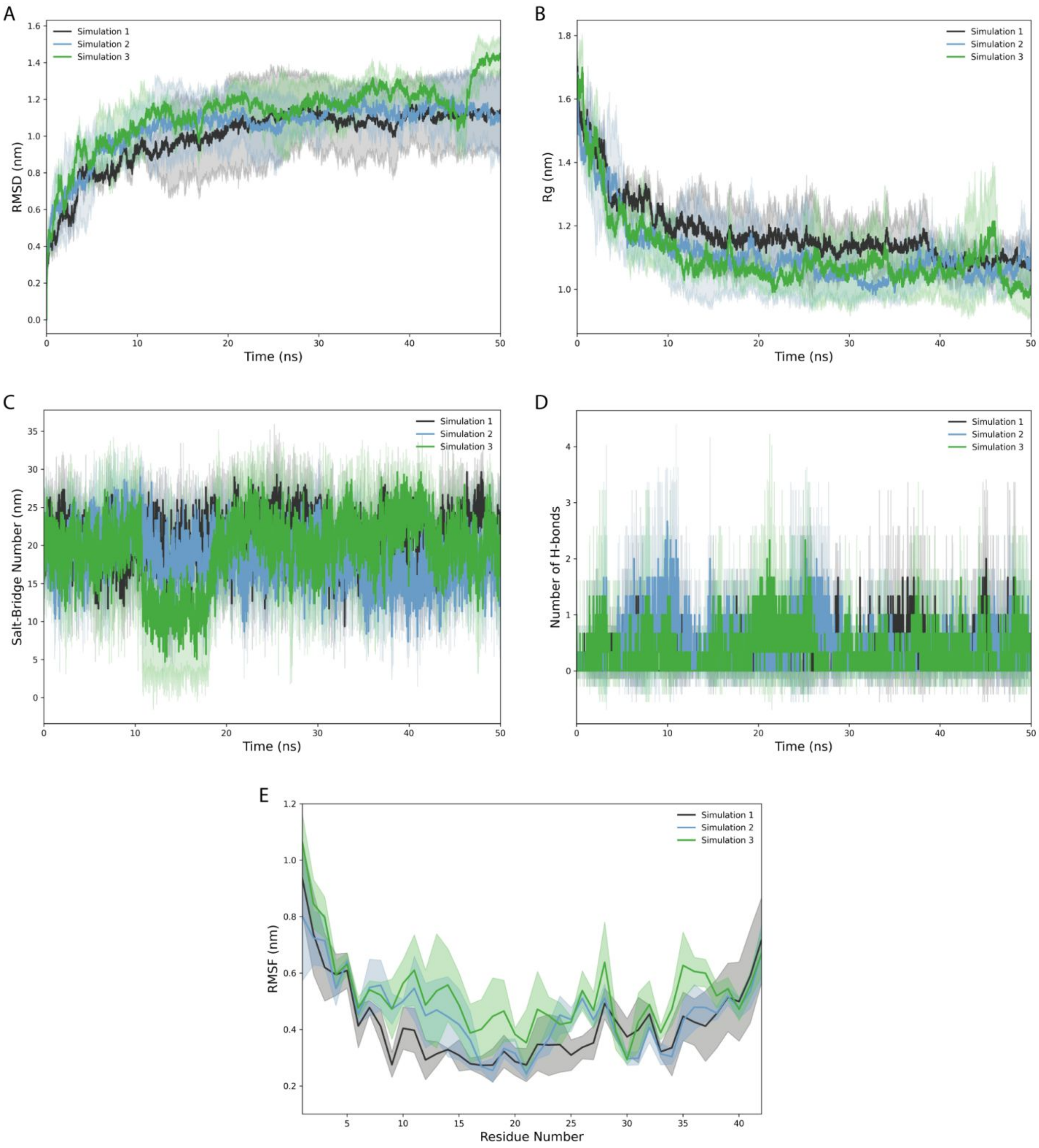

Figure S1. Line plots of structural properties along the molecular dynamics simulations. (A) RMSD; (B) Rg; (C) Number of salt bridges; (D) Number of Hbonds; and (E) RMSF. Each plot represents the average values across the three simulation replicas, and the shaded areas indicate the standard deviation.

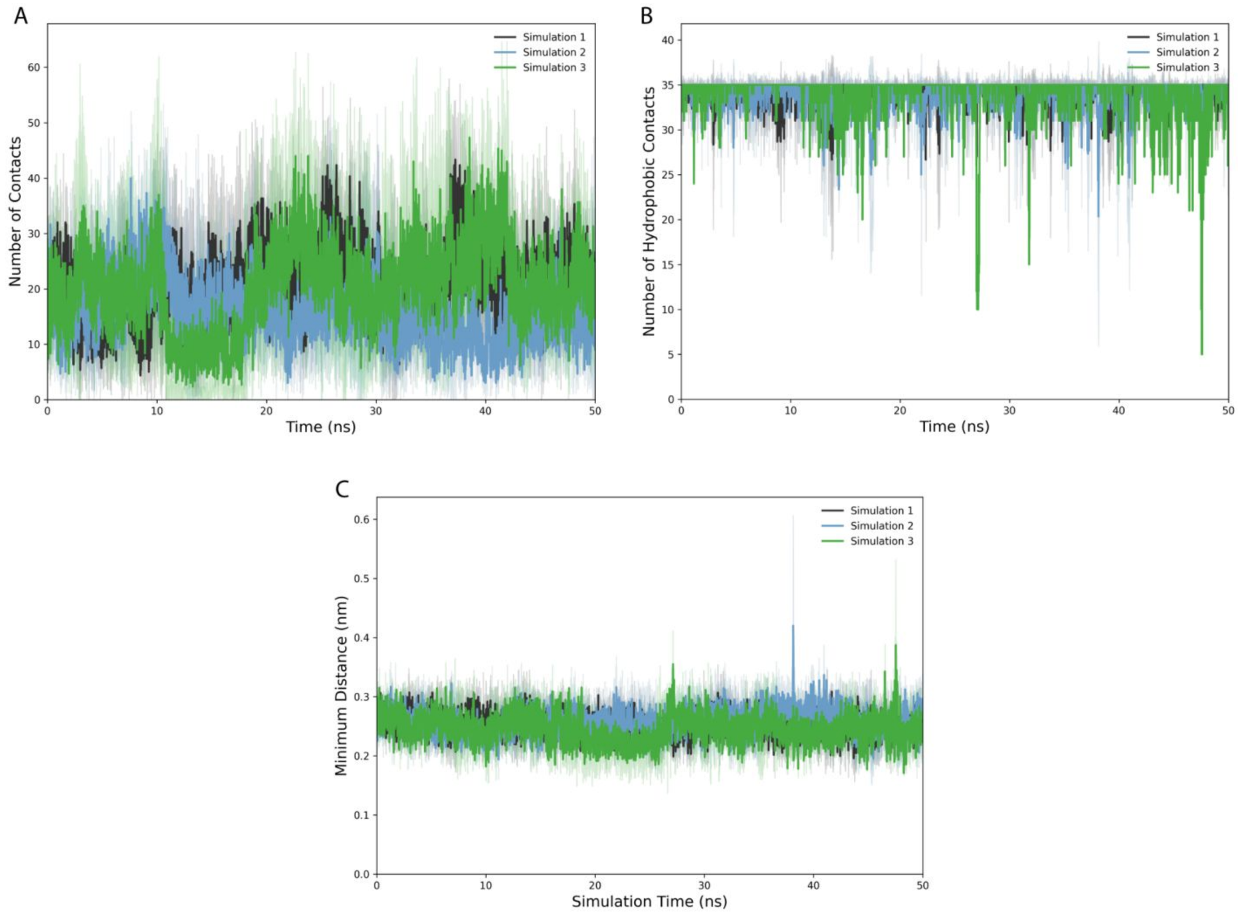

Figure S2. Analysis of peptide–ligand interactions throughout the MD simulations. (A) Number of peptide-ligand Contacts; (B) Number of hydrophobic contacts over time; (C) Minimum distance between the peptide and the ligand over time.

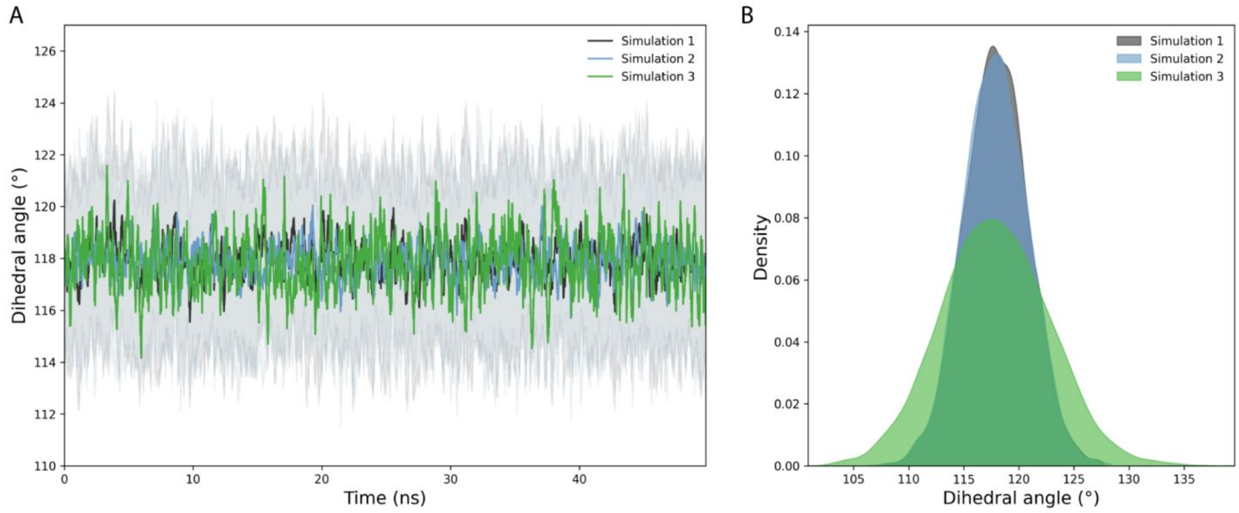

Figure S3. Analysis of the ligand angle throughout the MD simulations. (A) time-dependent plot of the ligand angle; and (B) density distribution of the ligand angle.

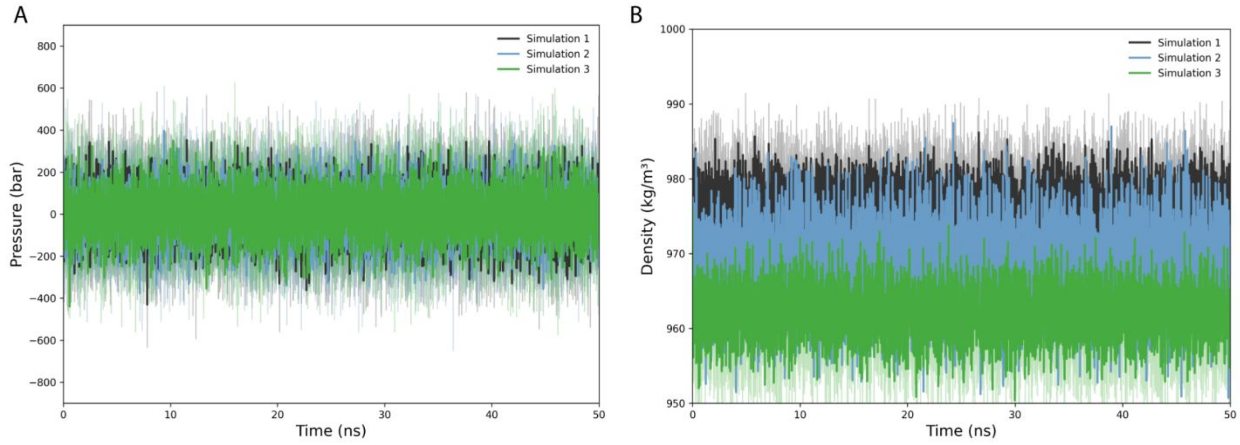

Figure S4. System properties throughout the MD simulations. (A) pressure profile over time; and (B) density profile over time.

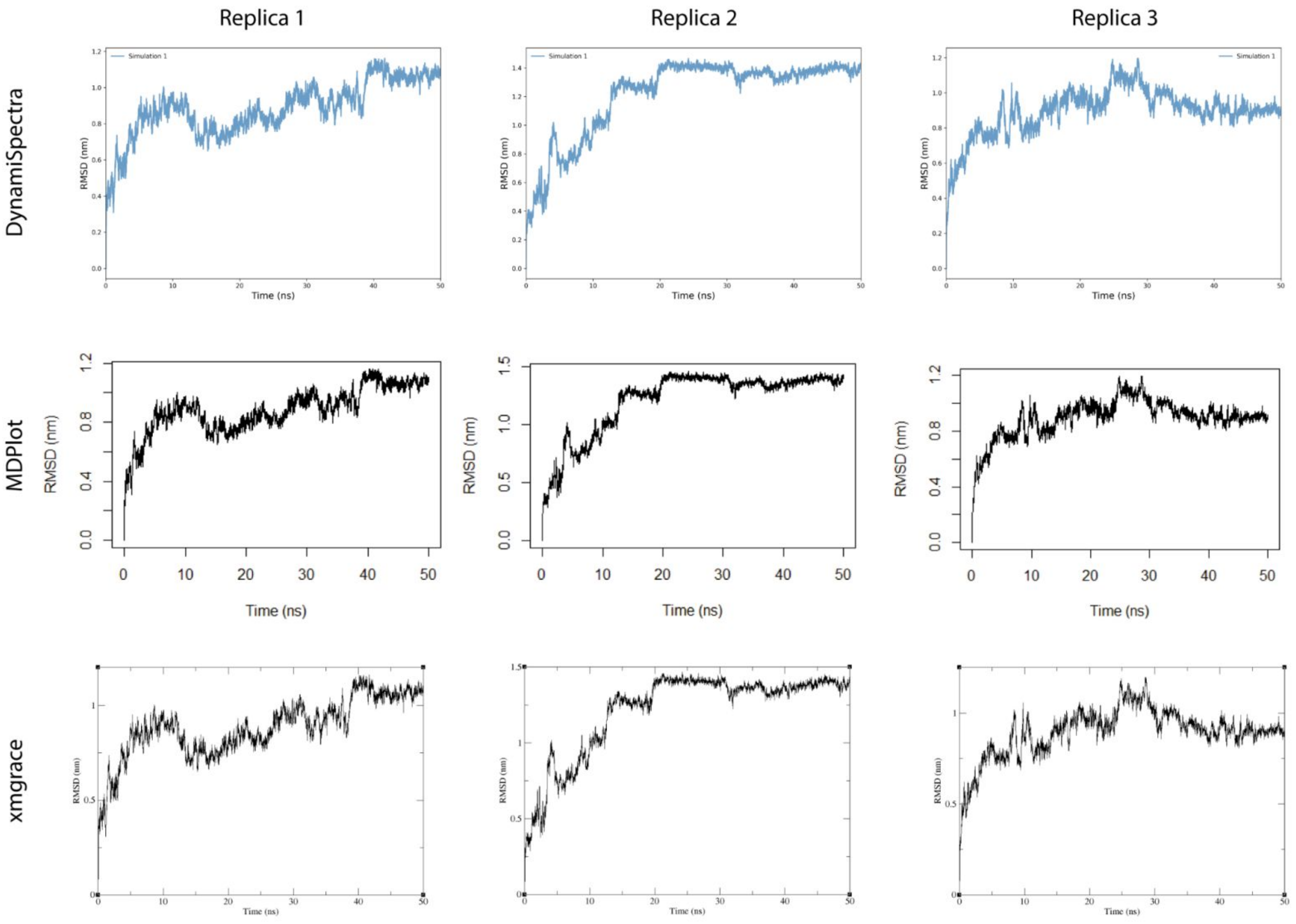

Figure S5. Validation of RMSD calculations across different analysis tools. The RMSD profiles from replicas 1, 2, and 3 of the molecular dynamics simulation were compared using DynamiSpectra, MDplot, and Xmgrace, showing consistent results across the three tools.
