# Supplementary material for: DynamiSpectra: A Python Software Package and Web Platform for Molecular Dynamics Data Analysis in Computational Biology
Source: J Chem Inf Model. 2025 Aug 20;65(17):8901–10. doi: 10.1021/acs.jcim.5c01270 (PMC12421658; doi:10.1021/acs.jcim.5c01270)
Supplement: Supplementary file 2 [file ci5c01270_si_002.pdf]

DynamiSpectra

Release 1.1.0

Iverson Conrado Bezerra

Jul 15, 2025

Contents

1 Overview **1**

1.1 DynamiSpectra: from molecular dynamics simulations to comprehensive data analysis 1
1.2 Installation 1
1.3 Documentation 1
1.4 Demo 2

2 Installation **3**

3 Running Automated Tests for DynamiSpectra **5**

3.1 1. Clone the repository 5
3.2 2. Change to the project directory 5
3.3 3. (Optional but recommended) Create and activate a virtual environment 5
3.4 4. Install required dependencies 5
3.5 5. Install DynamiSpectra in editable mode 6
3.6 6. Run the automated tests 6
3.7 7. Check the test results 6
3.8 Notes 6

4 Input **7**

4.1 Root Mean Square Deviation 7
4.2 Root Mean Square Fluctuation 9
4.3 Radius of Gyration 11
4.4 Hydrogen Bond Analysis 12
4.5 Solvent Accessible Surface Area 14
4.6 Salt Bridge 16
4.7 Protein–Ligand Contacts 18
4.8 Protein–Ligand Minimum Distance 20
4.9 Protein-Ligand Hydrophobic Contacts 21
4.10 Inter-residue Distance Matrix 23
4.11 Phi and Psi Angles 24
4.12 Rotamers (Dihedral χ1 and χ2 Angles) 25
4.13 Ligand Density 27
4.14 Ligand Dihedral Angle Analysis 27
4.15 Principal Component Analysis (PCA) 30
4.16 Secondary Structure Probability 31
4.17 Secondary Structure Fractions 32
4.18 Pressure Analysis 32
4.19 Temperature Analysis 34
4.20 Density Analysis 36

5 RMSD 39
5.1 Overview . . . 39
5.2 Complete code . . . 41

6 RMSF 45
6.1 Overview . . . 45
6.2 Complete code . . . 46

7 Radius of gyration 51
7.1 Overview . . . 51
7.2 Complete code . . . 53

8 Hydrogen Bond Analysis 57
8.1 Overview . . . 57
8.2 Complete code . . . 59

9 Solvent Accessible Surface Area 65
9.1 Overview . . . 65
9.2 Complete code . . . 67

10 Salt Bridge 71
10.1 Overview . . . 71
10.2 Complete code . . . 73

11 Protein-Ligand Contacts 77
11.1 Overview . . . 77
11.2 Complete code . . . 79

12 Protein-Ligand Minimal Distance 85
12.1 Overview . . . 85
12.2 Complete code . . . 87

13 Protein–Ligand Hydrophobic Contacts 93
13.1 Overview . . . 93
13.2 Complete code . . . 95

14 Inter-residue Distance Matrix 101
14.1 Overview . . . 101
14.2 Complete code . . . 102

15 Phi and Psi Angles 107
15.1 Overview . . . 107
15.2 Complete code . . . 108

16 Rotamers 113
16.1 Overview . . . 113
16.2 Complete code . . . 116

17 Ligand Density 121
17.1 Overview . . . 121
17.2 Complete code . . . 122

18 Ligand Angle 125
18.1 Overview . . . 125
18.2 Complete code . . . 128

19 Principal Component Analysis 133
19.1 Overview . . . 133
19.2 Complete code . . . 134

20 Secondary Structure Probability **139**
20.1 Overview . . . 139
20.2 Complete code . . . 140

21 Secondary Structure Fraction **145**
21.1 Overview . . . 145
21.2 Complete code . . . 146

22 System Pressure **151**
22.1 Overview . . . 151
22.2 Complete code . . . 153

23 System Temperature **159**
23.1 Overview . . . 159
23.2 Complete code . . . 161

24 System Density **165**
24.1 Overview . . . 165
24.2 Complete code . . . 167

25 Contributing **173**
25.1 Bug reports . . . 173
25.2 Documentation improvements . . . 173
25.3 Feature requests and feedback . . . 173

26 Authors **175**

27 Changelog **177**
27.1 1.1.0 (2025-07-08) . . . 177
27.2 1.0.6 (2025-05-01) . . . 177

28 Indices and tables **179**

1 Overview

1.1 DynamiSpectra: from molecular dynamics simulations to comprehensive data analysis

DynamiSpectra is an advanced Python package designed for analyzing molecular dynamics simulation data. Built to process and interpret results primarily from *GROMACS https://www.gromacs.org/* structure, *DynamiSpectra* provides a streamlined and efficient workflow for researchers in the field of molecular dynamics. By leveraging the power of Python, this package enables users to perform complex analyses, visualize molecular behaviors, and gain valuable insights from simulation outputs. Whether you're studying protein folding, ligand interactions, or other molecular phenomena, *DynamiSpectra* empowers you to dive deep into your data and uncover meaningful patterns.

It provides powerful tools to calculate and visualize a wide range of molecular properties, including Hydrogen bonds, Root Mean Square Deviation, Root Mean Square Fluctuation, Solvent Accessible Surface Area, Radius of Gyration, Secondary Structure Analysis, and Principal component. These features enable researchers to gain deep insights into the dynamics of molecular systems, study conformational changes, and investigate interactions between molecules with greater precision.

1.2 Installation

```
pip install DynamiSpectra
```

You can also install the in-development version with:

```
pip install https://github.com/Conradoou/DynamiSpectra/archive/main.zip
```

Note: *DynamiSpectra* uses the package SciPy, which requires a fortran compiler to be installed to build its source,which can be tricky on some systems. An alternative to this is to use Anaconda environment. *DynamiSpectra* can then be more easily installed within this environment, using the command line or preferred method. Installing *DynamiSpectra* may take some time on certain systems. If you observe the command line halting, please be patient, as the installation process may require seconds to minutes to complete.

1.3 Documentation

https://dynamiSpectra.readthedocs.io/

1.4 Demo

In the repository, specifically in DynamiSpectra/datas, there are several folders for each analysis with the corresponding GROMACS output data for testing.

2 Installation

Use the following command in the command line:

```
pip install DynamiSpectra
```

Note: ***DynamiSpectra*** **uses the SciPy package, which may require a Fortran compiler for installation if built from source.**

However, pre-compiled versions are typically available via pip, eliminating the need for manual compilation. If any installation issues arise, using an Anaconda environment can simplify the process, as SciPy and its dependencies can be easily installed through conda.

3 Running Automated Tests for DynamiSpectra

This guide explains how to run the automated tests of the DynamiSpectra package after cloning it from GitHub.

3.1 1. Clone the repository

Open your terminal (command prompt) and run:

```
git clone https://github.com/Conradoou/DynamiSpectra.git
```

This will download the full project, including the test data.

3.2 2. Change to the project directory

```
cd DynamiSpectra
```

3.3 3. (Optional but recommended) Create and activate a virtual environment

- On Linux/macOS:

```
python3 -m venv venv
source venv/bin/activate
```

- On Windows:

```
python -m venv venv
.\venv\Scripts\activate
```

3.4 4. Install required dependencies

Install all required Python packages using:

```
pip install -r requirements.txt
```

3.5 5. Install DynamiSpectra in editable mode

This allows running the package locally with the latest changes:

```
pip install -e .
```

3.6 6. Run the automated tests

Execute all tests with:

```
pytest
```

To run a specific test file, for example the pressure analysis test:

```
pytest tests/test_pressure.py
```

3.7 7. Check the test results

- If all tests pass, you will see output indicating no failures.
- If any test fails, pytest will display the error details for troubleshooting.

3.8 Notes

- The test scripts use data files inside the *data/* folder, so keep this folder intact.
- Make sure you have *pytest* installed; it is included in *requirements.txt*.
- Inside the *tests/* folder, there is a subfolder named *Test results* that contains images of the expected output plots. You can visually compare these images with the plots generated during the test runs to confirm the results.

4 Input

DynamiSpectra provides a variety of data analysis tools for GROMACS output files:

4.1 Root Mean Square Deviation

DynamiSpectra requires GROMACS output files with the *.xvg* extension for RMSD analysis. These files must contain the time series of atomic deviations during the simulation, typically generated using the *gmx rms* tool from GROMACS. The *.xvg* file is expected to have two columns: the first representing simulation time in nanoseconds (ns), and the second containing RMSD values in nanometers (nm). These values correspond to the deviation of the selected atoms or groups of atoms over time, in relation to a reference structure.

To generate this file using GROMACS, the following command can be used:

```
gmx rms -s Simulation.tpr -f Simulation.xtc -o rmsd.xvg -tu ns
```

DynamiSpectra allows the analysis of one or more simulation groups. Each group can include one or multiple replicate *.xvg* files. The analysis function automatically computes the average and standard deviation across replicates for each group and produces both time-resolved and density plots.

Code Execution

The analysis is performed by calling the *rmsd_analysis* function from the RMSD module:

```
from dynamispectra.RMSD import rmsd_analysis
```

The following example shows how to analyze three simulation groups, each containing three replicates:

```
# Input RMSD files for each simulation group
simulation1_files = [
    r'C:\Users\Conrado\Desktop\DynamiSpectra\datas\RMSD\rmsd_Replicate1.xvg',
    r'C:\Users\Conrado\Desktop\DynamiSpectra\datas\RMSD\rmsd_Replicate2.xvg',
    r'C:\Users\Conrado\Desktop\DynamiSpectra\datas\RMSD\rmsd_Replicate3.xvg'
]

simulation2_files = [
    r'C:\Users\Conrado\Desktop\DynamiSpectra\datas\RMSD\rmsd_Replicate1.xvg',
    r'C:\Users\Conrado\Desktop\DynamiSpectra\datas\RMSD\rmsd_Replicate2.xvg',
    r'C:\Users\Conrado\Desktop\DynamiSpectra\datas\RMSD\rmsd_Replicate3.xvg'
]

simulation3_files = [
    r'C:\Users\Conrado\Desktop\DynamiSpectra\datas\RMSD\rmsd_Replicate1.xvg',
```

(continues on next page)

(continued from previous page)

```
    r'C:\Users\Conrado\Desktop\DynamiSpectra\datas\RMSD\rmsd_Replicate2.xvg',
    r'C:\Users\Conrado\Desktop\DynamiSpectra\datas\RMSD\rmsd_Replicate3.xvg'
]

# Output directory to save plots
output_folder = r'C:\Users\Conrado\Documents\Test'

# Optional configuration for the RMSD vs time plot
rmsd_config = {
    # Labels for each group
    'labels': ['Simulation 1', 'Simulation 2', 'Simulation 3'],
    # Line colors for each group
    'colors': ['#333333', '#6A9EDA', '#49C547'],
    # Transparency of the shaded error area
    'alpha': 0.2,
    # Size of the figure (width, height)
    'figsize': (9, 6),
    # Label for the x-axis
    'xlabel': 'Simulation Time (ns)',
    # Label for the y-axis
    'ylabel': 'RMSD (nm)',
    # Font size for axis labels
    'label_fontsize': 12
}

# Optional configuration for the RMSD density (distribution) plot
density_config = {
    # Labels for each group
    'labels': ['Simulation 1', 'Simulation 2', 'Simulation 3'],
    # Line colors for each group
    'colors': ['#333333', '#6A9EDA', '#49C547'],
    # Transparency of the curves
    'alpha': 0.6,
    # Size of the density plot
    'figsize': (6, 6),
    # Label for the x-axis
    'xlabel': 'RMSD (nm)',
    # Label for the y-axis
    'ylabel': 'Kernel Density',
     # Font size for axis labels
    'label_fontsize': 12
}

# Run the analysis
rmsd_analysis(
    output_folder,
    simulation1_files,
    simulation2_files,
    simulation3_files,
    rmsd_config=rmsd_config,
    density_config=density_config
)
```

The *rmsd_config* and *density_config* dictionaries allow users to customize the appearance of the plots. All fields are optional, and default values will be applied if no configuration is provided.

Although the function supports multiple groups with replicates, it also works with a single group and a single replicate.

For example, calling:

```
rmsd_analysis(output_folder, simulation1_files)
```

is sufficient to generate RMSD and density plots for just one simulation set. This flexibility allows users to perform both basic and comparative analyses without modifying the source code, regardless of the number of replicates available.

4.2 Root Mean Square Fluctuation

DynamiSpectra requires GROMACS output files with the *.xvg* extension for RMSF analysis. These files must contain the root mean square fluctuation of each residue, typically generated using the *gmx rmsf* tool from GROMACS. The *.xvg* file must contain two columns: the first indicating the residue number, and the second representing the RMSF value (nm) for each residue. These values quantify the flexibility or mobility of each residue along the simulation trajectory.

To generate the required file using GROMACS, the following command can be used:

```
gmx rmsf -f Simulation.xtc -s Simulation.tpr -o rmsf.xvg -res
```

DynamiSpectra allows analyzing one or more simulation groups, each optionally containing multiple replicate *.xvg* files. The analysis function computes the average and standard deviation across replicates and generates two types of plots: a per-residue fluctuation curve and a density distribution of RMSF values.

Code Execution

Import the analysis function:

```
from dynamispectra.RMSF import rmsf_analysis
```

Example with three simulation groups, each containing three replicates:

```
# Define replicate file paths for each simulation group
simulation1_files = [
    r'C:\Users\Conrado\Desktop\DynamiSpectra\datas\RMSF\rmsf_Replicate1.xvg',
    r'C:\Users\Conrado\Desktop\DynamiSpectra\datas\RMSF\rmsf_Replicate2.xvg',
    r'C:\Users\Conrado\Desktop\DynamiSpectra\datas\RMSF\rmsf_Replicate3.xvg'
]

simulation2_files = [
    r'C:\Users\Conrado\Desktop\DynamiSpectra\datas\RMSF\rmsf_Replicate1.xvg',
    r'C:\Users\Conrado\Desktop\DynamiSpectra\datas\RMSF\rmsf_Replicate2.xvg',
    r'C:\Users\Conrado\Desktop\DynamiSpectra\datas\RMSF\rmsf_Replicate3.xvg'
]

simulation3_files = [
    r'C:\Users\Conrado\Desktop\DynamiSpectra\datas\RMSF\rmsf_Replicate1.xvg',
    r'C:\Users\Conrado\Desktop\DynamiSpectra\datas\RMSF\rmsf_Replicate2.xvg',
    r'C:\Users\Conrado\Desktop\DynamiSpectra\datas\RMSF\rmsf_Replicate3.xvg'
]

# Output directory to save the plots
output_folder = r'C:\Users\Conrado\Documents\Test'

# Optional configuration for the per-residue RMSF plot
rmsf_config = {
    # Labels for each group in the legend
    'labels': ['Simulation 1', 'Simulation 2', 'Simulation 3'],
    # Line colors for each group
```

(continues on next page)

(continued from previous page)

```
    'colors': ['#333333', '#6A9EDA', '#49C547'],
    # Transparency of the shaded error region
    'alpha': 0.3,
    # Size of the figure (width, height)
    'figsize': (9, 6),
    # Label for the x-axis
    'xlabel': 'Residue Number',
    # Label for the y-axis
    'ylabel': 'RMSF (nm)',
    # Font size for axis labels
    'label_fontsize': 12,
    # X-axis limits (optional)
    'xlim': (1, 42),
    # Y-axis limits (optional)
    'ylim': (0.1, 0.9),
}

# Optional configuration for the RMSF density plot
density_config = {
    # Labels for KDE curves
    'labels': ['Simulation 1', 'Simulation 2', 'Simulation 3'],
    # Colors for KDE curves
    'colors': ['#333333', '#6A9EDA', '#49C547'],
    # Transparency of the KDE fill
    'alpha': 0.5,
    # Size of the density figure
    'figsize': (6, 6),
    # X-axis label
    'xlabel': 'RMSF (nm)',
    # Y-axis label
    'ylabel': 'Density',
    # Font size for axis labels
    'label_fontsize': 12,
    # X-axis limits (optional)
    'xlim': (0, 1),
    # Y-axis limits (None = automatic)
    'ylim': None,
}

# Run the RMSF analysis
rmsf_analysis(
    output_folder,
    simulation1_files,
    simulation2_files,
    simulation3_files,
    rmsf_config=rmsf_config,
    density_config=density_config
)
```

You may also analyze a single group with a single replicate by passing only one list of files:

```
rmsf_analysis(output_folder, simulation1_files)
```

Both configuration dictionaries (*rmsf_config* and *density_config*) are optional. If not provided, the function will generate plots with default layout and colors. This flexibility allows users to customize plots according to their preferences while keeping the function compatible with both simple and advanced use cases.

4.3 Radius of Gyration

DynamiSpectra requires GROMACS output files with the *.xvg* extension for Radius of Gyration (Rg) analysis. These files should contain the time series of Rg values computed during the simulation, typically generated with the *gmx gyrate* tool. The *.xvg* file must include the simulation time in nanoseconds (ns) in the first column and the total radius of gyration in nanometers (nm) in the second column. Additional columns (typically third to fifth) may include component values along the X, Y, and Z axes, though only the total Rg (second column) is used in this analysis.

To generate a compatible file using GROMACS, the following command can be used:

```
gmx gyrate -f Simulation.xtc -s Simulation.tpr -o gyrate.xvg
```

The analysis supports one or more simulation groups, each potentially composed of multiple replicate *.xvg* files. *DynamiSpectra* calculates average and standard deviation across replicates for each group, and produces both a time-resolved plot and a distribution (KDE) plot of the Rg values.

Code Execution

Import the analysis function from the Rg module:

```
from dynamispectra.Rg import rg_analysis
```

Example using three simulation groups with three replicates each:

```
# Define replicate files for each simulation group
simulation1_files = [
    r'C:\Users\Conrado\Desktop\DynamiSpectra\datas\Rg\gyrate_Replicate1.xvg',
    r'C:\Users\Conrado\Desktop\DynamiSpectra\datas\Rg\gyrate_Replicate2.xvg',
    r'C:\Users\Conrado\Desktop\DynamiSpectra\datas\Rg\gyrate_Replicate3.xvg'
]

simulation2_files = [
    r'C:\Users\Conrado\Desktop\DynamiSpectra\datas\Rg\gyrate_Replicate1.xvg',
    r'C:\Users\Conrado\Desktop\DynamiSpectra\datas\Rg\gyrate_Replicate2.xvg',
    r'C:\Users\Conrado\Desktop\DynamiSpectra\datas\Rg\gyrate_Replicate3.xvg'
]

simulation3_files = [
    r'C:\Users\Conrado\Desktop\DynamiSpectra\datas\Rg\gyrate_Replicate1.xvg',
    r'C:\Users\Conrado\Desktop\DynamiSpectra\datas\Rg\gyrate_Replicate2.xvg',
    r'C:\Users\Conrado\Desktop\DynamiSpectra\datas\Rg\gyrate_Replicate3.xvg'
]

# Output directory to save the plots
output_folder = r'C:\Users\Conrado\Documents\Test'

# Configuration for the Rg time series plot
rg_config = {
    # Legend labels for each simulation group
    'labels': ['Simulation 1', 'Simulation 2', 'Simulation 3'],
    # Line and fill colors
    'colors': ['#333333', '#6A9EDA', '#49C547'],
    # Transparency for shaded error band
    'alpha': 0.2,
    # Width and height of the figure
    'figsize': (9, 6),
     # Label for the x-axis
    'xlabel': 'Simulation Time (ns)',
    # Label for the y-axis
```

(continues on next page)

(continued from previous page)

```
    'ylabel': 'Rg (nm)',
    # Font size for axis labels
    'label_fontsize': 12
}

# Configuration for the Rg distribution (KDE) plot
density_config = {
    # Labels matching each simulation group
    'labels': ['Simulation 1', 'Simulation 2', 'Simulation 3'],
    # Fill colors for density curves
    'colors': ['#333333', '#6A9EDA', '#49C547'],
    # Transparency for KDE fill
    'alpha': 0.6,
    # Width and height of the KDE figure
    'figsize': (6, 6),
    # Label for the x-axis
    'xlabel': 'Rg (nm)',
    # Label for the y-axis
    'ylabel': 'Kernel Density',
    # Font size for axis labels
    'label_fontsize': 12
}

# Run the Rg analysis and generate both plots
rg_analysis(
    output_folder,
    simulation1_files,
    simulation2_files,
    simulation3_files,
    rg_config=rg_config,
    density_config=density_config
)
```

It is also possible to run the analysis using a single group with only one replicate. For example:

```
rg_analysis(output_folder, simulation1_files)
```

All plot configuration dictionaries are optional. If omitted, the plots will be generated with default visual settings. This design allows users to tailor the visualization for presentations or publications without altering the analysis logic.

4.4 Hydrogen Bond Analysis

DynamiSpectra requires *.xvg* output files from GROMACS for hydrogen bond analysis. These files are typically generated using the *gmx hbond* tool and must contain the time-resolved data of H-bond interactions observed during molecular dynamics simulations.

The *.xvg* file must have the following structure:

- Column 1: Simulation time in nanoseconds
- Column 2: Total number of hydrogen bonds detected at each time point
- Column 3: Number of atom pairs within 0.35 nm (potential H-bond candidates)

These data enable detailed tracking of H-bond dynamics and the compactness or rearrangement of molecular systems throughout the simulation.

To generate the required *.xvg* file using GROMACS:

```
gmx hbond -s Simulation.tpr -f Simulation.xtc -num hbond.xvg -tu ns
```

DynamiSpectra supports analysis of one or multiple simulation groups, each optionally including several replicate files. It computes the average and standard deviation of H-bond counts across replicates and generates both time-resolved and density plots.

Code Execution

Import the H-bond analysis function:

```
from dynamispectra.Hbond import hbond_analysis
```

Example using three simulation groups:

```
# Define paths to .xvg replicate files for each simulation group
simulation1_files = [
    r'C:\Users\Conrado\Desktop\DynamiSpectra\datas\Hbond\hbond_Replicate1.xvg',
    r'C:\Users\Conrado\Desktop\DynamiSpectra\datas\Hbond\hbond_Replicate2.xvg',
    r'C:\Users\Conrado\Desktop\DynamiSpectra\datas\Hbond\hbond_Replicate3.xvg'
]

simulation2_files = [
    r'C:\Users\Conrado\Desktop\DynamiSpectra\datas\Hbond\hbond_Replicate1.xvg',
    r'C:\Users\Conrado\Desktop\DynamiSpectra\datas\Hbond\hbond_Replicate2.xvg',
    r'C:\Users\Conrado\Desktop\DynamiSpectra\datas\Hbond\hbond_Replicate3.xvg'
]

simulation3_files = [
    r'C:\Users\Conrado\Desktop\DynamiSpectra\datas\Hbond\hbond_Replicate1.xvg',
    r'C:\Users\Conrado\Desktop\DynamiSpectra\datas\Hbond\hbond_Replicate2.xvg',
    r'C:\Users\Conrado\Desktop\DynamiSpectra\datas\Hbond\hbond_Replicate3.xvg'
]

# Folder where the plots will be saved
output_folder = r'C:\Users\Conrado\Documents\Test'

# Plot configuration for H-bond over time
hbond_config = {
    # Legend labels for each simulation
    'labels': ['Simulation 1', 'Simulation 2', 'Simulation 3'],
    # Line and fill colors for each group
    'colors': ['#333333', '#6A9EDA', '#49C547'],
    # Transparency for shaded error area
    'alpha': 0.2,
    # Width and height of the time series figure
    'figsize': (9, 6),
    # Label for the x-axis
    'xlabel': 'Time (ns)',
    # Label for the y-axis
    'ylabel': 'Number of H-bonds',
    # Font size of axis labels
    'label_fontsize': 12
}

# Plot configuration for H-bond density (KDE)
density_config = {
    # KDE labels
    'labels': ['Simulation 1', 'Simulation 2', 'Simulation 3'],
```

(continues on next page)

(continued from previous page)

```
    # KDE fill colors
    'colors': ['#333333', '#6A9EDA', '#49C547'],
    # Transparency for KDE areas
    'alpha': 0.6,
    # Width and height of the KDE figure
    'figsize': (6, 6),
    # x-axis label
    'xlabel': 'Number of H-bonds',
    # y-axis label
    'ylabel': 'Kernel Density',
    # Font size for axis labels
    'label_fontsize': 12
}

# Run the H-bond analysis
hbond_analysis(
    output_folder,
    simulation1_files,
    simulation2_files,
    simulation3_files,
    hbond_config=hbond_config,
    density_config=density_config
)
```

You can also run the analysis with a single group and a single replicate:

```
hbond_analysis(output_folder, simulation1_files)
```

If configuration dictionaries are omitted, *DynamiSpectra* will generate the plots with default settings. These customization options allow full control of the appearance for publications or presentations.

4.5 Solvent Accessible Surface Area

DynamiSpectra requires *.xvg* output files from GROMACS for SASA analysis. These files are typically generated using the *gmx sasa* tool and provide time-resolved measurements of the solvent-accessible surface area of the selected atoms during the simulation.

The *.xvg* file must follow this format:

- Column 1: Simulation time (ps)
- Column 2: Total SASA (nm^2) of the selected group of atoms over time

This information enables detailed evaluation of conformational changes and solvation properties of the system.

To generate the required *.xvg* file using GROMACS:

```
gmx sasa -s Simulation.tpr -f Simulation.xtc -o sasa.xvg -probe 0.14
```

DynamiSpectra supports multiple simulation groups, each with one or more replicates. The package automatically aligns time series, computes mean and standard deviation, and generates plots for both time evolution and density distribution of SASA.

Code Execution

Import the SASA analysis module:

```
from dynamispectra.SASA import sasa_analysis
```

Example with three groups of simulations, each containing three replicates:

```
# Define paths to .xvg replicate files for each simulation group
simulation1_files = [
    r'C:\Users\Conrado\Desktop\DynamiSpectra\data\SASA\sasa_Replicate1.xvg',
    r'C:\Users\Conrado\Desktop\DynamiSpectra\data\SASA\sasa_Replicate2.xvg',
    r'C:\Users\Conrado\Desktop\DynamiSpectra\data\SASA\sasa_Replicate3.xvg'
]

simulation2_files = [
    r'C:\Users\Conrado\Desktop\DynamiSpectra\data\SASA\sasa_Replicate1.xvg',
    r'C:\Users\Conrado\Desktop\DynamiSpectra\data\SASA\sasa_Replicate2.xvg',
    r'C:\Users\Conrado\Desktop\DynamiSpectra\data\SASA\sasa_Replicate3.xvg'
]

simulation3_files = [
    r'C:\Users\Conrado\Desktop\DynamiSpectra\data\SASA\sasa_Replicate1.xvg',
    r'C:\Users\Conrado\Desktop\DynamiSpectra\data\SASA\sasa_Replicate2.xvg',
    r'C:\Users\Conrado\Desktop\DynamiSpectra\data\SASA\sasa_Replicate3.xvg'
]

# Output folder where the plots will be saved
output_folder = r'C:\Users\Conrado\Documents\Test'

# Configuration for the SASA time series plot
sasa_config = {
    # Labels shown in legend
    'labels': ['Simulation 1', 'Simulation 2', 'Simulation 3'],
    # Colors for curves and shaded area
    'colors': ['#333333', '#6A9EDA', '#49C547'],
    # Transparency of std deviation fill
    'alpha': 0.2,
    # Size of the time series figure
    'figsize': (9, 6),
    # Label for x-axis
    'xlabel': 'Simulation Time (ns)',
    # Label for y-axis
    'ylabel': 'SASA (nm²)',
    # Font size for axis labels
    'label_fontsize': 12
}

# Configuration for the SASA density distribution plot (KDE)
density_config = {
    # Legend labels for KDE plot
    'labels': ['Simulation 1', 'Simulation 2', 'Simulation 3'],
    # Fill colors for KDE areas
    'colors': ['#333333', '#6A9EDA', '#49C547'],
    # Transparency for filled areas
    'alpha': 0.6,
    # Size of the density plot
    'figsize': (6, 6),
    # x-axis label
    'xlabel': 'SASA (nm²)',
    # y-axis label
    'ylabel': 'Kernel Density',
    # Font size for axis labels
    'label_fontsize': 12
}
```

(continues on next page)

(continued from previous page)

```
# Run the analysis for SASA
sasa_analysis(
    output_folder,
    simulation1_files,
    simulation2_files,
    simulation3_files,
    sasa_config=sasa_config,
    density_config=density_config
)
```

To run the analysis with a single group or a single file, just provide one list:

```
sasa_analysis(output_folder, simulation1_files)
```

If no configuration dictionaries are provided, default settings will be used. Customize the plots as needed to match the desired presentation style or publication format.

4.6 Salt Bridge

DynamiSpectra requires *.xvg* output files from GROMACS for salt bridge analysis between the ligand and the protein, or only protein. These files are typically generated using the *gmx mindist* tool with appropriate index groups and contain time series of minimum distances between charged groups.

The *.xvg* file must follow this format:

- Column 1: Simulation time (ps)
- Column 2: Minimum salt-bridge distance (nm) between charged groups over time

This data allows monitoring the presence or absence of salt-bridge interactions during the simulation.

To generate the required *.xvg* file using GROMACS:

```
gmx mindist -f Simulation.xtc -s Simulation.tpr -n index.ndx -od Simulation.xvg -
↪on saltbridge.xvg -group -d 0.4
```

DynamiSpectra supports multiple simulation groups, each with one or more replicates. The package automatically aligns time series, computes mean and standard deviation, and generates plots for both time evolution and density distribution of salt-bridge distances.

Code Execution

Import the salt bridge analysis module:

```
from dynamispectra.saltbridge import saltbridge_analysis
```

Example with three groups of simulations, each containing three replicates:

```
# Define paths to .xvg replicate files for each simulation group
simulation1_files = [
    r'C:\Users\Conrado\Desktop\DynamiSpectra\saltbridge_310k_Replicate1.xvg',
    r'C:\Users\Conrado\Desktop\DynamiSpectra\saltbridge_310k_Replicate2.xvg',
    r'C:\Users\Conrado\Desktop\DynamiSpectra\saltbridge_310k_Replicate3.xvg'
]

simulation2_files = [
    r'C:\Users\Conrado\Desktop\DynamiSpectra\saltbridge_310k_Replicate1.xvg',
    r'C:\Users\Conrado\Desktop\DynamiSpectra\saltbridge_310k_Replicate2.xvg',
    r'C:\Users\Conrado\Desktop\DynamiSpectra\saltbridge_310k_Replicate3.xvg'
```

(continues on next page)

(continued from previous page)

```
]

simulation3_files = [
    r'C:\Users\Conrado\Desktop\DynamiSpectra\saltbridge_310k_Replicate1.xvg',
    r'C:\Users\Conrado\Desktop\DynamiSpectra\saltbridge_310k_Replicate2.xvg',
    r'C:\Users\Conrado\Desktop\DynamiSpectra\saltbridge_310k_Replicate3.xvg'
]

# Output folder where the plots will be saved
output_folder = r'C:\Users\Conrado\Documents\Test'

# Configuration for the salt-bridge time series plot
saltbridge_config = {
    # Labels shown in legend
    'labels': ['Simulation 1', 'Simulation 2', 'Simulation 3'],
    # Colors for curves and shaded area
    'colors': ['#333333', '#6A9EDA', '#49C547'],
    # Transparency of std deviation fill
    'alpha': 0.2,
    # Size of the time series figure
    'figsize': (9, 6),
    # Label for x-axis
    'xlabel': 'Simulation Time (ns)',
    # Label for y-axis
    'ylabel': 'Salt-Bridge Distance (nm)',
    # Font size for axis labels
    'label_fontsize': 12
}

# Configuration for the salt-bridge density distribution plot (KDE)
density_config = {
    # Legend labels for KDE plot
    'labels': ['Simulation 1', 'Simulation 2', 'Simulation 3'],
    # Fill colors for KDE areas
    'colors': ['#333333', '#6A9EDA', '#49C547'],
    # Transparency for filled areas
    'alpha': 0.6,
    # Size of the density plot
    'figsize': (6, 6),
    # x-axis label
    'xlabel': 'Salt-Bridge Distance (nm)',
    # y-axis label
    'ylabel': 'Kernel Density',
    # Font size for axis labels
    'label_fontsize': 12
}

# Run the analysis for salt-bridge distances
saltbridge_analysis(
    output_folder,
    simulation1_files,
    simulation2_files,
    simulation3_files,
    saltbridge_config=saltbridge_config,
    density_config=density_config
)
```

To run the analysis with a single group or a single file, just provide one list:

```
saltbridge_analysis(output_folder, simulation1_files)
```

If no configuration dictionaries are provided, default settings will be used. Customize the plots as needed to match your presentation or publication style.

4.7 Protein–Ligand Contacts

DynamiSpectra requires *.xvg* output files from GROMACS for analyzing the number of atomic contacts between protein and ligand during the simulation. These files are typically generated using the *gmx mindist* or *gmx contact* tool with appropriate index groups for the protein and ligand atoms.

The *.xvg* file must follow this format:

- Column 1: Simulation time (ps)
- Column 2: Number of contacts between the selected groups over time

This information allows monitoring interaction stability, binding dynamics, and fluctuation of protein–ligand contacts across time.

To generate the required *.xvg* file using GROMACS:

```
gmx mindist -f Simulation.xtc -s Simulation.tpr -n index.ndx -on contacts.xvg -d 0.
↪35
```

DynamiSpectra supports multiple simulation groups, each with one or more replicates. The package automatically aligns time series, computes mean and standard deviation, and generates plots for both time evolution and density distribution of contacts.

Code Execution

Import the contact analysis module:

```
from dynamispectra.Contacts import contact_analysis
```

Example with three groups of simulations, each containing three replicates:

```
# Define paths to .xvg replicate files for each simulation group
simulation1_files = [
    r'C:\Users\Conrado\Desktop\DynamiSpectra\datas\contacts_Replicate1.xvg',
    r'C:\Users\Conrado\Desktop\DynamiSpectra\datas\contacts_Replicate2.xvg',
    r'C:\Users\Conrado\Desktop\DynamiSpectra\datas\contacts_Replicate3.xvg'
]

simulation2_files = [
    r'C:\Users\Conrado\Desktop\DynamiSpectra\datas\contacts_Replicate1.xvg',
    r'C:\Users\Conrado\Desktop\DynamiSpectra\datas\contacts_Replicate2.xvg',
    r'C:\Users\Conrado\Desktop\DynamiSpectra\datas\contacts_Replicate3.xvg'
]

simulation3_files = [
    r'C:\Users\Conrado\Desktop\DynamiSpectra\datas\contacts_Replicate1.xvg',
    r'C:\Users\Conrado\Desktop\DynamiSpectra\datas\contacts_Replicate2.xvg',
    r'C:\Users\Conrado\Desktop\DynamiSpectra\datas\contacts_Replicate3.xvg'
]

# Output folder where the plots will be saved
output_folder = r'C:\Users\Conrado\Documents\Test'
```

(continues on next page)

(continued from previous page)

```
# Configuration for the contact vs. time plot
contact_config = {
    # Legend labels
    'labels': ['Simulation 1', 'Simulation 2', 'Simulation 3'],
    # Curve and fill colors
    'colors': ['#333333', '#6A9EDA', '#49C547'],
    # Shaded area transparency
    'alpha': 0.2,
    # Figure size (width, height)
    'figsize': (9, 6),
    # x-axis label
    'xlabel': 'Time (ns)',
    # y-axis label
    'ylabel': 'Number of Contacts',
    # Font size for labels
    'label_fontsize': 12
}

# Configuration for the contact density (KDE) plot
density_config = {
    # Legend labels for KDE
    'labels': ['Simulation 1', 'Simulation 2', 'Simulation 3'],
    # KDE fill colors
    'colors': ['#333333', '#6A9EDA', '#49C547'],
    # Transparency for density
    'alpha': 0.2,
    # Size of the density plot
    'figsize': (9, 6),
    # x-axis label
    'xlabel': 'Number of Contacts',
    # y-axis label
    'ylabel': 'Kernel Density',
    # Font size for labels
    'label_fontsize': 12
}

# Run the contact analysis
contact_analysis(
    output_folder,
    simulation1_files,
    simulation2_files,
    simulation3_files,
    contact_config=contact_config,
    density_config=density_config
)
```

To run the analysis with a single group or a single file, just provide one list:

```
contact_analysis(output_folder, simulation1_files)
```

If no configuration dictionaries are provided, default settings will be used. Customize the plots to suit your analysis or publication needs.

4.8 Protein–Ligand Minimum Distance

DynamiSpectra requires *.xvg* output files from GROMACS to analyze the minimum distance between protein and ligand atoms during molecular dynamics simulations. These files are typically generated using the *gmx distance* tool with selected atom groups from an index file.

The *.xvg* file must follow this format:

- Column 1: Simulation time (ps)
- Column 2: Minimum distance (nm) between protein and ligand atoms at each time point

This analysis allows the evaluation of binding stability, complex dissociation, and proximity of interaction over time.

To generate the required *.xvg* file using GROMACS:

```
gmx mindist -f Simulation.xtc -s Simulation.tpr -n index.ndx -od mindist.xvg -d 0.
↪35
```

DynamiSpectra supports multiple simulation groups, each with one or more replicates. The tool aligns all time series, computes mean and standard deviation, and generates plots showing time evolution and the distribution of distances.

Code Execution

Import the distance analysis module:

```
from dynamispectra.Distance import distance_analysis
```

Example with three simulation groups, each containing three replicates:

```
# Define paths to .xvg replicate files for each simulation group
simulation1_files = [
    r'C:\Users\Conrado\Desktop\DynamiSpectra\distance_Replicate1.xvg',
    r'C:\Users\Conrado\Desktop\DynamiSpectra\distance_Replicate2.xvg',
    r'C:\Users\Conrado\Desktop\DynamiSpectra\distance_Replicate3.xvg'
]

simulation2_files = [
    r'C:\Users\Conrado\Desktop\DynamiSpectra\distance_Replicate1.xvg',
    r'C:\Users\Conrado\Desktop\DynamiSpectra\distance_Replicate2.xvg',
    r'C:\Users\Conrado\Desktop\DynamiSpectra\distance_Replicate3.xvg'
]

simulation3_files = [
    r'C:\Users\Conrado\Desktop\DynamiSpectra\distance_Replicate1.xvg',
    r'C:\Users\Conrado\Desktop\DynamiSpectra\distance_Replicate2.xvg',
    r'C:\Users\Conrado\Desktop\DynamiSpectra\distance_Replicate3.xvg'
]

# Output folder for saving plots and results
output_folder = r'C:\Users\Conrado\Documents\Test'

# Configuration for the distance time series plot
distance_config = {
    # Legend labels for each simulation group
    'labels': ['Simulation 1', 'Simulation 2', 'Simulation 3'],
    # Line colors for the plots
    'colors': ['#333333', '#6A9EDA', '#49C547'],
    # Transparency of shaded area (std deviation)
    'alpha': 0.2,
    # Width and height of the time series plot
```

(continues on next page)

(continued from previous page)

```
    'figsize': (9, 6),
    # X-axis label
    'xlabel': 'Simulation Time (ns)',
    # Y-axis label
    'ylabel': 'Minimum Distance (nm)',
    # Font size for axis labels
    'label_fontsize': 12
}

# Configuration for the distance density distribution (KDE) plot
density_config = {
    # Legend labels for KDE
    'labels': ['Simulation 1', 'Simulation 2', 'Simulation 3'],
    # Fill colors for each simulation group
    'colors': ['#333333', '#6A9EDA', '#49C547'],
    # Transparency of KDE fill
    'alpha': 0.6,
    # Width and height of the KDE plot
    'figsize': (6, 6),
    # X-axis label
    'xlabel': 'Minimum Distance (nm)',
    # Y-axis label
    'ylabel': 'Kernel Density',
    # Font size for labels
    'label_fontsize': 12
}

# Run the analysis for minimum distance
distance_analysis(
    output_folder,
    simulation1_files,
    simulation2_files,
    simulation3_files,
    distance_config=distance_config,
    density_config=density_config
)
```

To run the analysis with a single group or a single file, just provide one list:

```
distance_analysis(output_folder, simulation1_files)
```

If no configuration dictionaries are provided, default plot settings will be applied. You can tailor the plot appearance for presentations or publications by adjusting the dictionary values.

4.9 Protein-Ligand Hydrophobic Contacts

DynamiSpectra supports the analysis of hydrophobic contacts between ligand and protein from *.xvg* files. These files typically represent the number of hydrophobic contacts over time, generated using custom GROMACS selections involving hydrophobic atoms.

The *.xvg* file must follow this format:

- Column 1: Simulation time (ps)
- Column 2: Number of hydrophobic contacts between ligand and protein

This analysis helps to evaluate the contribution of nonpolar interactions to the stability and binding of protein–ligand complexes.

To generate *.xvg* files, you can use GROMACS with *gmx select* or other post-processing tools with appropriate selections:

```
gmx select -f Simulation.xtc -s Simulation.tpr -n index.ndx -select 'group "LIG"␣
↪and within 0.6 of group "Protein"' -os hydrophobic_contacts.xvg
```

Code Execution

Import the analysis function:

```
from dynamispectra.Hydrophobic_contacts import hydrophobic_analysis
```

Example with three simulation groups, each with three replicate files:

```
simulation1_files = [
    r'E:\Conrado\PNU\Dinamica\hydrophobic_contacts_Rep1.xvg',
    r'E:\Conrado\PNU\Dinamica\hydrophobic_contacts_Rep2.xvg',
    r'E:\Conrado\PNU\Dinamica\hydrophobic_contacts_Rep3.xvg'
]

simulation2_files = [
    r'E:\Conrado\PNU\Dinamica\hydrophobic_contacts2_Rep1.xvg',
    r'E:\Conrado\PNU\Dinamica\hydrophobic_contacts2_Rep2.xvg',
    r'E:\Conrado\PNU\Dinamica\hydrophobic_contacts2_Rep3.xvg'
]

simulation3_files = [
    r'E:\Conrado\PNU\Dinamica\hydrophobic_contacts3_Rep1.xvg',
    r'E:\Conrado\PNU\Dinamica\hydrophobic_contacts3_Rep2.xvg',
    r'E:\Conrado\PNU\Dinamica\hydrophobic_contacts3_Rep3.xvg'
]

output_folder = r'C:\Users\Conrado\Documents\Test'

contact_config = {
    # Legend labels for each simulation group
    'labels': ['Simulation 1', 'Simulation 2', 'Simulation 3'],
    # Colors for curves and shaded area
    'colors': ['#333333', '#6A9EDA', '#49C547'],
    # Transparency of std deviation fill
    'alpha': 0.2,
    # Figure size for time series
    'figsize': (9, 6),
    # X-axis label
    'xlabel': 'Time (ns)',
    # Y-axis label
    'ylabel': 'Number of Hydrophobic Contacts',
    # Font size for axis labels
    'label_fontsize': 12
}

density_config = {
    # Labels for KDE curves
    'labels': ['Simulation 1', 'Simulation 2', 'Simulation 3'],
    # Fill colors for KDE
    'colors': ['#333333', '#6A9EDA', '#49C547'],
    # Transparency of fill
    'alpha': 0.2,
    # Size of the KDE plot
```

(continues on next page)

(continued from previous page)

```
    'figsize': (6, 6),
    # X-axis label
    'xlabel': 'Number of Hydrophobic Contacts',
    # Y-axis label
    'ylabel': 'Kernel Density',
    # Font size for axis labels
    'label_fontsize': 12
}

hydrophobic_analysis(
    output_folder,
    simulation1_files,
    simulation2_files,
    simulation3_files,
    contact_config=contact_config,
    density_config=density_config
)
```

To run the analysis with only one group or a single file, simply provide one list:

```
hydrophobic_analysis(output_folder, simulation1_files)
```

If the configuration dictionaries are not provided, default plotting settings will be used.

4.10 Inter-residue Distance Matrix

DynamiSpectra supports the visualization of inter-residue distance matrices using *.xpm* files generated by GROMACS. These matrices represent the average distances between residue pairs throughout a trajectory and are useful for identifying compact or flexible regions within a protein.

The *.xpm* file can be generated using the following GROMACS command:

```
gmx mdmat -f Simulation.xtc -s Simulation.tpr -mean Simulation.xpm -no Simulation.
↪xvg
```

This analysis is typically used for all-against-all residue contact distances in a protein structure.

Code Execution

Import the distance matrix analysis module:

```
from dynamispectra.DistanceMatrix import distance_matrix_analysis
```

Example for one *.xpm* file corresponding to a single simulation or replicate:

```
xpm_file_path = r'C:\Users\Conrado\Documents\Test\mdmat.xpm'

output_path = r'C:\Users\Conrado\Documents\Test\distance_matrix'

config = {
    # Label for the X-axis
    'xlabel': 'Residues',
    # Label for the Y-axis
    'ylabel': 'Residues',
    # Font size for axis labels
    'label_fontsize': 12,
    # Title of the matrix plot
    'title': 'Distance Matrix - Replica 1',
```

(continues on next page)

(continued from previous page)

```
    # Font size for the plot title
    'title_fontsize': 12,
    # Label for the color scale
    'colorbar_label': 'Distance (nm)',
    # Maximum distance value (cmap upper limit)
    'max_distance': 2.0,
    # Colormap (e.g., 'jet', 'viridis', 'plasma')
    'cmap': 'jet'
}

distance_matrix_analysis(
    xpm_file_path,
    output_path,
    plot=True,
    config=config
)
```

The resulting plot will be saved as *.png* and *.tiff* using the specified *output_path* as prefix. The matrix is rendered as a color-coded heatmap where the color intensity reflects inter-residue distances.

To use default plotting parameters, omit the *config* argument:

```
distance_matrix_analysis(xpm_file_path, output_path)
```

4.11 Phi and Psi Angles

DynamiSpectra analyzes backbone dihedral angles φ (phi) and ψ (psi) from .xvg files generated by GROMACS. These files contain Ramachandran angle distributions used to assess protein conformational states over time. The analysis produces individual plots for each simulation replica as well as a combined plot representing the circular average of all replicas.

The *.xvg* files can be generated with the following GROMACS command:

```
gmx rama -s Simulation.tpr -f Simulation.xtc -o rama.xvg
```

This analysis creates kernel density estimation (KDE) heatmaps of φ-ψ angle distributions, helping identify favored secondary structure conformations.

Code Execution

Import the phi-psi analysis module:

```
from dynamispectra.PhiPsi import phipsi_analysis
```

Example input files:

```
simulation1_files = [
    r'E:\Conrado\PNU\Dynamics\rama_rep1.xvg',
    r'E:\Conrado\PNU\Dynamics\rama_rep2.xvg',
    r'E:\Conrado\PNU\Dynamics\rama_rep3.xvg'
]

output_folder = r'C:\Users\Conrado\Documents\Test'

phipsi_config = {
    # Colormap for KDE heatmaps
    'cmap': 'jet',
    # KDE grid resolution
```

(continues on next page)

(continued from previous page)

```
    'grid_size': 100,
    # Heatmap opacity
    'alpha': 1,
    # Size of each subplot (width, height)
    'figsize_subplot': (6, 5),
    # Overall figure size (currently unused)
    'figsize_overall': (6, 6),
    # Font size for axis labels and colorbar
    'label_fontsize': 12,
    # Custom names for each simulation group
    'group_names': ['Control Group']
}

phipsi_analysis(
    output_folder,
    simulation1_files,
    phipsi_config=phipsi_config,
    residue_name="ALA-30"
)
```

The resulting KDE plots display favored φ and ψ angle regions per group, facilitating comparison of backbone conformational sampling.

To analyze all residues without filtering, omit the *residue_name* parameter or set it to *None*:

```
phipsi_analysis(
    output_folder,
    simulation1_files,
    phipsi_config=phipsi_config
)
```

4.12 Rotamers (Dihedral χ1 and χ2 Angles)

DynamiSpectra supports detailed analysis of side-chain rotamer states through the dihedral angles χ1 and χ2 extracted from .xvg files generated by GROMACS. This analysis helps characterize conformational changes and rotamer preferences during molecular dynamics simulations. Circular mean statistics are used to average χ1 and χ2 angles across replicates, properly accounting for their periodic nature. This module generates three types of plots to visualize rotamer distributions.

- **KDE**: Kernel Density Estimation (2D KDE heatmap) showing the probability density of χ1 and χ2 angles combined.
- **Dotplot**: Scatter plot filtered by the selected time window.
- **Histogram (Hist)**: Distribution plots of χ1 and χ2 angles separately, showing frequency density.

You can extract χ1 and χ2 dihedral angles with GROMACS using commands like:

```
gmx chi -s Simulation.tpr -f Simulation.xtc -n index.ndx -o chi1.xvg -type dihedral
gmx chi -s Simulation.tpr -f Simulation.xtc -n index.ndx -o chi2.xvg -type dihedral
```

These visualizations provide a comprehensive view of side-chain conformations during the simulation.

Note: The atom groups defining the χ1 and χ2 dihedral angles must be correctly specified in the GROMACS index file (*.ndx*) prior to angle calculation.

Code Execution

Import the rotamer analysis function from *DynamiSpectra*:

```
from dynamispectra.Rotamers import dihedral_kde_and_dotplot
```

Example input files for χ1 and χ2 angles:

```
chi1_files = [
    r'E:\Conrado\PNU\Dynamics\rotamers_chi1_chi2\chi1_time_Rep1.xvg',
    r'E:\Conrado\PNU\Dynamics\rotamers_chi1_chi2\chi1_time_Rep2.xvg',
    r'E:\Conrado\PNU\Dynamics\rotamers_chi1_chi2\chi1_time_Rep3.xvg'
]

chi2_files = [
    r'E:\Conrado\PNU\Dynamics\rotamers_chi1_chi2\chi2_time_Rep1.xvg',
    r'E:\Conrado\PNU\Dynamics\rotamers_chi1_chi2\chi2_time_Rep2.xvg',
    r'E:\Conrado\PNU\Dynamics\rotamers_chi1_chi2\chi2_time_Rep3.xvg'
]

output_folder = r'C:\Users\Conrado\Documents\Test'
```

Specify the time window (in picoseconds) to filter the angle data analyzed:

```
time_window = (35000, 40000)  # Analyze frames between a time chosen
```

Run the analysis and generate KDE maps, dotplots, and histograms with customized titles, labels, colors, and output filename:

```
dihedral_kde_and_dotplot(
    output_folder,
    chi1_files,
    chi2_files,
    config={
        'kde_title': 'Rotamers of Leu34 at 35-40 ns',
        'dot_title': 'Rotamers of Leu34 at 35-40 ns',
        'hist_title': 'Rotamer Distributions of Leu34 at 35-40 ns',
        # X-axis label for histograms
        'hist_xlabel': 'Angles (°)',
        # Y-axis label for histograms
        'hist_ylabel': 'Density',
        # Histogram legend labels
        'hist_legend_labels': [r'$\mathit{\chi_1}$', r'$\mathit{\chi_2}$'],
        # Output filename
        'save_name': 'rotamers_plot.png',
        # Colormap for KDE heatmaps
        'cmap': 'Oranges',
        # Dot color in dotplot
        'dot_color': 'brown',
        # Label for KDE colorbar
        'colorbar_label': 'Estimated Density',
        # Histogram color for χ1
        'chi1_color': 'brown',
        # Histogram color for χ2
        'chi2_color': 'orange'
    },
    time_window=time_window
)
```

The output will include combined KDE, dotplot, and histogram figures illustrating the rotamer distributions for the selected residue and time window.

To change the analyzed residue or time window, modify the input files and *time_window* parameter accordingly.

4.13 Ligand Density

DynamiSpectra enables visualization of ligand density maps using *.xpm* files generated by GROMACS. These maps represent the spatial occupancy of ligand atoms around the target molecule during the simulation, helping identify preferred binding regions or solvent-exposed areas.

The *.xpm* file can be generated with a GROMACS command such as:

```
gmx densmap -f Simulation.xtc -s Simulation.tpr -n index.ndx -o ligand_density.xpm
```

This analysis provides a 2D heatmap of ligand occupancy, with colors representing the frequency of ligand presence in specific spatial bins.

Code Execution

Import the ligand density analysis module:

```
from dynamispectra.ligand_density import ligand_density_analysis
```

Example usage for one *.xpm* file and specifying output plot parameters:

```
xpm_file_path = r'C:\Users\Conrado\Desktop\DynamiSpectra\datas\Ligand_Density\
↪ligand_density.xpm'

output_path = r'C:\Users\Conrado\Documents\Test'

ligand_density_analysis(
    # Input .xpm file path
    xpm_file_path,
    # Output path prefix (no extension added)
    output_path=output_path,
    # Show plot interactively
    plot=True,
    # Colormap for heatmap visualization
    cmap='inferno',
    # X-axis label
    xlabel='Z-axis (nm)',
    # Y-axis label
    ylabel='X-axis (nm)',
    # Plot title
    title='Ligand Density around Target',
    # Colorbar label
    colorbar_label='Occupancy',
     # Figure size in inches (width, height)
    figsize=(7, 6),
    # Font size for axis labels and colorbar
    label_fontsize=12
)
```

The resulting plots are saved in *.png* and *.tiff* formats at the specified output location.

To use default plotting parameters, call the function with just the *.xpm* file and output path:

```
ligand_density_analysis(xpm_file_path, output_path)
```

4.14 Ligand Dihedral Angle Analysis

DynamiSpectra provides analysis and visualization of ligand dihedral angles over time, allowing insight into conformational changes and flexibility of ligand torsions during molecular dynamics simulations.

Input data consist of *.xvg* files containing dihedral angle values measured at each simulation frame for multiple replicas across different simulation groups.

```
gmx gangle -f Simulation.xtc -n Simulation.ndx -g1 dihedral -group1 diedro_1 -oall␣
↪diedro_ligand.xvg
```

Note: To extract ligand dihedral angles using GROMACS, the specific atoms defining each dihedral must be predefined in the index (*.ndx*) file.

Code Execution

Import the ligand angle analysis function:

```
from dynamispectra.LigandAngle import angle_ligand_analysis
```

Example with three simulation groups, each containing three replicate *.xvg* files:

```
simulation1_files = [
    r'E:\Project\MD\LigandDihedral\ligand_dihedral_Rep1_angle1.xvg',
    r'E:\Project\MD\LigandDihedral\ligand_dihedral_Rep2_angle1.xvg',
    r'E:\Project\MD\LigandDihedral\ligand_dihedral_Rep3_angle1.xvg',
]

simulation2_files = [
    r'E:\Project\MD\LigandDihedral\ligand_dihedral_Rep1_angle1.xvg',
    r'E:\Project\MD\LigandDihedral\ligand_dihedral_Rep2_angle1.xvg',
    r'E:\Project\MD\LigandDihedral\ligand_dihedral_Rep3_angle1.xvg',
]

simulation3_files = [
    r'E:\Project\MD\LigandDihedral\ligand_dihedral_Rep1_angle1.xvg',
    r'E:\Project\MD\LigandDihedral\ligand_dihedral_Rep2_angle1.xvg',
    r'E:\Project\MD\LigandDihedral\ligand_dihedral_Rep3_angle1.xvg',
]


output_folder = r'C:\Users\Bioinformatics\Documents\LigandAngleResults'

time_config = {
# Legend labels
'labels': ['Simulation 1', 'Simulation 2', 'Simulation 3'],
# Line colors
'colors': ['#333333', '#6A9EDA', '#49C547'],
# Shaded area transparency (±SD)
'alpha': 0.2,
# Figure size (width, height)
'figsize': (9, 6),
# X-axis label
'xlabel': 'Time (ns)',
# Y-axis label
'ylabel': 'Dihedral angle (°)',
# Font size for axis labels
'label_fontsize': 12,
# Smoothing window (optional)
'smooth_window': 20,
# Y-axis range
'ylim': (110, 127)
}

```

(continues on next page)

(continued from previous page)

```
density_config = {
# Legend labels
'labels': ['Simulation 1', 'Simulation 2', 'Simulation 3'],
# Fill colors
'colors': ['#333333', '#6A9EDA', '#49C547'],
# Transparency for KDE
'alpha': 0.6,
# Figure size
'figsize': (6, 6),
# X-axis label
'xlabel': 'Dihedral angle (°)',
# Y-axis label
'ylabel': 'Density',
# Font size for axis labels
'label_fontsize': 12
}

kde2d_config = {
# Legend labels
'labels': ['Simulation 1', 'Simulation 2', 'Simulation 3'],
# Marker/line colors
'colors': ['#333333', '#6A9EDA', '#49C547'],
# Figure size
'figsize': (8, 6),
# X-axis label
'xlabel': 'Time (ns)',
# Y-axis label
'ylabel': 'Dihedral angle (°)',
# Font size for axis labels
'label_fontsize': 12,
# Colormap for 2D KDE heatmap
'cmap': 'Oranges'
}


angle_ligand_analysis(
    output_folder,
    simulation1_files,
    simulation2_files,
    simulation3_files,
    time_config=time_config,
    density_config=density_config,
    kde2d_config=kde2d_config
)
```

This will produce plots of dihedral angles over time with mean ± standard deviation shading, KDE density distributions, and 2D KDE heatmaps showing angle vs. simulation time, all saved to the specified output folder.

To run the analysis with a single group or a single file, just provide one list:

```
angle_ligand_analysis(output_folder, simulation1_files)
```

If no configuration dictionaries are provided, default plot settings will be applied. You can tailor the plot appearance for presentations or publications by adjusting the dictionary values.

4.15 Principal Component Analysis (PCA)

DynamiSpectra provides tools to visualize and interpret the results of principal component analysis (PCA) applied to molecular dynamics trajectories. This analysis allows the identification of dominant motions and conformational changes by projecting atomic displacements onto principal components.

The input files consist of:

- A *.xvg* file containing the projection of atomic displacements onto PC1 and PC2 (typically generated using *gmx covar* and *gmx anaeig*)
- A *.xvg* file containing the eigenvalues for each principal component (typically *eigenval.xvg* from *gmx anaeig*)

Code Execution

Import the PCA analysis function:

```
from dynamispectra.PCA import pca_analysis
```

Define input files, output folder and run the analysis:

```
pca_file_path = r'C:\Users\Conrado\Desktop\DynamiSpectra\data\PCA\pca_projection.
↪xvg'
eigenval_path = r'C:\Users\Conrado\Desktop\DynamiSpectra\data\PCA\eigenval.xvg'
output_folder = r'C:\Users\Conrado\Documents\Test'

pca_analysis(
# Path to PCA projection file
pca_file_path,
# Path to eigenvalue file
eigenval_path,
# Output folder to save the plot
output_folder,
# Title of the plot
title='',
# Size of the figure (width, height)
figsize=(8, 7),
# Colormap used for time coloring
cmap='inferno',
# Size of the scatter points
point_size=50,
# Font size for X and Y axis labels
axis_label_fontsize=15,
# Font size for the title
title_fontsize=16,
# Font size for the colorbar label
colorbar_label_fontsize=13,
# Font size for the colorbar ticks
colorbar_tick_fontsize=10
)
```

This will generate a PCA scatter plot where each point represents a frame projected in the PC1–PC2 space, colored according to simulation time. The fraction of variance explained by PC1 and PC2 is automatically extracted from the eigenvalue file and included in the axis labels. You can modify the colormap, point size, and transparency as needed for presentation or publication purposes.

To use default settings, omit the optional keyword arguments.

4.16 Secondary Structure Probability

DynamiSpectra provides tools for the analysis and comparison of secondary structure content across different simulation groups. The analysis is based on *.dat* files containing the fraction of time each residue spent in different secondary structure elements (e.g., helix, sheet, coil) throughout the trajectory.

These *.dat* files are typically generated by external tools such as **dssp** in GROMACS or *gmx dssp*, followed by post-processing to calculate structure probabilities per residue.

Code Execution

Import the secondary structure analysis function:

```
from dynamispectra.SecondaryStructure import ss_analysis
```

Define input files for each simulation group (each group consists of replicate *.dat* files), and run the analysis:

```
simulation1_files = [
    r'C:\Users\Conrado\Desktop\DynamiSpectra\simulation1_rep1.dat',
    r'C:\Users\Conrado\Desktop\DynamiSpectra\simulation1_rep2.dat',
    r'C:\Users\Conrado\Desktop\DynamiSpectra\simulation1_rep3.dat'
]

simulation2_files = [
    r'C:\Users\Conrado\Desktop\DynamiSpectra\simulation2_rep1.dat',
    r'C:\Users\Conrado\Desktop\DynamiSpectra\simulation2_rep2.dat',
    r'C:\Users\Conrado\Desktop\DynamiSpectra\simulation2_rep3.dat'
]

simulation3_files = [
    r'C:\Users\Conrado\Desktop\DynamiSpectra\simulation3_rep1.dat',
    r'C:\Users\Conrado\Desktop\DynamiSpectra\simulation3_rep2.dat',
    r'C:\Users\Conrado\Desktop\DynamiSpectra\simulation3_rep3.dat'
]

output_folder = r'C:\Users\Conrado\Documents\Test'

plot_config = {
    # Names of the groups
    'labels': ['Simulation 1', 'Simulation 2', 'Simulation 3'],
    # Colors for each group
    'colors': ['#333333', '#6A9EDA', '#49C547'],
    # Transparency of the lines or shaded area
    'alpha': 0.6,
    # Font size for axis labels
    'axis_label_size': 12,
    # Label for the Y-axis
    'y_axis_label': 'Secondary Structure Probability (%)',
    # Figure size (width, height)
    'figsize': (9, 7)
}

ss_analysis(
    output_folder,
    simulation1_files,
    simulation2_files,
    simulation3_files,
    plot_config=plot_config
)
```

This will produce a plot showing the average secondary structure probability across residues, with shaded regions representing the standard deviation among replicates for each simulation group. If no plot configuration is provided, default styles will be applied.

4.17 Secondary Structure Fractions

DynamiSpectra allows visualization of the time evolution of global secondary structure content during molecular dynamics simulations. This analysis provides insights into the overall structural dynamics by quantifying the fraction of residues adopting each secondary structure type (helix, sheet, coil) per frame.

The input *.dat* file must contain secondary structure assignments per frame, usually obtained using *gmx dssp* followed by conversion to per-frame structural fractions.

Code Execution

Import the analysis function:

```
from dynamispectra.FractionSS import fractions_ss_analysis
```

Specify the input file, output folder and optional plot configuration:

```
file_path = r'C:\Users\Conrado\Desktop\DynamiSpectra\datas\SecondaryStructure\
↪simulation1.dat'
output_folder = r'C:\Users\Conrado\Documents\Test'

plot_config = {
    # Plot title
    'title': 'Secondary Structure Fractions Over Time',
    # Label for x-axis
    'xlabel': 'Simulation Frames',
    # Label for y-axis
    'ylabel': 'Residue Fraction',
    # Size of the figure
    'figsize': (9, 6),
    # Font size for labels
    'fontsize': 12,
    # X-axis range (optional)
    'xlim': (0, 10000),
    # Y-axis range (optional)
    'ylim': (0, 0.85)
}
```

Run the analysis and export the plot and Excel summary:

```
fractions_ss_analysis(file_path, output_folder, plot_config=plot_config)
```

This function will produce:

- A time series plot showing the evolution of helix, sheet, and coil content over time.
- An Excel file (*SecondaryStructure_Fractions.xlsx*) containing both raw and average values of each structure type.

If no configuration is provided, the plot will be generated using default settings.

4.18 Pressure Analysis

DynamiSpectra enables visualization of system pressure throughout molecular dynamics simulations. Pressure analysis helps assess system equilibration, barostat performance, and simulation stability over time. This module accepts *.xvg* output files generated by GROMACS using the built-in pressure calculation options during simulation.

To generate the required *.xvg* file for temperature using GROMACS, run:

```
gmx energy -f Simulation.edr -o pressure.xvg
```

Input data consists of *.xvg* files output by GROMACS temperature calculations.

Code Execution

Import the pressure analysis function:

```
from dynamispectra.Pressure import pressure_analysis
```

Define the input files for each simulation group:

```
simulation1_files = [
    r'E:\Conrado\PNU\Dynamics\pressure_sim1_rep1.xvg',
    r'E:\Conrado\PNU\Dynamics\pressure_sim1_rep2.xvg',
    r'E:\Conrado\PNU\Dynamics\pressure_sim1_rep3.xvg',
]

simulation2_files = [
    r'E:\Conrado\PNU\Dynamics\pressure_sim2_rep1.xvg',
    r'E:\Conrado\PNU\Dynamics\pressure_sim2_rep2.xvg',
    r'E:\Conrado\PNU\Dynamics\pressure_sim2_rep3.xvg',
]

simulation3_files = [
    r'E:\Conrado\PNU\Dynamics\pressure_sim3_rep1.xvg',
    r'E:\Conrado\PNU\Dynamics\pressure_sim3_rep2.xvg',
    r'E:\Conrado\PNU\Dynamics\pressure_sim3_rep3.xvg',
]
```

Specify the output folder and run the analysis:

```
output_folder = r'C:\Users\Conrado\Documents\Test'

pressure_config = {
    # Legend labels
    'labels': ['Simulation 1', 'Simulation 2', 'Simulation 3'],
    # Line colors for each group
    'colors': ['#333333', '#6A9EDA', '#49C547'],
    # Figure size (width, height)
    'figsize': (10, 6),
    # Std deviation fill transparency
    'alpha': 0.3,
    # X-axis label
    'xlabel': 'Time (ns)',
    # Y-axis label
    'ylabel': 'Pressure (bar)',
    # Font size for axis and legend
    'label_fontsize': 14,
    # Y-axis range
    'ylim': (-900, 900)
}

density_config = {
    # Legend labels
    'labels': ['Simulation 1', 'Simulation 2', 'Simulation 3'],
    # Fill colors for KDE plots
```

(continues on next page)

(continued from previous page)

```
    'colors': ['#333333', '#6A9EDA', '#49C547'],
    # Figure size (width, height)
    'figsize': (6, 6),
    # Fill transparency
    'alpha': 0.5,
    # X-axis label
    'xlabel': 'Pressure (bar)',
    # Y-axis label
    'ylabel': 'Density',
    # Font size for labels
    'label_fontsize': 14,
    # Minimum x-value for KDE plot
    'x_min': -900,
    # Maximum x-value for KDE plot
    'x_max': 900
}

pressure_analysis(
    output_folder,
    simulation1_files,
    simulation2_files,
    simulation3_files,
    pressure_config=pressure_config,
    density_config=density_config
)
```

This will generate a time series plot showing the pressure profile with mean ± standard deviation for each group, and a KDE plot of pressure distributions.

4.19 Temperature Analysis

DynamiSpectra allows analysis and visualization of system temperature during molecular dynamics simulations. Temperature profiles help monitor system equilibration and thermal stability.

To generate the required *.xvg* file for temperature using GROMACS, run:

```
gmx energy -f Simulation.edr -o temperature.xvg
```

Input data consists of *.xvg* files output by GROMACS temperature calculations.

Code Execution

Import the temperature analysis function:

```
from dynamispectra.Temperature import temperature_analysis
```

Define the input *.xvg* files for each simulation group (each group with multiple replicates):

```
simulation1_files = [
    r'E:\Conrado\PNU\Dinamica\temperature_rep1.xvg',
    r'E:\Conrado\PNU\Dinamica\temperature_rep2.xvg',
    r'E:\Conrado\PNU\Dinamica\temperature_rep3.xvg'
]

simulation2_files = [
    r'E:\Conrado\PNU\Dinamica\temperature_rep1.xvg',
    r'E:\Conrado\PNU\Dinamica\temperature_rep2.xvg',
```

(continues on next page)

(continued from previous page)

```
    r'E:\Conrado\PNU\Dinamica\temperature_rep3.xvg'
]

simulation3_files = [
    r'E:\Conrado\PNU\Dinamica\temperature_rep1.xvg',
    r'E:\Conrado\PNU\Dinamica\temperature_rep2.xvg',
    r'E:\Conrado\PNU\Dinamica\temperature_rep3.xvg'
]
```

Specify the output folder to save the plots and run the analysis:

```
output_folder = r'C:\Users\Conrado\Documents\Test'

temp_config = {
    # Legend labels
    'labels': ['Simulation 1', 'Simulation 2', 'Simulation 3'],
    # Line colors for each group
    'colors': ['#6A9EDA', '#e04f4f', "#bca140"],
    # Transparency for std deviation shading
    'alpha': 0.3,
    # Figure size (width, height)
    'figsize': (9, 6),
    # X-axis label
    'xlabel': 'Time (ns)',
    # Y-axis label
    'ylabel': 'Temperature (K)',
    # Font size for axis labels
    'label_fontsize': 12
}

density_config = {
    # Legend labels
    'labels': ['Simulation 1', 'Simulation 2', 'Simulation 3'],
    # Fill colors for KDE plots
    'colors': ['#6A9EDA', '#e04f4f', '#bca140'],
    # Transparency for KDE fill
    'alpha': 0.6,
    # Figure size (width, height)
    'figsize': (6, 6),
    # X-axis label
    'xlabel': 'Temperature (K)',
    # Y-axis label
    'ylabel': 'Density',
    # Font size for axis labels
    'label_fontsize': 12
}

temperature_analysis(
    output_folder,
    simulation1_files,
    simulation2_files,
    simulation3_files,
    temp_config=temp_config,
    density_config=density_config
)
```

This will generate time series plots showing temperature fluctuations with mean ± standard deviation and KDE plots

representing the temperature distribution for each simulation group.

To analyze a single simulation group, provide a single list of files. If the configuration dictionaries are omitted, default plotting parameters will be applied.

4.20 Density Analysis

DynamiSpectra allows analysis and visualization of system density throughout molecular dynamics simulations. This provides insight into system stability, phase transitions, and equilibration.

This module processes *.xvg* files typically generated by GROMACS during simulation or via energy analysis tools.

To obtain density data from GROMACS, you can use the following command after your simulation:

```
gmx energy -f Simulation.edr -o density.xvg
```

Select the density property when prompted.

Code Execution

Import the density analysis function:

```
from dynamispectra.Density import density_analysis
```

Define the input files for each simulation group:

```
simulation1_files = [
    r'E:\Conrado\PNU\Dinamica\simulation1\density_rep1.xvg',
    r'E:\Conrado\PNU\Dinamica\simulation1\density_rep2.xvg',
    r'E:\Conrado\PNU\Dinamica\simulation1\density_rep3.xvg',
]

simulation2_files = [
    r'E:\Conrado\PNU\Dinamica\simulation2\density_rep1.xvg',
    r'E:\Conrado\PNU\Dinamica\simulation2\density_rep2.xvg',
    r'E:\Conrado\PNU\Dinamica\simulation2\density_rep3.xvg',
]

simulation3_files = [
    r'E:\Conrado\PNU\Dinamica\simulation3\density_rep1.xvg',
    r'E:\Conrado\PNU\Dinamica\simulation3\density_rep2.xvg',
    r'E:\Conrado\PNU\Dinamica\simulation3\density_rep3.xvg',
]
```

Specify the output folder and run the analysis:

```
output_folder = r'C:\Users\Conrado\Documents\Test'

density_config = {
    # Legend labels for each simulation group
    'labels': ['Simulation 1', 'Simulation 2', 'Simulation 3'],
    # Colors for each line and shaded area
    'colors': ['#333333', '#6A9EDA', '#49C547'],
    # Figure size (width, height)
    'figsize': (10, 6),
    # Transparency of shaded std deviation area
    'alpha': 0.3,
    # X-axis label
    'xlabel': 'Time (ns)',
    # Y-axis label
```

(continues on next page)

(continued from previous page)

```
    'ylabel': 'Density (kg/m³)',
    # Font size for axis labels
    'label_fontsize': 14,
    # Y-axis limits for better visualization
    'ylim': (950, 1010)
}

distribution_config = {
    # Legend labels for KDE plots
    'labels': ['Simulation 1', 'Simulation 2', 'Simulation 3'],
    # Colors for KDE fills
    'colors': ['#333333', '#6A9EDA', '#49C547'],
    # Figure size
    'figsize': (6, 6),
    # Transparency for KDE fills
    'alpha': 0.5,
    # X-axis label
    'xlabel': 'Density (kg/m³)',
    # Y-axis label
    'ylabel': 'Density',
    # Font size for axis labels
    'label_fontsize': 14,
    # Minimum x value for KDE plot range
    'x_min': 950,
    # Maximum x value for KDE plot range
    'x_max': 1010
}

density_analysis(
    output_folder,
    simulation1_files,
    simulation2_files,
    simulation3_files,
    density_config=density_config,
    distribution_config=distribution_config
)
```

This will generate plots of density over time with mean ± standard deviation shading, plus KDE density distribution plots, all saved to the specified output folder.

5 RMSD

5.1 Overview

DynamiSpectra enables comprehensive analysis of Root Mean Square Deviation (RMSD) from molecular dynamics simulations using .xvg files. The tool supports the processing of multiple replicates per simulation group, allowing the calculation of average and standard deviation to capture structural deviations over time. However, it can also be applied to a single replicate, making it suitable for both exploratory analyses and more robust comparative studies involving multiple trajectories.

The generated plots provide time series visualizations with shaded standard deviations and kernel density distributions of RMSD values. This allows users to assess the dynamic stability of systems across simulations and compare structural drift between conditions.

Command line in GROMACS to generate .xvg files for the analysis:

```
gmx rms -s Simulation.tpr -f Simulation.xtc -n index.ndx -tu ns -o Simulation.xvg
```

```
def rmsd_analysis(output_folder, *simulation_file_groups, rmsd_config=None,␣
↪density_config=None)
```

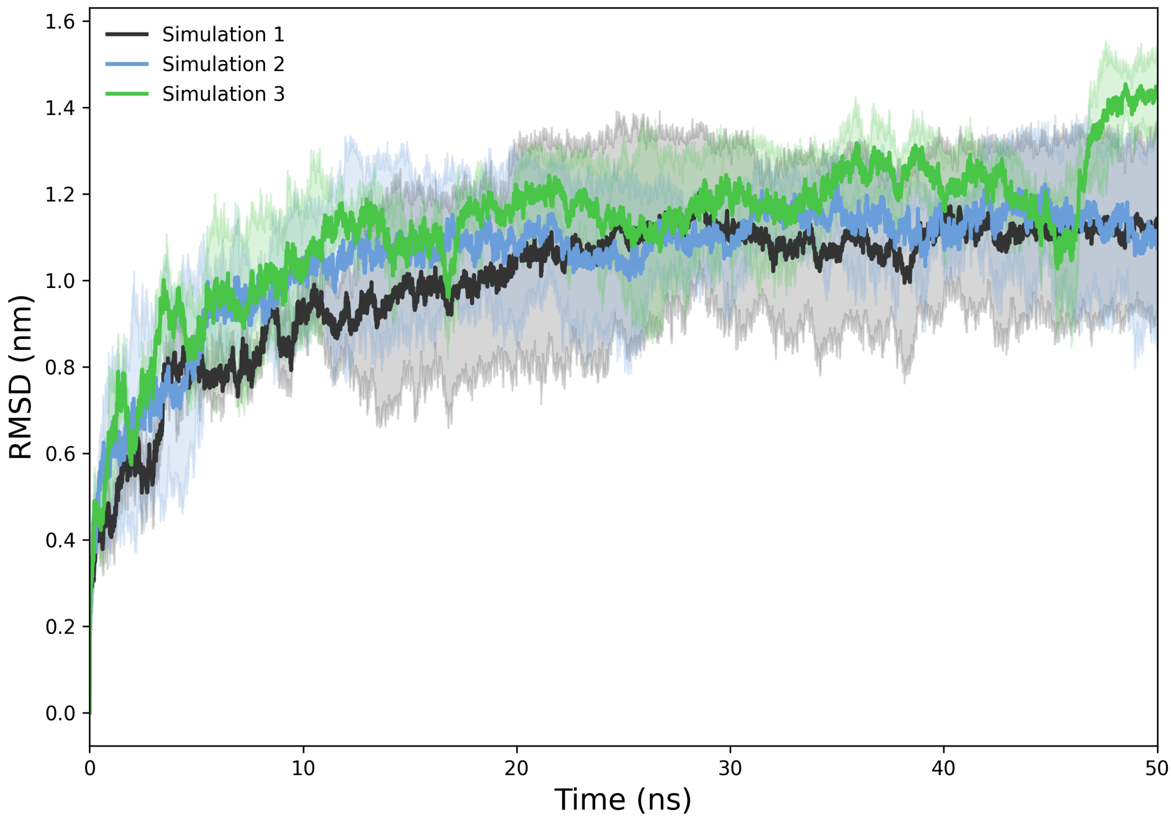

Interpretation guidence: This plot illustrates the temporal evolution of structural deviation relative to the reference conformation during the simulation. A stable RMSD plateau indicates that the system has equilibrated and maintains a consistent structure. Gradual increases or large fluctuations suggest ongoing conformational changes, instability, or unfolding events. Assessing the RMSD trend helps identify when the system reaches equilibrium and whether structural deviations remain within expected biological variability.

```
def plot_density_all(results, output_folder, config)
```

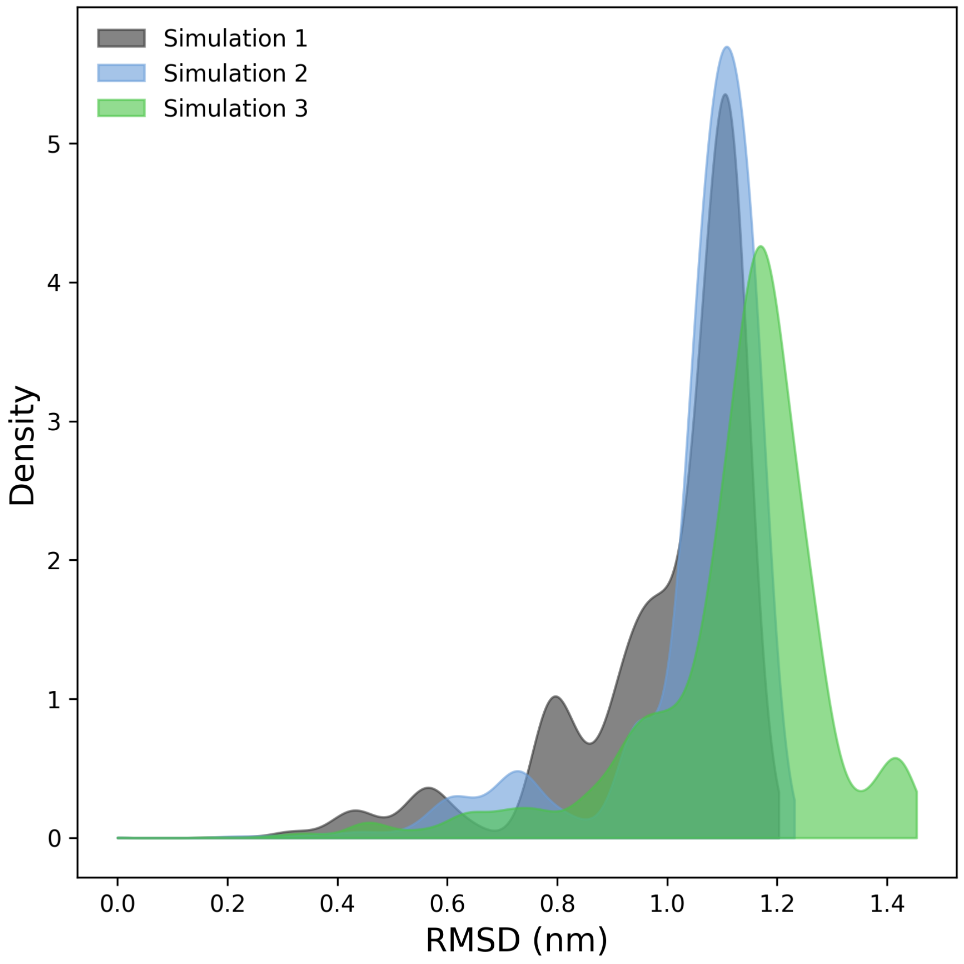

Interpretation guidance: This plot shows how frequently different RMSD values occur during the simulation. Sharp peaks indicate stable conformations, while broader or multiple peaks reveal structural flexibility or transitions between states.

5.2 Complete code

```
import numpy as np
import matplotlib.pyplot as plt
from scipy.stats import gaussian_kde
import os
```

```
def read_rmsd(file):
```

```
"""
Reads RMSD data from a .xvg file.
Skips header/comment lines and extracts time and RMSD values.
"""
try:
    print(f"Reading file: {file}")
    times, rmsd = [], []
    with open(file, 'r') as f:
        for line in f:
            if line.startswith(('#', '@', ';')) or line.strip() == '':
                continue
```

(continues on next page)

(continued from previous page)

```
            try:
                values = line.split()
                if len(values) >= 2:
                    time, rmsd_val = map(float, values[:2])
                    times.append(time)
                    rmsd.append(rmsd_val)
            except ValueError:
                print(f"Error processing line: {line.strip()}")
                continue
    if len(times) == 0 or len(rmsd) == 0:
        raise ValueError(f"File {file} does not contain valid data.")
    return np.array(times), np.array(rmsd)
except Exception as e:
    print(f"Error reading file {file}: {e}")
    return None, None
```

```
def check_simulation_times(*time_arrays):
```

```
"""
Ensure all replicate time arrays in a group are aligned.
"""
for i in range(1, len(time_arrays)):
    if not np.allclose(time_arrays[0], time_arrays[i]):
        raise ValueError(f"Simulation times do not match between replicate 1 and␣
↪replicate {i+1}")
```

```
def plot_rmsd_all(results, output_folder, config):
```

```
"""
Plot RMSD time series with mean ± std for all simulation groups.
"""
labels = config.get('labels', [f'Simulation {i+1}' for i in range(len(results))])
colors = config.get('colors', None)
alpha = config.get('alpha', 0.2)
figsize = config.get('figsize', (8, 6))
xlabel = config.get('xlabel', 'Time (ps)')
ylabel = config.get('ylabel', 'RMSD (nm)')
label_fontsize = config.get('label_fontsize', 12)
xlim = config.get('xlim', None)
ylim = config.get('ylim', None)

plt.figure(figsize=figsize)

for i, (time, mean, std) in enumerate(results):
    color = colors[i] if colors and i < len(colors) else None
    plt.plot(time, mean, label=labels[i], color=color, linewidth=2)
    plt.fill_between(time, mean - std, mean + std, color=color, alpha=alpha)

plt.xlabel(xlabel, fontsize=label_fontsize)
plt.ylabel(ylabel, fontsize=label_fontsize)
plt.legend(frameon=False, loc='best', fontsize=10)
plt.tick_params(axis='both', which='major', labelsize=10)

if xlim:
    plt.xlim(xlim)
```

(continues on next page)

(continued from previous page)

```
else:
    max_time = max([np.max(time) for time, _, _ in results])
    plt.xlim(0, max_time)

if ylim:
    plt.ylim(ylim)

plt.tight_layout()
os.makedirs(output_folder, exist_ok=True)
plt.savefig(os.path.join(output_folder, 'rmsd_plot.tiff'), dpi=300)
plt.savefig(os.path.join(output_folder, 'rmsd_plot.png'), dpi=300)
plt.show()
```

```
def plot_density_all(results, output_folder, config):
```

```
"""
Plot KDE density of RMSD values for all simulation groups.
"""
labels = config.get('labels', [f'Simulation {i+1}' for i in range(len(results))])
colors = config.get('colors', None)
alpha = config.get('alpha', 0.5)
figsize = config.get('figsize', (6, 6))
xlabel = config.get('xlabel', 'RMSD (nm)')
ylabel = config.get('ylabel', 'Density')
label_fontsize = config.get('label_fontsize', 12)
xlim = config.get('xlim', None)
ylim = config.get('ylim', None)

plt.figure(figsize=figsize)

for i, (_, mean, _) in enumerate(results):
    color = colors[i] if colors and i < len(colors) else None
    kde = gaussian_kde(mean)
    x_min = 0
    x_max = max(mean) if max(mean) > 0 else 1
    x_vals = np.linspace(x_min, x_max, 1000)
    plt.fill_between(x_vals, kde(x_vals), color=color, alpha=alpha,␣
↪label=labels[i])

plt.xlabel(xlabel, fontsize=label_fontsize)
plt.ylabel(ylabel, fontsize=label_fontsize)
plt.legend(frameon=False, loc='best', fontsize=10)
plt.tick_params(axis='both', which='major', labelsize=10)

if xlim:
    plt.xlim(xlim)
if ylim:
    plt.ylim(ylim)

plt.tight_layout()
os.makedirs(output_folder, exist_ok=True)
plt.savefig(os.path.join(output_folder, 'density_plot.tiff'), dpi=300)
plt.savefig(os.path.join(output_folder, 'density_plot.png'), dpi=300)
plt.show()
```

```
def rmsd_analysis(output_folder, *simulation_file_groups, rmsd_config=None,␣
↪density_config=None):
```

```
"""
Main function to process multiple simulation groups, each with multiple replicate␣
↪files.
Computes mean and std deviation for each group and generates plots.
"""
if rmsd_config is None:
    rmsd_config = {}
if density_config is None:
    density_config = {}

def process_group(file_paths):
    times = []
    rmsd_vals = []
    for file in file_paths:
        time, rmsd = read_rmsd(file)
        if time is None or rmsd is None:
            raise ValueError(f"Error reading file: {file}. Please verify the file␣
↪content.")
        times.append(time)
        rmsd_vals.append(rmsd)
    check_simulation_times(*times)
    mean_rmsd = np.mean(rmsd_vals, axis=0)
    std_rmsd = np.std(rmsd_vals, axis=0)
    return times[0], mean_rmsd, std_rmsd

results = []
for group in simulation_file_groups:
    if group:
        result = process_group(group)
        results.append(result)

if len(results) == 0:
    raise ValueError("You must provide at least one group of simulation files.")

plot_rmsd_all(results, output_folder, rmsd_config)
plot_density_all(results, output_folder, density_config)
```

6

RMSF

6.1 Overview

DynamiSpectra provides a comprehensive Root Mean Square Fluctuation (RMSF) analysis package for molecular dynamics simulations using .xvg input files. The RMSF module supports analysis of individual simulation replicas and computes averages and standard deviations across multiple replicates. The graphical outputs show the average RMSF with a shaded area representing the standard deviation, facilitating visualization of flexibility patterns.

Command line in GROMACS to generate .xvg files for the analysis:

```
gmx rmsf -f Simulation.xtc -s Simulation.tpr -o Simulation.xvg -res
```

```
def rmsf_analysis(output_folder, *simulation_files_groups, rmsf_config=None,
↪density_config=None)
```

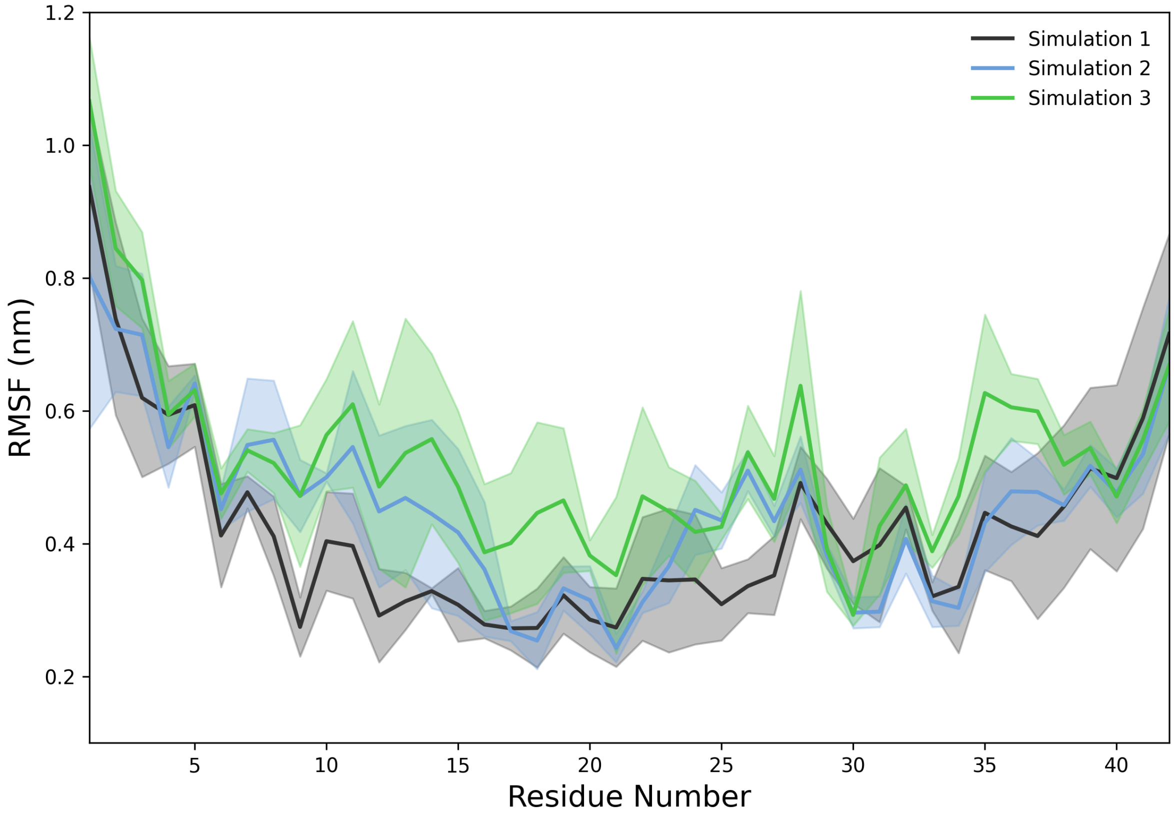

Interpretation guidance: This graph shows RMSF values across residues, where higher peaks indicate regions of greater flexibility or mobility, such as loops or termini. Lower values correspond to more rigid regions like helices or sheets. Observing these patterns helps identify functionally important flexible segments and assess the dynamic behavior of the system. Comparing RMSF profiles with references helps determine if observed fluctuations are expected or significant.

```
def plot_density(results, output_folder, config={})
```

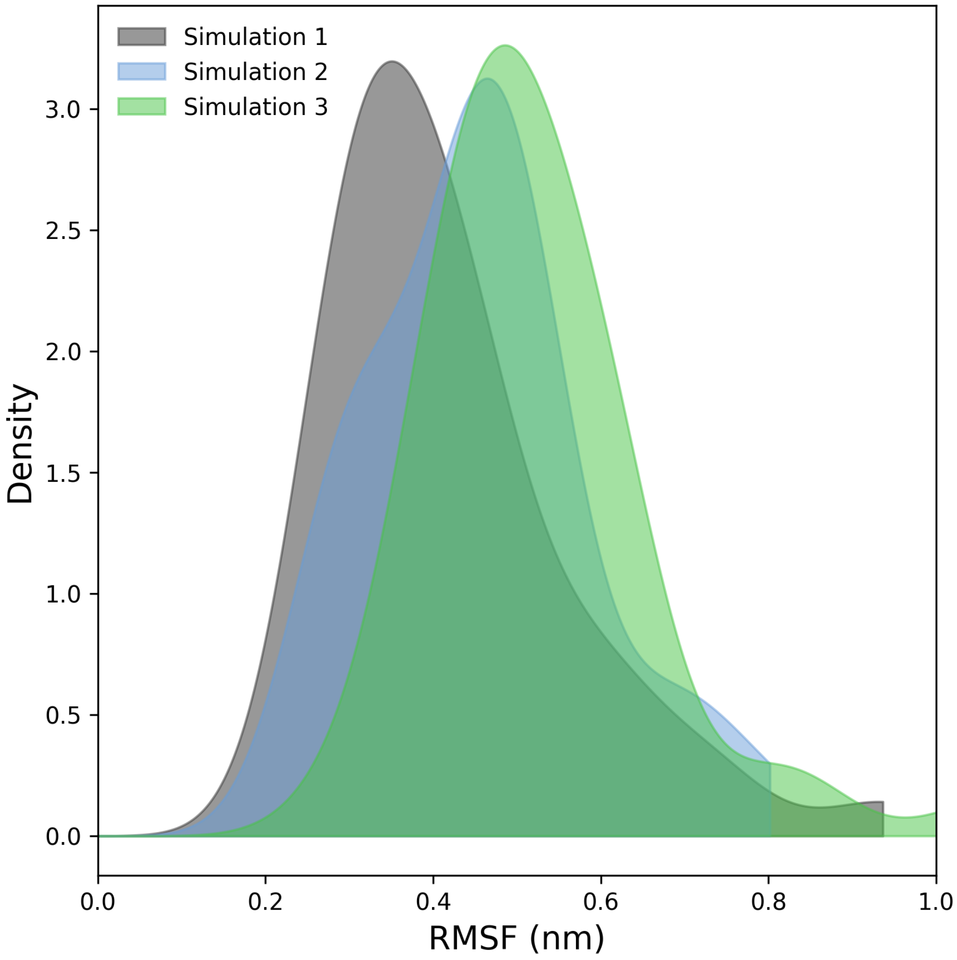

Interpretation guidance: This graph shows RMSF values across residues, where higher peaks indicate regions of greater flexibility or mobility, such as loops or termini. Lower values correspond to more rigid regions like helices or sheets. Observing these patterns helps identify functionally important flexible segments and assess the dynamic behavior of the system. Comparing RMSF profiles with references helps determine if observed fluctuations are expected or significant.

6.2 Complete code

```
import numpy as np
import matplotlib.pyplot as plt
from scipy.stats import gaussian_kde
import os
```

```
def read_rmsf(file):
```

```
try:
    print(f"Reading file: {file}")
    positions, rmsf_vals = [], []
    with open(file, 'r') as f:
        for line in f:
            if line.startswith(('#', '@', ';')) or line.strip() == '':
                continue
            try:
                values = line.split()
                if len(values) >= 2:
                    pos, rmsf = map(float, values[:2])
                    positions.append(pos)
                    rmsf_vals.append(rmsf)
            except ValueError:
                print(f"Error processing line: {line.strip()}")
                continue
    if len(positions) == 0 or len(rmsf_vals) == 0:
        raise ValueError(f"File {file} does not contain valid data.")
    return np.array(positions), np.array(rmsf_vals)
except Exception as e:
    print(f"Error reading file {file}: {e}")
    return None, None
```

```
def check_simulation_positions(*position_arrays):
```

```
for i in range(1, len(position_arrays)):
    if not np.allclose(position_arrays[0], position_arrays[i]):
        raise ValueError(f"Residue positions do not match between replicate 1 and␣
↪replicate {i+1}")
```

```
def plot_rmsf(results, output_folder, config={}):
```

```
labels = config.get('labels', [f'Simulation {i+1}' for i in range(len(results))])
colors = config.get('colors', ['#333333', '#6A9EDA', '#54b36a', '#e67e22', '#9b59b6
↪'])
alpha = config.get('alpha', 0.2)
figsize = config.get('figsize', (8, 6))
xlabel = config.get('xlabel', 'Residue')
ylabel = config.get('ylabel', 'RMSF (nm)')
label_fontsize = config.get('label_fontsize', 12)
xlim = config.get('xlim', None)
ylim = config.get('ylim', None)

plt.figure(figsize=figsize)

for i, (x, mean, std) in enumerate(results):
    plt.plot(x, mean, label=labels[i], color=colors[i % len(colors)], linewidth=2)
    plt.fill_between(x, mean - std, mean + std, color=colors[i % len(colors)],␣
↪alpha=alpha)

plt.xlabel(xlabel, fontsize=label_fontsize)
plt.ylabel(ylabel, fontsize=label_fontsize)
plt.legend(frameon=False, loc='upper right', fontsize=10)
plt.tick_params(axis='both', which='major', labelsize=10)

if xlim:
```

(continues on next page)

(continued from previous page)

```
    plt.xlim(xlim)
if ylim:
    plt.ylim(ylim)

plt.tight_layout()
os.makedirs(output_folder, exist_ok=True)
plt.savefig(os.path.join(output_folder, 'rmsf_plot.tiff'), format='tiff', dpi=300)
plt.savefig(os.path.join(output_folder, 'rmsf_plot.png'), format='png', dpi=300)
plt.show()
```

```
def plot_density(results, output_folder, config={}):
```

```
labels = config.get('labels', [f'Simulation {i+1}' for i in range(len(results))])
colors = config.get('colors', ['#333333', '#6A9EDA', '#54b36a', '#e67e22', '#9b59b6
↪'])
alpha = config.get('alpha', 0.5)
figsize = config.get('figsize', (6, 6))
xlabel = config.get('xlabel', 'RMSF (nm)')
ylabel = config.get('ylabel', 'Density')
label_fontsize = config.get('label_fontsize', 12)
xlim = config.get('xlim', None)
ylim = config.get('ylim', None)

plt.figure(figsize=figsize)

for i, (_, mean, _) in enumerate(results):
    kde = gaussian_kde(mean)
    x = np.linspace(0, max(mean), 1000)
    plt.fill_between(x, kde(x), color=colors[i % len(colors)], alpha=alpha,␣
↪label=labels[i])

plt.xlabel(xlabel, fontsize=label_fontsize)
plt.ylabel(ylabel, fontsize=label_fontsize)
plt.legend(frameon=False, loc='upper left', fontsize=10)
plt.tick_params(axis='both', which='major', labelsize=10)

if xlim:
    plt.xlim(xlim)
if ylim:
    plt.ylim(ylim)

plt.tight_layout()
os.makedirs(output_folder, exist_ok=True)
plt.savefig(os.path.join(output_folder, 'density_plot.tiff'), format='tiff',␣
↪dpi=300)
plt.savefig(os.path.join(output_folder, 'density_plot.png'), format='png', dpi=300)
plt.show()
```

```
def rmsf_analysis(output_folder, *simulation_files_groups, rmsf_config=None,␣
↪density_config=None):
```

```
if rmsf_config is None:
    rmsf_config = {}
if density_config is None:
    density_config = {}
```

(continues on next page)

(continued from previous page)

```
def process_group(file_paths):
    positions = []
    values = []
    for file in file_paths:
        pos, rmsf = read_rmsf(file)
        if pos is None or rmsf is None:
            raise ValueError(f"Error reading file: {file}")
        positions.append(pos)
        values.append(rmsf)
    check_simulation_positions(*positions)
    return positions[0], np.mean(values, axis=0), np.std(values, axis=0)

results = []
for group in simulation_files_groups:
    if group:
        result = process_group(group)
        results.append(result)

if not results:
    raise ValueError("At least one group of simulation files is required.")

plot_rmsf(results, output_folder, rmsf_config)
plot_density(results, output_folder, density_config)
```

7 Radius of gyration

7.1 Overview

DynamiSpectra provides a robust analytical tool for evaluating the radius of gyration (Rg) from molecular dynamics simulations using .xvg input files. This module supports the analysis of individual simulation replicas as well as the computation of average Rg values and standard deviations across replicates. Visual outputs include time series plots with shaded variability and Rg density distributions for each simulation group.

This analysis helps monitor the structural compactness and stability of macromolecules over time, offering valuable insight into folding behavior, domain organization, or global conformational changes throughout the simulation.

Command line in GROMACS to generate .xvg files for the analysis:

```
gmx gyrate -f Simulation.xtc -s Simulation.tpr -o gyrate_simulation.xvg
```

```
def rg_analysis(output_folder, *simulation_groups, rg_config=None, density_
↪config=None)
```

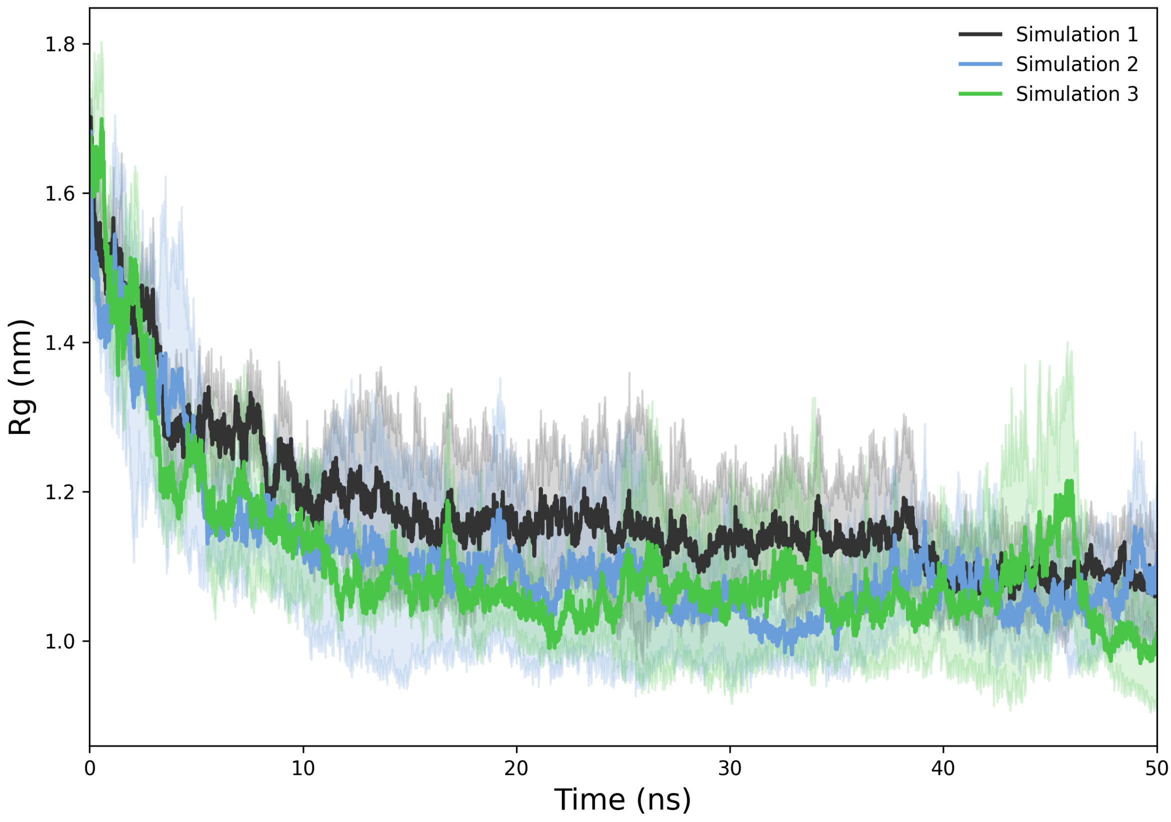

How to interpret: This plot illustrates the evolution of the radius of gyration over time. A stable or consistent Rg profile indicates that the system maintains a compact and well-defined structure throughout the simulation. In contrast, marked increases or decreases in Rg may reflect molecular expansion, unfolding, or compaction. Evaluating Rg trends in relation to expected structural behavior helps determine whether the system's compactness aligns with its functional or biological role.

```
def plot_density(results, output_folder, config)
```

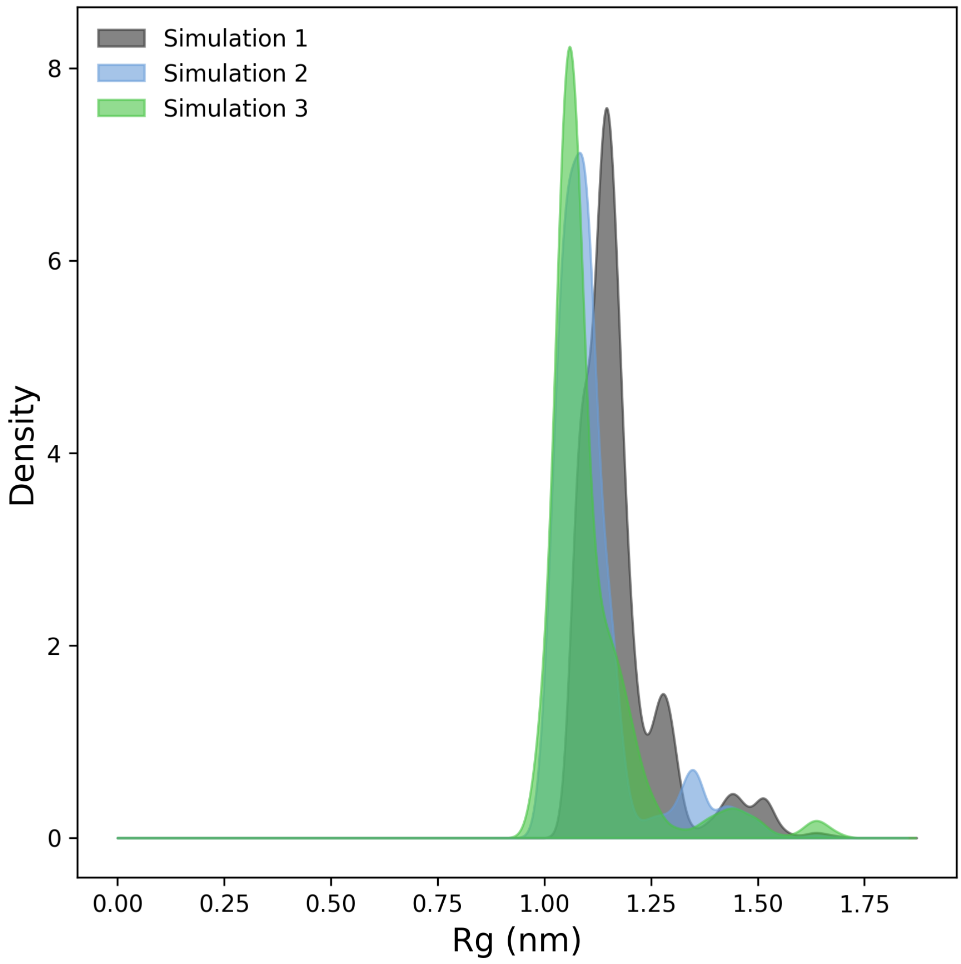

How to interpret: This plot shows the density distribution of the radius of gyration values throughout the simulation. Peaks in the distribution indicate the most frequently sampled Rg values. Sharper, well-defined peaks suggest structural stability and consistency across time or replicates, while broader peaks reflect greater variability in system compactness.

7.2 Complete code

```
import numpy as np
import matplotlib.pyplot as plt
from scipy.stats import gaussian_kde
import os
```

```
def read_rg(file):
```

```
"""
Reads Radius of Gyration data from a GROMACS .xvg file.
Returns time in nanoseconds and Rg values as numpy arrays.
"""
try:
    print(f"Reading file: {file}")
    times, rg_values = [], []
    with open(file, 'r') as f:
        for line in f:
            if line.startswith(('#', '@', ';')) or line.strip() == '':
```

(continues on next page)

(continued from previous page)

```
                continue
            try:
                values = line.split()
                if len(values) >= 2:
                    time, rg_val = map(float, values[:2])
                    times.append(time / 1000)  # Convert picoseconds to nanoseconds
                    rg_values.append(rg_val)
            except ValueError:
                print(f"Warning: could not parse line: {line.strip()}")
                continue
    if len(times) == 0 or len(rg_values) == 0:
        raise ValueError(f"No valid data found in file: {file}")
    return np.array(times), np.array(rg_values)
except Exception as e:
    print(f"Error reading file {file}: {e}")
    return None, None
```

```
def check_simulation_times(*time_arrays):
```

```
"""
Checks if all simulation replicates share the same time points.
Raises ValueError if any mismatch is detected.
"""
for i in range(1, len(time_arrays)):
    if not np.allclose(time_arrays[0], time_arrays[i]):
        raise ValueError(f"Simulation times do not match between replicate 1 and␣
↪replicate {i+1}.")
```

```
def plot_rg(results, output_folder, config):
```

```
"""
Plots time series of Radius of Gyration (Rg) with mean ± std shading.
Supports any number of simulation groups.
"""
labels = config.get('labels', [f'Simulation {i+1}' for i in range(len(results))])
colors = config.get('colors', plt.cm.get_cmap('tab10').colors)
alpha = config.get('alpha', 0.2)
figsize = config.get('figsize', (7, 6))
xlabel = config.get('xlabel', 'Time (ns)')
ylabel = config.get('ylabel', 'Radius of Gyration (nm)')
label_fontsize = config.get('label_fontsize', 12)
xlim = config.get('xlim', None)
ylim = config.get('ylim', None)

plt.figure(figsize=figsize)

for idx, (time, mean, std) in enumerate(results):
    color = colors[idx % len(colors)]
    plt.plot(time, mean, label=labels[idx], color=color, linewidth=2)
    plt.fill_between(time, mean - std, mean + std, color=color, alpha=alpha)

plt.xlabel(xlabel, fontsize=label_fontsize)
plt.ylabel(ylabel, fontsize=label_fontsize)
plt.legend(frameon=False, loc='upper right', fontsize=10)
plt.tick_params(axis='both', which='major', labelsize=10)
```

(continues on next page)

(continued from previous page)

```
if xlim:
    plt.xlim(xlim)
else:
    max_time = max([t[-1] for t, _, _ in results])
    plt.xlim(0, max_time)

if ylim:
    plt.ylim(ylim)

plt.tight_layout()
os.makedirs(output_folder, exist_ok=True)
plt.savefig(os.path.join(output_folder, 'rg_plot.tiff'), dpi=300)
plt.savefig(os.path.join(output_folder, 'rg_plot.png'), dpi=300)
plt.show()
```

```
def plot_density(results, output_folder, config):
```

```
"""
Plots kernel density estimates (KDE) of Radius of Gyration distributions
for each simulation group.
"""
labels = config.get('labels', [f'Simulation {i+1}' for i in range(len(results))])
colors = config.get('colors', plt.cm.get_cmap('tab10').colors)
alpha = config.get('alpha', 0.5)
figsize = config.get('figsize', (6, 6))
xlabel = config.get('xlabel', 'Radius of Gyration (nm)')
ylabel = config.get('ylabel', 'Density')
label_fontsize = config.get('label_fontsize', 12)
xlim = config.get('xlim', None)
ylim = config.get('ylim', None)

plt.figure(figsize=figsize)

for idx, (_, mean, _) in enumerate(results):
    kde = gaussian_kde(mean)
    x_min = xlim[0] if xlim else 0
    x_max = xlim[1] if xlim else max(mean) * 1.1
    x = np.linspace(x_min, x_max, 1000)
    color = colors[idx % len(colors)]
    plt.fill_between(x, kde(x), color=color, alpha=alpha, label=labels[idx])

plt.xlabel(xlabel, fontsize=label_fontsize)
plt.ylabel(ylabel, fontsize=label_fontsize)
plt.legend(frameon=False, loc='upper left', fontsize=10)
plt.tick_params(axis='both', which='major', labelsize=10)

if xlim:
    plt.xlim(xlim)
if ylim:
    plt.ylim(ylim)

plt.tight_layout()
os.makedirs(output_folder, exist_ok=True)
plt.savefig(os.path.join(output_folder, 'rg_density.tiff'), dpi=300)
plt.savefig(os.path.join(output_folder, 'rg_density.png'), dpi=300)
```

(continues on next page)

(continued from previous page)

```
plt.show()
```

```
def rg_analysis(output_folder, *simulation_groups, rg_config=None, density_
↪config=None):
```

```
"""
Main analysis function to process multiple simulation groups with multiple
replicates each. Computes mean and std deviation and plots the results.

Parameters:
- output_folder: directory to save plots
- simulation_groups: variable number of lists, each containing replicate file␣
↪paths for one simulation group
- rg_config: dict with configuration for Rg time series plot
- density_config: dict with configuration for Rg density plot
"""
if rg_config is None:
    rg_config = {}
if density_config is None:
    density_config = {}

results = []

for group_idx, group_files in enumerate(simulation_groups):
    if not group_files:
        print(f"Warning: Simulation group {group_idx + 1} has no files and will be␣
↪skipped.")
        continue

    times_list = []
    rg_list = []

    for file in group_files:
        t, rg = read_rg(file)
        if t is None or rg is None:
            raise ValueError(f"Invalid data in file {file}. Please check the file␣
↪content.")
        times_list.append(t)
        rg_list.append(rg)

    # Check all replicates have matching time points
    check_simulation_times(*times_list)

    mean_rg = np.mean(rg_list, axis=0)
    std_rg = np.std(rg_list, axis=0)
    results.append((times_list[0], mean_rg, std_rg))

if len(results) == 0:
    raise ValueError("No valid simulation groups were provided.")

# Plot results
plot_rg(results, output_folder, rg_config)
plot_density(results, output_folder, density_config)
```

8 Hydrogen Bond Analysis

8.1 Overview

DynamiSpectra provides a comprehensive and versatile analytical framework for quantifying and monitoring hydrogen bond dynamics throughout molecular dynamics simulations. Using input data in standardized .xvg file format, this module enables detailed evaluation of hydrogen bonding interactions that are essential for understanding molecular stability, conformational changes, and interaction patterns.

This analysis offers an in-depth view of hydrogen bond formation and disruption over time. When multiple simulation replicates are available, *DynamiSpectra* calculates averaged hydrogen bond counts and associated variability measures, such as standard deviation, to provide statistically robust insights. The software also supports analysis of individual replicas, offering flexibility when replicate data is limited or unavailable.

Command line in GROMACS to generate .xvg files for the analysis:

```
gmx hbond -s Simulation.tpr -f Simulation.xtc -num Simulation.xvg -tu ns
```

Note: The hydrogen bond analysis module in *DynamiSpectra* also supports the analysis of inter-residue hydrogen bonds within the protein, as well as hydrogen bonds between the protein and ligand. Users simply need to select the appropriate groups during analysis and generate the corresponding .xvg output files.

```
def hbond_analysis(output_folder, *simulation_file_groups, hbond_config=None,
    density_config=None)
```

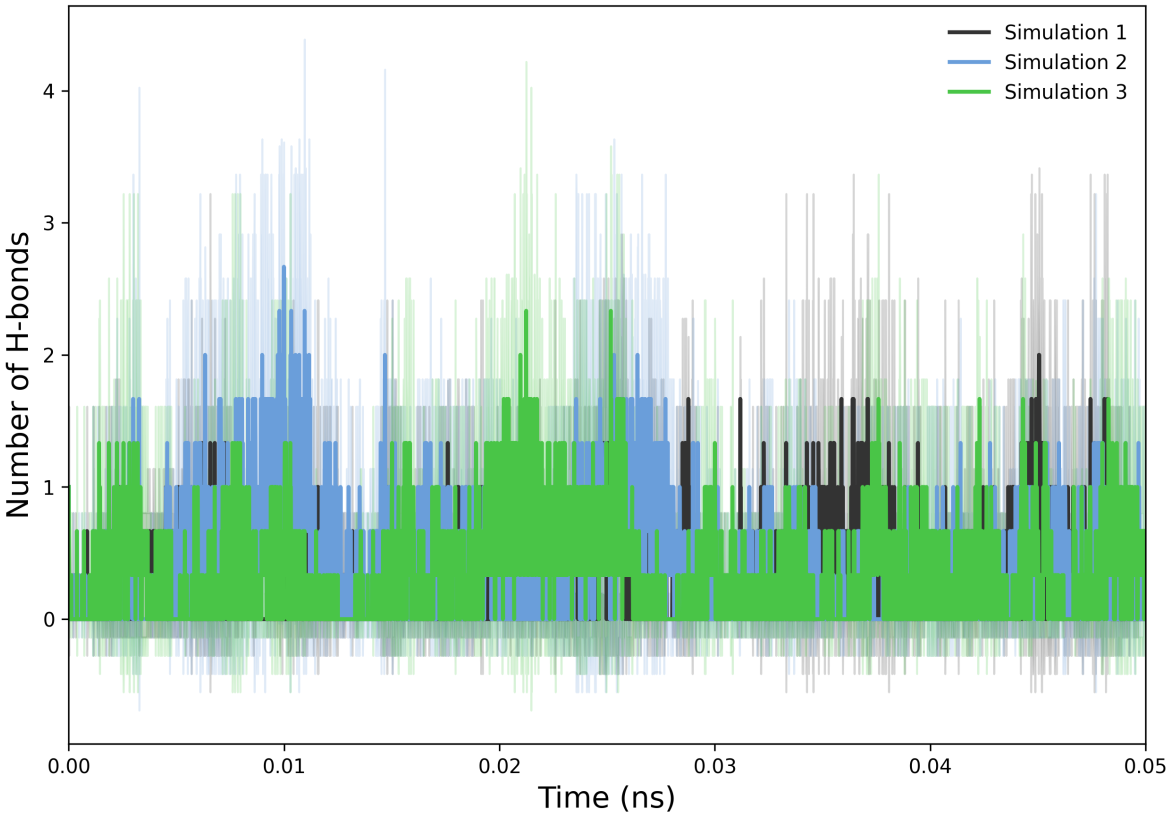

How to interpret: This plot illustrates the temporal evolution of hydrogen bond counts within the system. Stable trends indicate persistent hydrogen bonding and structural integrity, while fluctuations may reflect conformational changes or dynamic instabilities. Comparative analysis across simulations can reveal consistencies or discrepancies attributable to differing initial conditions or simulation parameters. Variations in hydrogen bond numbers over time often correspond to the formation or disruption of critical molecular interactions, providing valuable insight into the system's behavior.

```
def plot_density(results, output_folder, config)
```

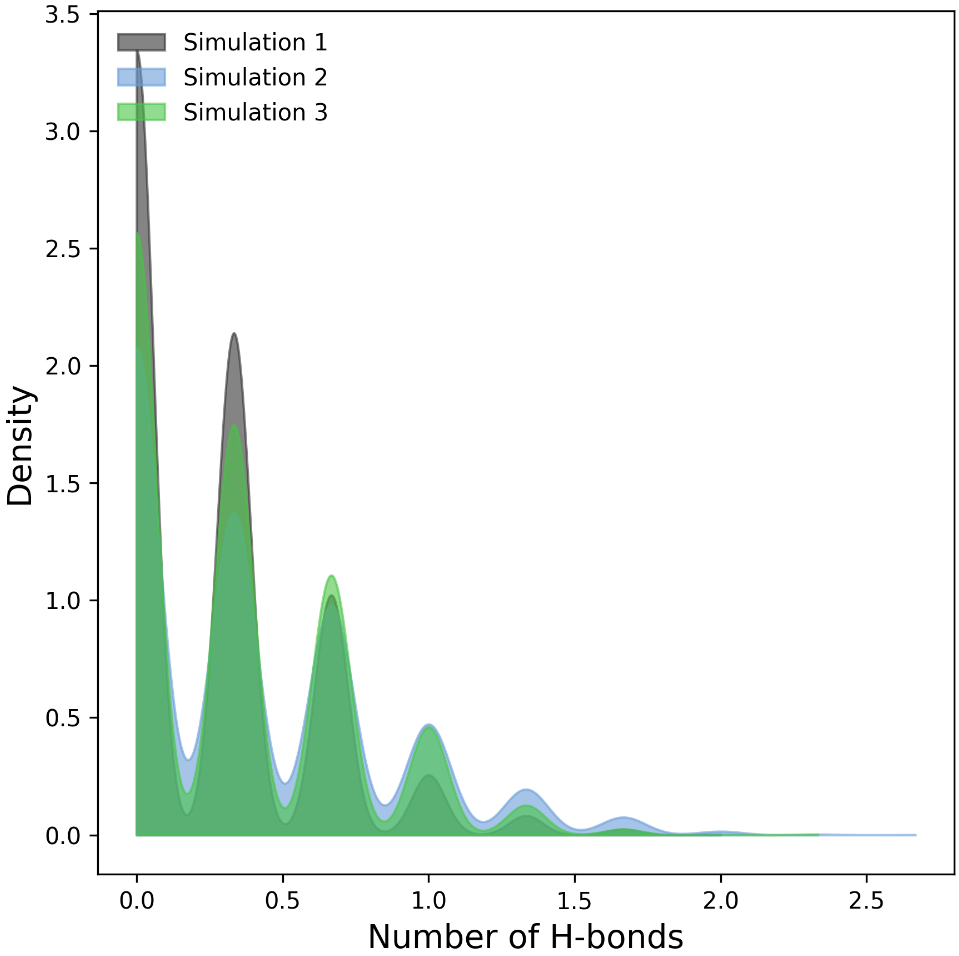

How to interpret: This plot represents the density distribution of hydrogen bonds observed throughout the simulations. The x-axis corresponds to the number of hydrogen bonds, while the y-axis denotes their frequency density. Regions with higher density indicate more frequent occurrences of specific hydrogen bond counts. Comparing density profiles across simulations enables the identification of similarities or divergences in hydrogen bonding patterns, offering valuable insights into the stability and interaction dynamics of the molecular system.

8.2 Complete code

```
import numpy as np
import matplotlib.pyplot as plt
from scipy.stats import gaussian_kde
import os
```

```
def read_hbond(file):
```

```
"""
Reads hydrogen bond data from a GROMACS .xvg file.

Parameters:
- file (str): Path to the .xvg file

Returns:
- times (np.ndarray): Time points (in ns)
- hbonds (np.ndarray): Number of hydrogen bonds at each time
```

(continues on next page)

(continued from previous page)

```
"""
try:
    times, hbonds = [], []
    with open(file, 'r') as f:
        for line in f:
            if line.startswith(('#', '@', ';')) or line.strip() == '':
                continue
            try:
                values = line.split()
                if len(values) >= 2:
                    time, hbond = map(float, values[:2])
                    times.append(time / 1000.0)  # convert ps to ns
                    hbonds.append(hbond)
            except ValueError:
                continue
    if len(times) == 0 or len(hbonds) == 0:
        raise ValueError(f"File {file} does not contain valid data.")
    return np.array(times), np.array(hbonds)
except Exception as e:
    print(f"Error reading file {file}: {e}")
    return None, None
```

```
def check_simulation_times(*time_arrays):
```

```
"""
Ensures all simulation time arrays are aligned across replicates.
"""
for i in range(1, len(time_arrays)):
    if not np.allclose(time_arrays[0], time_arrays[i]):
        raise ValueError(f"Simulation times do not match between file 1 and file
↪{i+1}")
```

```
def plot_hbond(results, output_folder, config):
```

```
"""
Plots average number of hydrogen bonds over time with std deviation.

Parameters:
- results (list of tuples): Each tuple contains (time, mean, std) for a simulation␣
↪group
- output_folder (str): Directory to save the plots
- config (dict): Plot customization options
"""
labels = config.get('labels', [f'Simulation {i+1}' for i in range(len(results))])
colors = config.get('colors', ['#333333', '#6A9EDA', '#54b36a', '#f2444d', '#fc9e19
↪'])
alpha = config.get('alpha', 0.2)
figsize = config.get('figsize', (7, 6))
xlabel = config.get('xlabel', 'Time (ns)')
ylabel = config.get('ylabel', 'Number of H-bonds')
label_fontsize = config.get('label_fontsize', 12)
xlim = config.get('xlim', None)
ylim = config.get('ylim', None)

plt.figure(figsize=figsize)
```

(continues on next page)

(continued from previous page)

```
for i, (time, mean, std) in enumerate(results):
    color = colors[i % len(colors)]
    plt.plot(time, mean, label=labels[i], color=color, linewidth=2)
    plt.fill_between(time, mean - std, mean + std, color=color, alpha=alpha)

plt.xlabel(xlabel, fontsize=label_fontsize)
plt.ylabel(ylabel, fontsize=label_fontsize)
plt.legend(frameon=False, loc='upper right', fontsize=10)
plt.tick_params(axis='both', which='major', labelsize=10)

if xlim:
    plt.xlim(xlim)
else:
    max_time = max([np.max(t) for t, _, _ in results])
    plt.xlim(0, max_time)

if ylim:
    plt.ylim(ylim)

plt.tight_layout()
os.makedirs(output_folder, exist_ok=True)
plt.savefig(os.path.join(output_folder, 'hbond_plot.tiff'), format='tiff', dpi=300)
plt.savefig(os.path.join(output_folder, 'hbond_plot.png'), format='png', dpi=300)
plt.show()
```

```
def plot_density(results, output_folder, config):
```

```
"""
Plots KDE density distributions of H-bond counts.

Parameters:
- results (list of tuples): Each tuple contains (time, mean, std) for a simulation␣
↪group
- output_folder (str): Directory to save the plots
- config (dict): Plot customization options
"""
labels = config.get('labels', [f'Simulation {i+1}' for i in range(len(results))])
colors = config.get('colors', ['#333333', '#6A9EDA', '#54b36a', '#f2444d', '#fc9e19
↪'])
alpha = config.get('alpha', 0.5)
figsize = config.get('figsize', (6, 6))
xlabel = config.get('xlabel', 'Number of H-bonds')
ylabel = config.get('ylabel', 'Density')
label_fontsize = config.get('label_fontsize', 12)
xlim = config.get('xlim', None)
ylim = config.get('ylim', None)

plt.figure(figsize=figsize)

for i, (_, mean, _) in enumerate(results):
    color = colors[i % len(colors)]
    kde = gaussian_kde(mean)
    x = np.linspace(0, max(mean), 1000)
    plt.fill_between(x, kde(x), color=color, alpha=alpha, label=labels[i])
```

(continues on next page)

(continued from previous page)

```
plt.xlabel(xlabel, fontsize=label_fontsize)
plt.ylabel(ylabel, fontsize=label_fontsize)
plt.legend(frameon=False, loc='upper left', fontsize=10)
plt.tick_params(axis='both', which='major', labelsize=10)

if xlim:
    plt.xlim(xlim)
if ylim:
    plt.ylim(ylim)

plt.tight_layout()
os.makedirs(output_folder, exist_ok=True)
plt.savefig(os.path.join(output_folder, 'hbond_density.tiff'), format='tiff',␣
↪dpi=300)
plt.savefig(os.path.join(output_folder, 'hbond_density.png'), format='png',␣
↪dpi=300)
plt.show()
```

```
def hbond_analysis(output_folder, *simulation_file_groups, hbond_config=None,␣
↪density_config=None):
```

```
"""
Main function to perform hydrogen bond analysis.

Parameters:
- output_folder (str): Directory to save the plots
- *simulation_file_groups: Lists of replicate file paths for each simulation group
- hbond_config (dict): Configuration for time-series plot
- density_config (dict): Configuration for KDE density plot
"""
if hbond_config is None:
    hbond_config = {}
if density_config is None:
    density_config = {}

def process_group(file_paths):
    times, hbonds = [], []
    for file in file_paths:
        time, hbond = read_hbond(file)
        if time is None or hbond is None:
            raise ValueError(f"Error reading file: {file}")
        times.append(time)
        hbonds.append(hbond)
    check_simulation_times(*times)
    return times[0], np.mean(hbonds, axis=0), np.std(hbonds, axis=0)

results = []
for group in simulation_file_groups:
    if group:
        result = process_group(group)
        results.append(result)

if not results:
    raise ValueError("At least one simulation group must be provided.")

plot_hbond(results, output_folder, hbond_config)
```

(continues on next page)

(continued from previous page)

```
plot_density(results, output_folder, density_config)
```

9 Solvent Accessible Surface Area

9.1 Overview

DynamiSpectra provides a comprehensive Solvent Accessible Surface Area (SASA) analysis module for molecular dynamics simulations. This module supports both individual replica analysis and the calculation of statistical metrics (mean and standard deviation) across multiple simulation replicas. The resulting plots display the average SASA over time with shaded regions representing the variability across replicates. This enables users to assess trends in solvent exposure and compare results across different conditions or systems.

Command line in GROMACS to generate .xvg files for the analysis:

```
gmx sasa -s Simulation.tpr -f Simulation.xtc -o sasa_simulation.xvg -probe 0.14
```

```
def sasa_analysis(output_folder, *simulation_files_groups, sasa_config=None,␣
↪density_config=None)
```

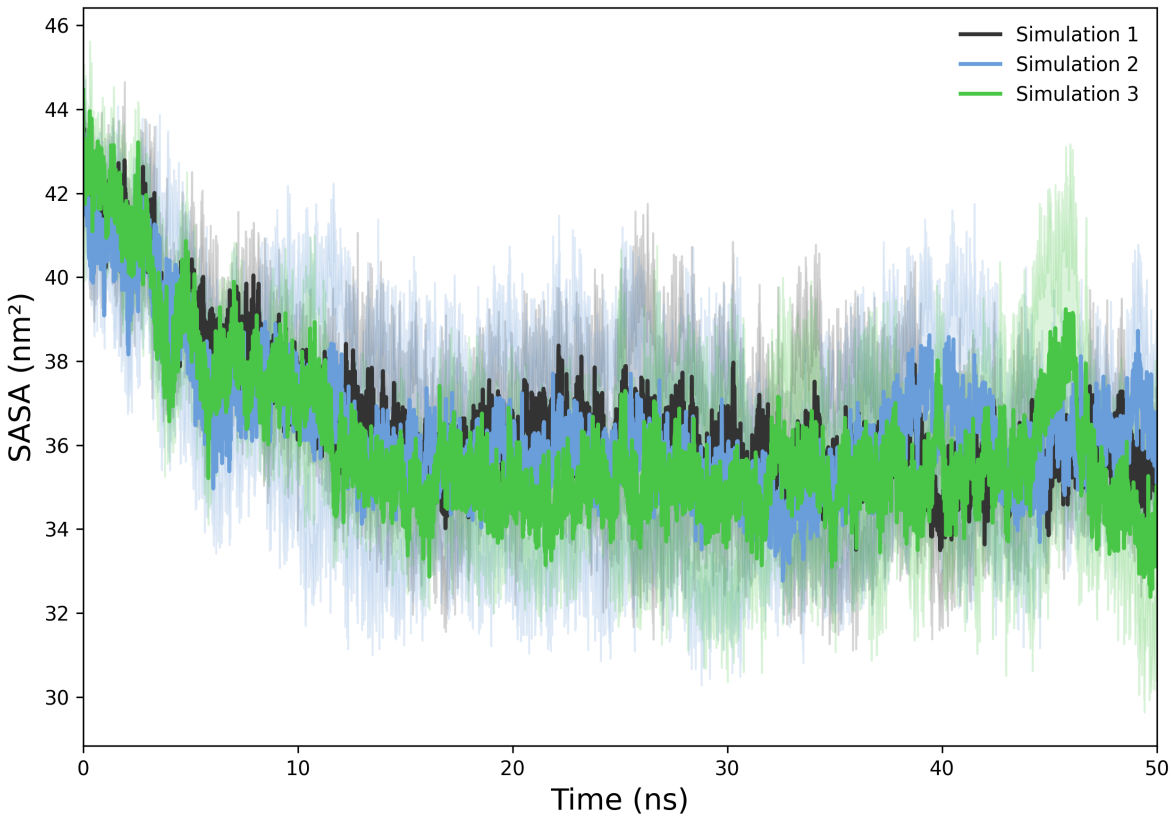

Interpretation guidance This graph shows the SASA over time, with the mean and standard deviation across replicas. A stable SASA indicates that the system maintains a consistent solvent-exposed surface area. Sudden increases may reflect unfolding or expansion, while decreases can suggest compaction or structural collapse. Monitoring SASA trends helps reveal conformational changes during the simulation.

```
def plot_density(means_list, output_folder, config)
```

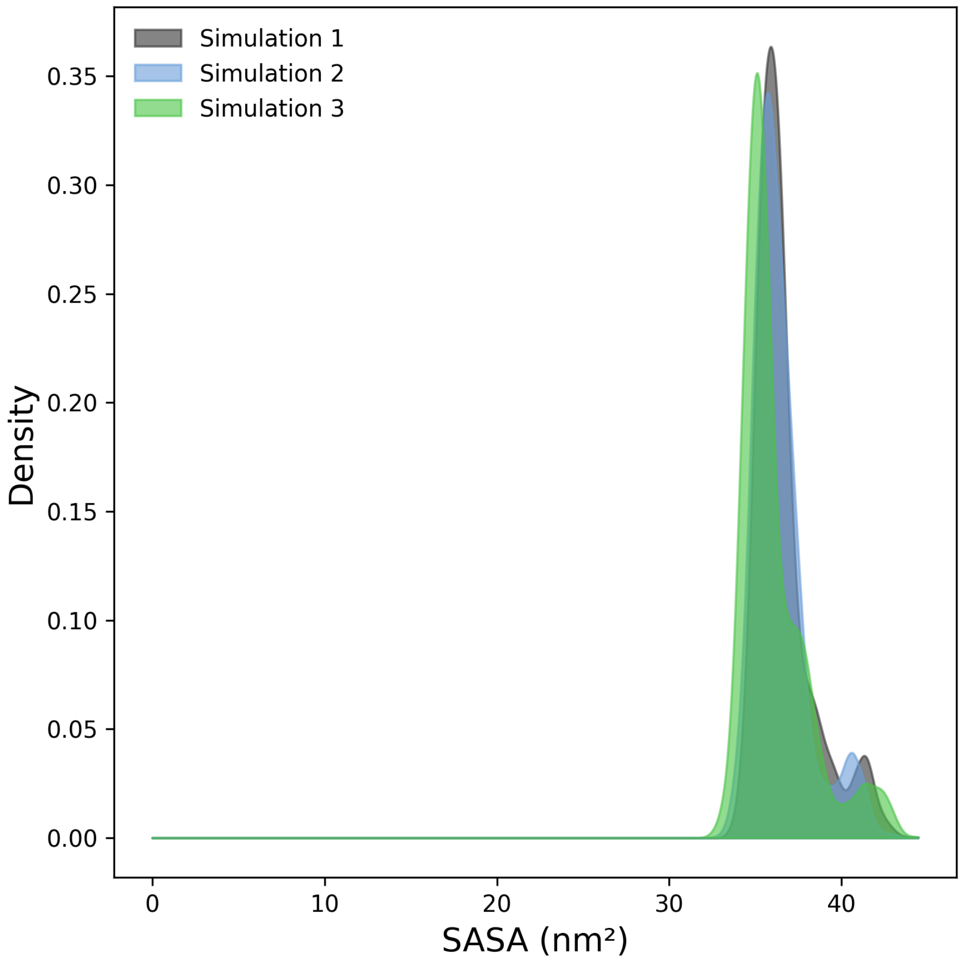

Interpretation guidance: This graph shows the probability distribution of SASA values across the simulation. Peaks indicate the most frequently sampled solvent-accessible areas. Narrow, sharp peaks suggest structural stability, while broader distributions reflect greater conformational variability. Comparing curves across simulations can reveal differences in surface exposure behavior.

9.2 Complete code

```
import numpy as np
import matplotlib.pyplot as plt
from scipy.stats import gaussian_kde
import os
```

```
def read_sasa(file):
```

```
"""
Reads SASA data from a GROMACS .xvg file.
Converts time from ps to ns.
Returns numpy arrays of time and SASA.
"""
try:
    print(f"Reading file: {file}")
    times, sasas = [], []
    with open(file, 'r') as f:
        for line in f:
```

(continues on next page)

(continued from previous page)

```
            # Skip comments and empty lines
            if line.startswith(('#', '@', ';')) or line.strip() == '':
                continue
            try:
                values = line.split()
                if len(values) >= 2:
                    time, sasa = map(float, values[:2])
                    times.append(time / 1000)  # convert ps to ns
                    sasas.append(sasa)
            except ValueError:
                print(f"Error processing line: {line.strip()}")
                continue
    if len(times) == 0 or len(sasas) == 0:
        raise ValueError(f"File {file} does not contain valid data.")
    return np.array(times), np.array(sasas)
except Exception as e:
    print(f"Error reading file {file}: {e}")
    return None, None
```

```
def check_simulation_times(*time_arrays):
```

```
"""
Checks if all provided time arrays are approximately equal.
Raises ValueError if any mismatch is found.
"""
for i in range(1, len(time_arrays)):
    if not np.allclose(time_arrays[0], time_arrays[i]):
        raise ValueError(f"Simulation times do not match between file 1 and file
↪{i+1}")
```

```
def plot_sasa(times_list, means_list, stds_list, output_folder, config):
```

```
"""
Plots SASA time series with mean ± std deviation shaded regions.
Accepts dynamic number of simulation groups.
"""
labels = config.get('labels', [f'Simulation {i+1}' for i in range(len(times_
↪list))])
colors = config.get('colors', ['#333333', '#6A9EDA', '#54b36a', '#e377c2', '#8c564b
↪', '#17becf'])
alpha = config.get('alpha', 0.2)
figsize = config.get('figsize', (7, 6))
xlabel = config.get('xlabel', 'Time (ns)')
ylabel = config.get('ylabel', 'SASA (nm²)')
label_fontsize = config.get('label_fontsize', 12)
xlim = config.get('xlim', None)
ylim = config.get('ylim', None)

plt.figure(figsize=figsize)

for i, (t, m, s) in enumerate(zip(times_list, means_list, stds_list)):
    if t is not None:
        color = colors[i % len(colors)]
        label = labels[i] if i < len(labels) else f'Simulation {i+1}'
        plt.plot(t, m, label=label, color=color, linewidth=2)
```

(continues on next page)

(continued from previous page)

```
        plt.fill_between(t, m - s, m + s, color=color, alpha=alpha)

plt.xlabel(xlabel, fontsize=label_fontsize)
plt.ylabel(ylabel, fontsize=label_fontsize)
plt.legend(frameon=False, loc='upper right', fontsize=10)
plt.tick_params(axis='both', which='major', labelsize=10)

if xlim:
    plt.xlim(xlim)
else:
    all_times = [t for t in times_list if t is not None]
    plt.xlim(0, max([t[-1] for t in all_times]))

if ylim:
    plt.ylim(ylim)

plt.tight_layout()
os.makedirs(output_folder, exist_ok=True)
plt.savefig(os.path.join(output_folder, 'sasa_plot.tiff'), format='tiff', dpi=300)
plt.savefig(os.path.join(output_folder, 'sasa_plot.png'), format='png', dpi=300)
plt.show()
```

```
def plot_density(means_list, output_folder, config):
```

```
"""
Plots KDE density of SASA distributions for each simulation group.
Accepts dynamic number of simulation groups.
"""
labels = config.get('labels', [f'Simulation {i+1}' for i in range(len(means_
↪list))])
colors = config.get('colors', ['#333333', '#6A9EDA', '#54b36a', '#e377c2', '#8c564b
↪', '#17becf'])
alpha = config.get('alpha', 0.5)
figsize = config.get('figsize', (6, 6))
xlabel = config.get('xlabel', 'SASA (nm²)')
ylabel = config.get('ylabel', 'Density')
label_fontsize = config.get('label_fontsize', 12)
xlim = config.get('xlim', None)
ylim = config.get('ylim', None)

plt.figure(figsize=figsize)

for i, m in enumerate(means_list):
    if m is not None:
        color = colors[i % len(colors)]
        label = labels[i] if i < len(labels) else f'Simulation {i+1}'
        kde = gaussian_kde(m)
        x = np.linspace(0, max(m), 1000)
        plt.fill_between(x, kde(x), color=color, alpha=alpha, label=label)

plt.xlabel(xlabel, fontsize=label_fontsize)
plt.ylabel(ylabel, fontsize=label_fontsize)
plt.legend(frameon=False, loc='upper left', fontsize=10)
plt.tick_params(axis='both', which='major', labelsize=10)

if xlim:
```

(continues on next page)

(continued from previous page)

```
    plt.xlim(xlim)
if ylim:
    plt.ylim(ylim)

plt.tight_layout()
os.makedirs(output_folder, exist_ok=True)
plt.savefig(os.path.join(output_folder, 'density_plot.tiff'), format='tiff',␣
↪dpi=300)
plt.savefig(os.path.join(output_folder, 'density_plot.png'), format='png', dpi=300)
plt.show()
```

```
def sasa_analysis(output_folder, *simulation_files_groups, sasa_config=None,␣
↪density_config=None):
```

```
"""
Main function to analyze SASA from multiple simulation groups and replicas.
Each group is a list of replicate file paths.
Computes mean ± std and generates plots.
"""
if sasa_config is None:
    sasa_config = {}
if density_config is None:
    density_config = {}

def process_group(file_paths):
    """
    Processes one simulation group (multiple replicas).
    Reads SASA data, checks time consistency, returns mean and std.
    """
    times, sasas = [], []
    for file in file_paths:
        time, sasa = read_sasa(file)
        if time is None or sasa is None:
            raise ValueError(f"Error reading file: {file}.")
        times.append(time)
        sasas.append(sasa)
    check_simulation_times(*times)
    return times[0], np.mean(sasas, axis=0), np.std(sasas, axis=0)

results = []
for group in simulation_files_groups:
    if group:
        time, mean, std = process_group(group)
        results.append((time, mean, std))

if not results:
    raise ValueError("You must provide at least one group of simulation files.")

# Unpack results dynamically for plotting
times_list, means_list, stds_list = zip(*results)

# Plot time series for all groups
plot_sasa(times_list, means_list, stds_list, output_folder, sasa_config)
# Plot KDE densities for all groups
plot_density(means_list, output_folder, density_config)
```

10 Salt Bridge

10.1 Overview

DynamiSpectra offers a robust salt bridge analysis tool for molecular dynamics simulations. This module tracks the minimum distances between charged groups in the protein and ligand over time, using input data in standardized .xvg file format. It supports analysis of individual replicas as well as averaging across multiple replicas, providing mean distances and variability (standard deviation). The generated plots visualize the temporal evolution of these distances, with shaded regions indicating the variability, facilitating clear interpretation of salt bridge stability.

Command line in GROMACS to generate .xvg files for the analysis:

```
gmx mindist -f Simulation.xtc -s Simulation.tpr -n index.ndx -od Simulation.xvg -
↪on saltbridge_csimulation.xvg -group -d 0.4
```

```
def saltbridge_analysis(output_folder, *simulation_files_groups, saltbridge_
↪config=None, density_config=None)
```

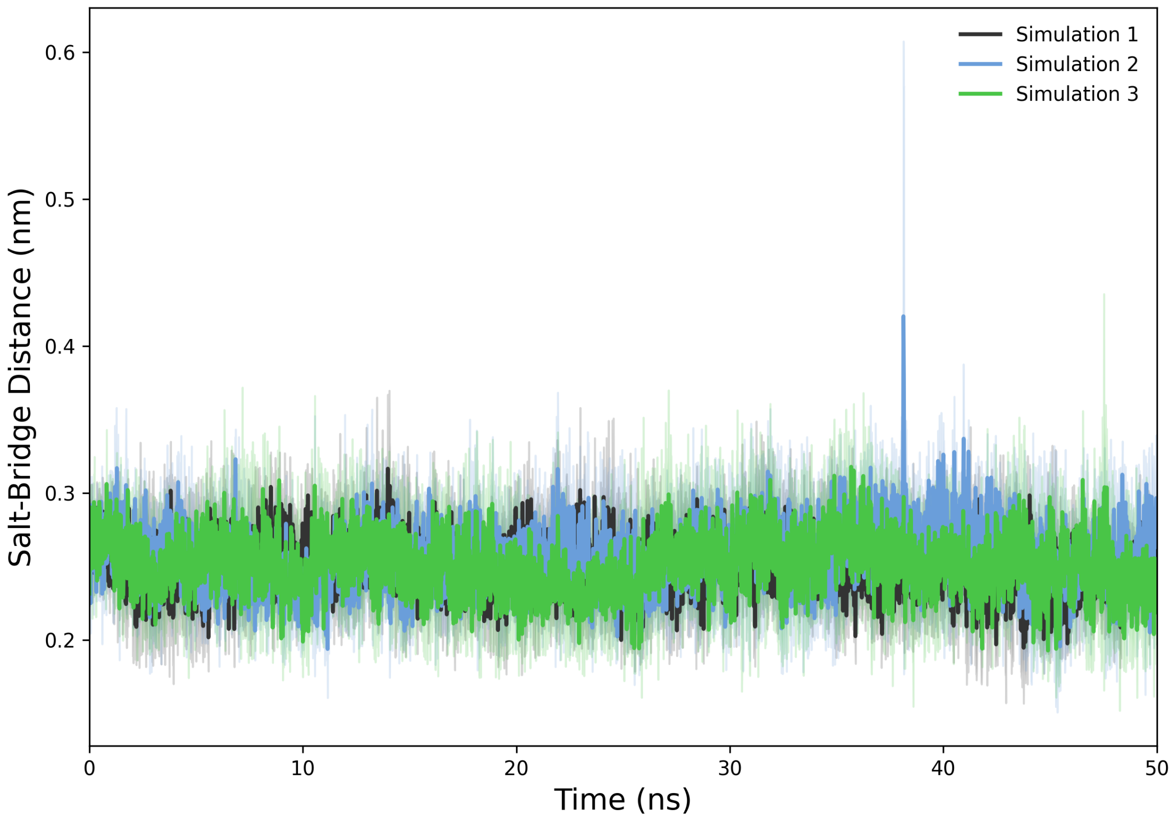

Interpretation guidance: This graph shows the evolution of the minimum distance between charged groups of the ligand and the protein over time, based on data from .xvg files. When analyzing it, observe if the distance stabilizes below a threshold indicating the presence of a stable salt bridge interaction. Small fluctuations around a consistent low distance suggest a persistent salt bridge, while large increases or irregular spikes may indicate the breaking or absence of the interaction. Comparing distance values with known thresholds or reference data helps determine the significance of the salt bridge throughout the simulation.

```
def plot_density(results, output_folder, config)
```

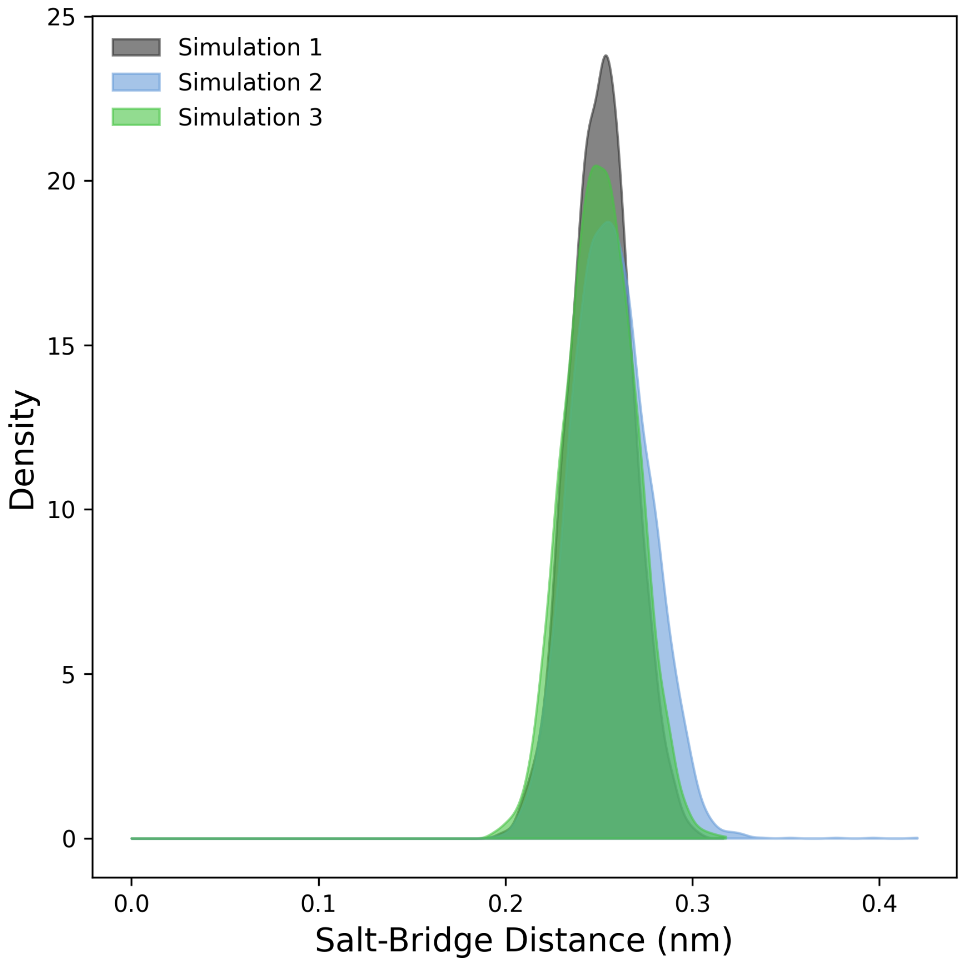

Interpretation guidance: This plot shows the distribution of salt bridge distances during the simulation. Peaks at lower distances indicate stable interactions, while broader or shifted peaks suggest more variability or disruption. Comparing curves reveals differences in salt bridge persistence across simulations.

10.2 Complete code

```
import numpy as np
import matplotlib.pyplot as plt
from scipy.stats import gaussian_kde
import os
```

```
def read_saltbridge(file):
```

```
try:
    print(f"Reading file: {file}")
    times, distances = [], []
    with open(file, 'r') as f:
        for line in f:
            if line.startswith(('#', '@', ';')) or line.strip() == '':
                continue
            try:
                values = line.split()
                if len(values) >= 2:
                    time, distance = map(float, values[:2])
```

(continues on next page)

(continued from previous page)

```
                    times.append(time / 1000.0)  # Convert ps to ns
                    distances.append(distance)
            except ValueError:
                print(f"Error processing line: {line.strip()}")
                continue
    if len(times) == 0 or len(distances) == 0:
        raise ValueError(f"File {file} does not contain valid data.")
    return np.array(times), np.array(distances)
except Exception as e:
    print(f"Error reading file {file}: {e}")
    return None, None
```

```
def check_simulation_times(*time_arrays):
```

```
for i in range(1, len(time_arrays)):
    if not np.allclose(time_arrays[0], time_arrays[i]):
        raise ValueError(f"Simulation times do not match between file 1 and file
↪{i+1}")
```

```
def plot_saltbridge(results, output_folder, config):
```

```
labels = config.get('labels', [f'Simulation {i+1}' for i in range(len(results))])
colors = config.get('colors', ['#333333', '#6A9EDA', '#54b36a', '#f39c12', '#8e44ad
↪'])
alpha = config.get('alpha', 0.2)
figsize = config.get('figsize', (7, 6))
xlabel = config.get('xlabel', 'Time (ns)')
ylabel = config.get('ylabel', 'Salt-Bridge Distance (nm)')
label_fontsize = config.get('label_fontsize', 12)
xlim = config.get('xlim', None)
ylim = config.get('ylim', None)

plt.figure(figsize=figsize)

for i, (time, mean, std) in enumerate(results):
    plt.plot(time, mean, label=labels[i], color=colors[i % len(colors)],␣
↪linewidth=2)
    plt.fill_between(time, mean - std, mean + std, color=colors[i % len(colors)],␣
↪alpha=alpha)

plt.xlabel(xlabel, fontsize=label_fontsize)
plt.ylabel(ylabel, fontsize=label_fontsize)
plt.legend(frameon=False, loc='upper right', fontsize=10)
plt.tick_params(axis='both', which='major', labelsize=10)

if xlim:
    plt.xlim(xlim)
else:
    max_time = max([t[-1] for t, _, _ in results])
    plt.xlim(0, max_time)

if ylim:
    plt.ylim(ylim)

plt.tight_layout()
```

(continues on next page)

(continued from previous page)

```
os.makedirs(output_folder, exist_ok=True)
plt.savefig(os.path.join(output_folder, 'saltbridge_plot.tiff'), format='tiff',␣
↪dpi=300)
plt.savefig(os.path.join(output_folder, 'saltbridge_plot.png'), format='png',␣
↪dpi=300)
plt.show()
```

```
def plot_density(results, output_folder, config):
```

```
labels = config.get('labels', [f'Simulation {i+1}' for i in range(len(results))])
colors = config.get('colors', ['#333333', '#6A9EDA', '#54b36a', '#f39c12', '#8e44ad
↪'])
alpha = config.get('alpha', 0.5)
figsize = config.get('figsize', (6, 6))
xlabel = config.get('xlabel', 'Salt-Bridge Distance (nm)')
ylabel = config.get('ylabel', 'Density')
label_fontsize = config.get('label_fontsize', 12)
xlim = config.get('xlim', None)
ylim = config.get('ylim', None)

plt.figure(figsize=figsize)

for i, (_, mean, _) in enumerate(results):
    kde = gaussian_kde(mean)
    x = np.linspace(0, max(mean), 1000)
    plt.fill_between(x, kde(x), color=colors[i % len(colors)], alpha=alpha,␣
↪label=labels[i])

plt.xlabel(xlabel, fontsize=label_fontsize)
plt.ylabel(ylabel, fontsize=label_fontsize)
plt.legend(frameon=False, loc='upper left', fontsize=10)
plt.tick_params(axis='both', which='major', labelsize=10)

if xlim:
    plt.xlim(xlim)
if ylim:
    plt.ylim(ylim)

plt.tight_layout()
os.makedirs(output_folder, exist_ok=True)
plt.savefig(os.path.join(output_folder, 'saltbridge_density.tiff'), format='tiff',␣
↪dpi=300)
plt.savefig(os.path.join(output_folder, 'saltbridge_density.png'), format='png',␣
↪dpi=300)
plt.show()
```

```
def saltbridge_analysis(output_folder, *simulation_files_groups, saltbridge_
↪config=None, density_config=None):
```

```
if saltbridge_config is None:
    saltbridge_config = {}
if density_config is None:
    density_config = {}

def process_group(file_paths):
```

(continues on next page)

(continued from previous page)

```
    times, distances = [], []
    for file in file_paths:
        time, dist = read_saltbridge(file)
        if time is None or dist is None:
            raise ValueError(f"Error reading file: {file}")
        times.append(time)
        distances.append(dist)
    check_simulation_times(*times)
    return times[0], np.mean(distances, axis=0), np.std(distances, axis=0)

results = []
for group in simulation_files_groups:
    if group:
        time, mean, std = process_group(group)
        results.append((time, mean, std))

if not results:
    raise ValueError("You must provide at least one group of simulation files.")

plot_saltbridge(results, output_folder, saltbridge_config)
plot_density(results, output_folder, density_config)
```

11 Protein-Ligand Contacts

11.1 Overview

DynamiSpectra offers a robust analytical framework to quantify the number of contacts between protein and ligand throughout molecular dynamics simulations, utilizing input data in .xvg file format. This analysis facilitates a detailed characterization of intermolecular interactions and binding dynamics.

The software's graphical interface enables the visualization of the average number of contacts calculated across multiple simulation replicates, with the associated standard deviation represented as a shaded region illustrating variability. Furthermore, *DynamiSpectra* accommodates the analysis of individual simulation replicas, allowing users to generate plots from a single dataset when multiple replicas are unavailable or unnecessary.

Command line in GROMACS to generate .xvg files for the analysis:

```
gmx mindist -f Simulation.xtc -s Simulation.tpr -n index.ndx -on Contacts.xvg -d 0.
↪35
```

```
def contact_analysis(output_folder, *simulation_file_groups, contact_config=None,␣
↪density_config=None)
```

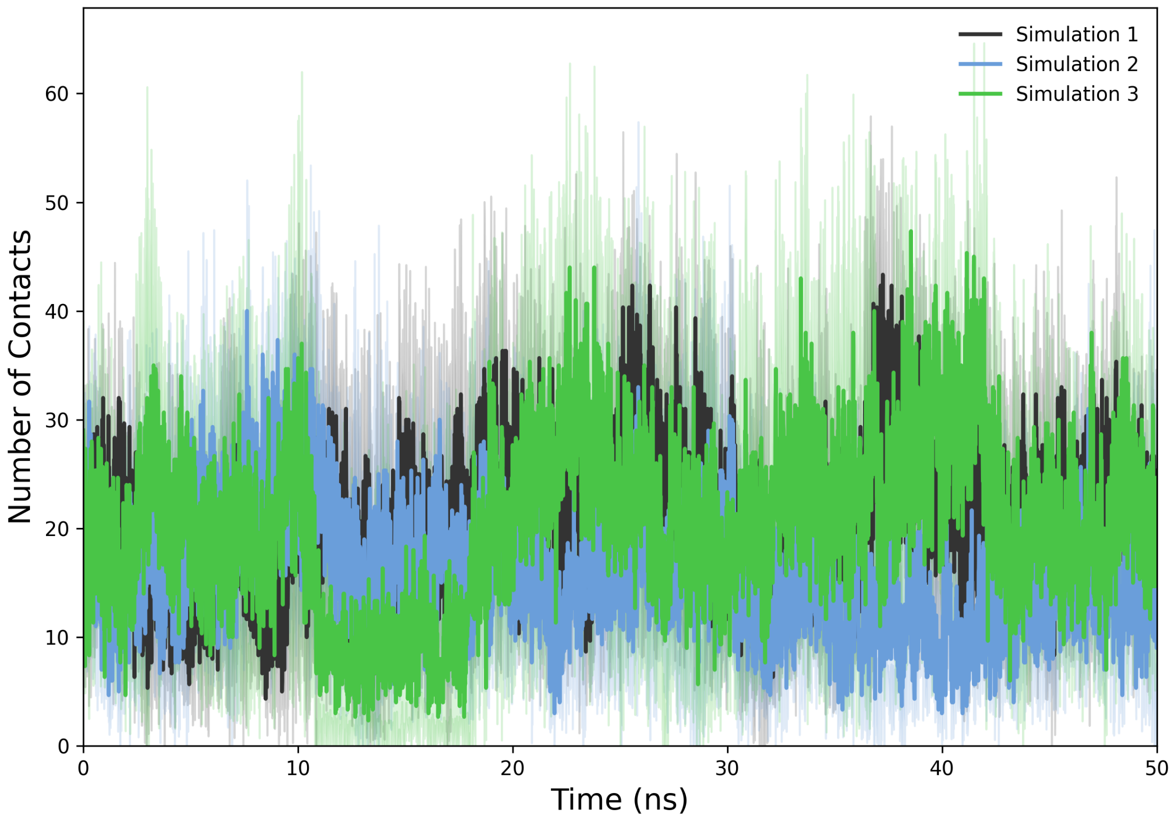

Interpretation guidance: The resulting plots depict the temporal evolution of protein-ligand contacts. A stable profile suggests persistent interactions and binding stability, whereas notable deviations may indicate conformational changes, ligand dissociation events, or alternative binding modes.

```
def plot_contact_density(results, output_folder, config=None)
```

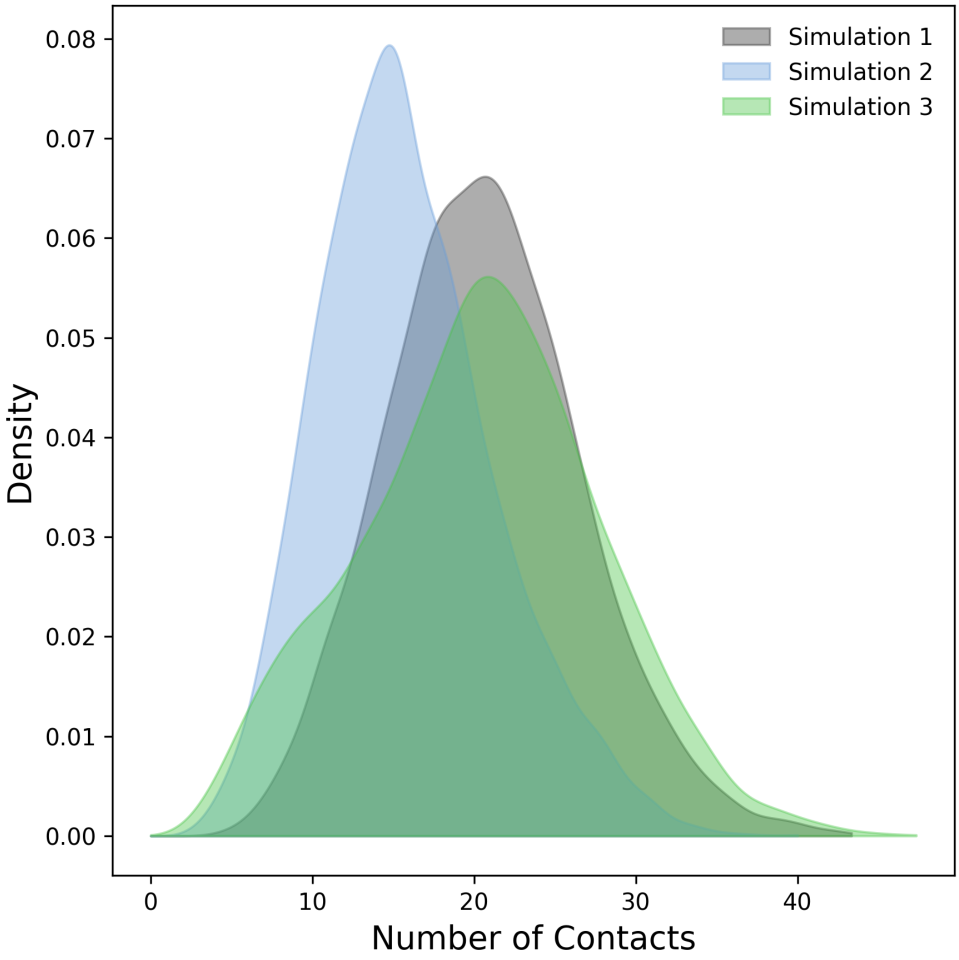

Interpretation guidance: This plot depicts the density distribution derived from the temporal profile of protein-ligand contacts. Peaks in the distribution correspond to regions exhibiting a higher frequency of interactions. Broader peaks indicate a more heterogeneous or variable contact pattern, while sharper, more pronounced peaks reflect stable and well-defined interaction regions between the protein and ligand.

11.2 Complete code

```
import numpy as np
import matplotlib.pyplot as plt
from scipy.stats import gaussian_kde
import os
```

```
def read_contacts(file):
```

```
"""
Reads contact data from a .xvg file.
Skips header lines and extracts time (in ns) and contact values.

Parameters:
-----------
file : str
    Path to the .xvg file

Returns:
```

(continues on next page)

(continued from previous page)

```
--------
times : np.ndarray
    Array of time points in nanoseconds.
contacts : np.ndarray
    Array of contact counts at each time point.
"""
try:
    times = []
    contacts = []
    with open(file, 'r') as f:
        for line in f:
            # Skip metadata or comment lines (start with #, @, or ;)
            if line.startswith(('#', '@', ';')) or line.strip() == '':
                continue
            try:
                values = line.split()
                if len(values) >= 2:
                    time_ps, contact_val = map(float, values[:2])
                    times.append(time_ps / 1000.0)  # Convert from picoseconds to␣
↪nanoseconds
                    contacts.append(contact_val)
            except ValueError:
                # If conversion to float fails, skip the line
                continue

    # Check if any valid data was read
    if len(times) == 0 or len(contacts) == 0:
        raise ValueError(f"File {file} does not contain valid data.")

    return np.array(times), np.array(contacts)

except Exception as e:
    # Print error if something goes wrong while reading the file
    print(f"Error reading file {file}: {e}")
    return None, None
```

```
def check_simulation_times(*time_arrays):
```

```
"""
Checks if all simulation time arrays are consistent (equal).
Raises an error if times do not match to avoid misaligned averaging.
"""
for i in range(1, len(time_arrays)):
    # Compare current time array with the first one
    if not np.allclose(time_arrays[0], time_arrays[i]):
        raise ValueError(f"Simulation times do not match between file 1 and file
↪{i+1}")
```

```
def plot_contacts(results, output_folder, config=None):
```

```
"""
Plots the mean number of contacts over time with shaded std deviation.

Parameters:
-----------
```

(continues on next page)

(continued from previous page)

```
results : list of tuples
    Each tuple is (time_array, mean_contacts, std_contacts) for one simulation␣
↪group.
output_folder : str
    Folder path to save the plots.
config : dict, optional
    Plot configuration dictionary (colors, labels, axis labels, etc.)
"""
plt.figure(figsize=config.get('figsize', (9, 6)))

# Plot mean ± std for each simulation group
for idx, (time, mean, std) in enumerate(results):
    label = config['labels'][idx] if config and 'labels' in config else f
↪'Simulation {idx+1}'
    color = config['colors'][idx] if config and 'colors' in config else None
    alpha = config.get('alpha', 0.2)

    # Plot average contact line
    plt.plot(time, mean, label=label, color=color, linewidth=2)

    # Plot shaded region for standard deviation
    plt.fill_between(time, mean - std, mean + std, color=color, alpha=alpha)

# Axis labels and formatting
plt.xlabel(config.get('xlabel', 'Time (ns)'), fontsize=config.get('label_fontsize',
↪ 12))
plt.ylabel(config.get('ylabel', 'Number of Contacts'), fontsize=config.get('label_
↪fontsize', 12))
plt.legend(frameon=False, loc='upper right', fontsize=10)
plt.tick_params(axis='both', which='major', labelsize=10)

# Dynamically adjust axis limits based on data
max_time = max([np.max(time) for time, _, _ in results])
max_val = max([np.max(mean + std) for _, mean, std in results])
plt.xlim(0, max_time)
plt.ylim(0, max_val * 1.05)  # Add 5% margin above max value

plt.tight_layout()

# Create output folder if it doesn't exist
os.makedirs(output_folder, exist_ok=True)

# Save plots in TIFF and PNG formats
plt.savefig(os.path.join(output_folder, 'contacts_plot.tiff'), dpi=300)
plt.savefig(os.path.join(output_folder, 'contacts_plot.png'), dpi=300)

plt.show()
```

```
def plot_contact_density(results, output_folder, config=None):
```

```
"""
Plots kernel density estimates of the contact number distributions.

Parameters:
-----------
results : list of tuples
```

(continues on next page)

(continued from previous page)

```
    Each tuple is (time_array, mean_contacts, std_contacts).
    Only the mean_contacts array is used here for density estimation.
output_folder : str
    Folder path to save the density plots.
config : dict, optional
    Plot configuration dictionary.
"""
plt.figure(figsize=config.get('figsize', (6, 6)))

# Plot KDE for each simulation group's mean contact values
for idx, (_, mean, _) in enumerate(results):
    kde = gaussian_kde(mean)  # Perform KDE on mean contacts
    x_vals = np.linspace(0, max(mean), 1000)  # Range for plotting KDE

    label = config['labels'][idx] if config and 'labels' in config else f
↪'Simulation {idx+1}'
    color = config['colors'][idx] if config and 'colors' in config else None
    alpha = config.get('alpha', 0.5)

    # Plot KDE curve as a filled area
    plt.fill_between(x_vals, kde(x_vals), color=color, alpha=alpha, label=label)

# Axis labels and formatting
plt.xlabel(config.get('xlabel', 'Number of Contacts'), fontsize=config.get('label_
↪fontsize', 12))
plt.ylabel(config.get('ylabel', 'Density'), fontsize=config.get('label_fontsize',␣
↪12))
plt.legend(frameon=False, loc='upper right', fontsize=10)
plt.tight_layout()

# Save density plots
plt.savefig(os.path.join(output_folder, 'contacts_density.tiff'), dpi=300)
plt.savefig(os.path.join(output_folder, 'contacts_density.png'), dpi=300)

plt.show()
```

```
def contact_analysis(output_folder, *simulation_file_groups, contact_config=None,␣
↪density_config=None):
```

```
"""
Main function to process multiple simulation groups and generate plots.

Parameters:
-----------
output_folder : str
    Directory where the plots will be saved.
*simulation_file_groups : list of lists
    Each element is a list of file paths for replicates of a simulation group.
contact_config : dict, optional
    Configuration for the contact vs time plot.
density_config : dict, optional
    Configuration for the density plot.
"""
def process_group(file_paths):
    # Read time and contact data from all replicas in the group
    times = []
```

(continues on next page)

(continued from previous page)

```
    contacts = []
    for file in file_paths:
        time, contact_val = read_contacts(file)
        times.append(time)
        contacts.append(contact_val)

    # Ensure all replicas are aligned in time
    check_simulation_times(*times)

    # Compute mean and std across replicas
    mean_contacts = np.mean(contacts, axis=0)
    std_contacts = np.std(contacts, axis=0)
    return times[0], mean_contacts, std_contacts

results = []
for group in simulation_file_groups:
    if group:
        # Process each simulation group (set of replicas)
        time, mean, std = process_group(group)
        results.append((time, mean, std))

# If at least one group is processed, generate plots
if len(results) >= 1:
    plot_contacts(results, output_folder, config=contact_config)
    plot_contact_density(results, output_folder, config=density_config)
else:
    raise ValueError("At least one simulation group is required.")
```

12 Protein-Ligand Minimal Distance

12.1 Overview

DynamiSpectra provides a rigorous and versatile analytical framework designed to quantify and monitor the dynamic distances between protein and ligand molecules throughout the course of molecular dynamics simulations. Utilizing input data in standardized .xvg file format, this module enables researchers to precisely evaluate intermolecular spatial relationships, which are critical for understanding binding affinity, conformational changes, and interaction stability.

This analysis offers a comprehensive perspective on how the proximity between the protein and ligand evolves over time, allowing identification of transient interactions, stable binding modes, or dissociation events. By incorporating multiple simulation replicates, *DynamiSpectra* calculates averaged distance profiles along with corresponding measures of variability, such as standard deviation, to provide statistically meaningful insights. Additionally, the software accommodates the evaluation of individual simulation replicas, enabling flexible application where replicate data may be limited or unnecessary.

Command line in GROMACS to generate .xvg files for the analysis:

```
gmx mindist -f Simulation.xtc -s Simulation.tpr -n index.ndx -od Distance.xvg -d 0.
↪35
```

```
def distance_analysis(output_folder, *simulation_file_groups, distance_config=None,
↪ density_config=None)
```

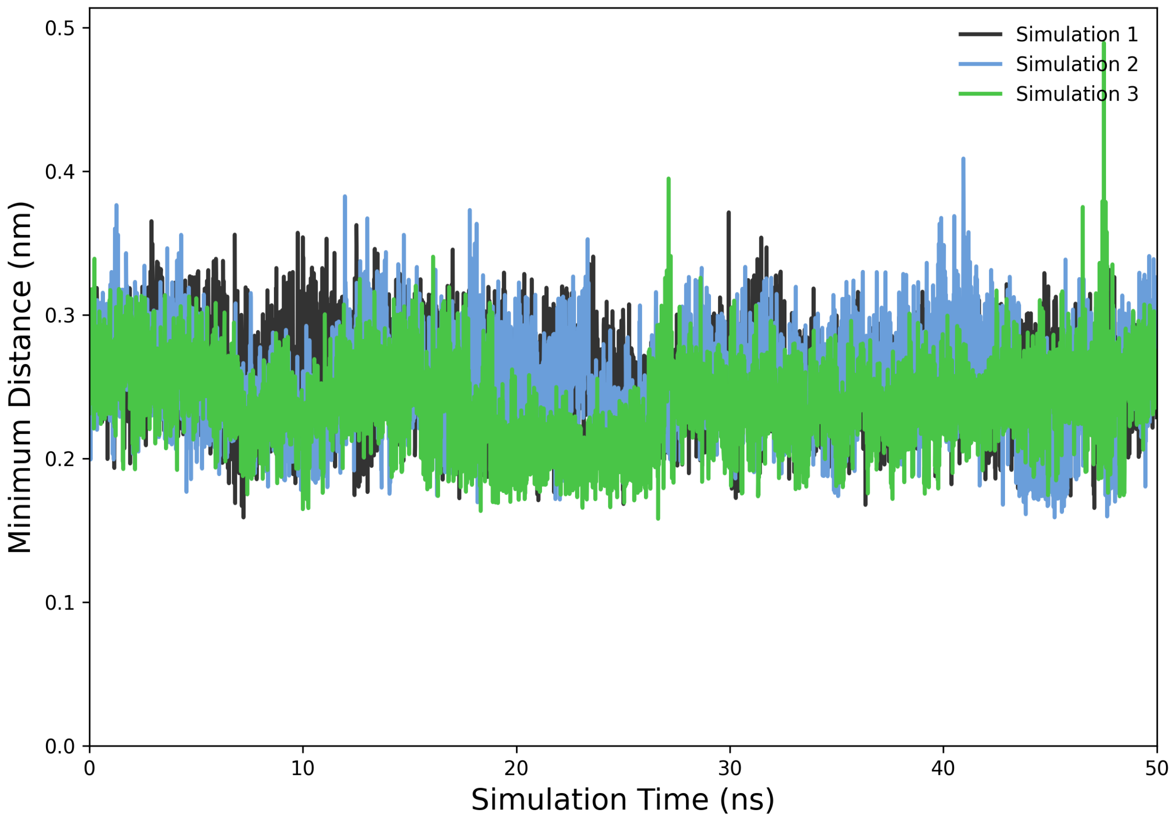

Interpretation guidance: The resulting plots depict the temporal evolution of the minimal distances between protein and ligand atoms. Consistently low minimal distances suggest stable close contacts or binding, while significant increases or fluctuations may indicate conformational changes, ligand dissociation, or transient interactions

```
def plot_distance_density(results, output_folder, config=None)
```

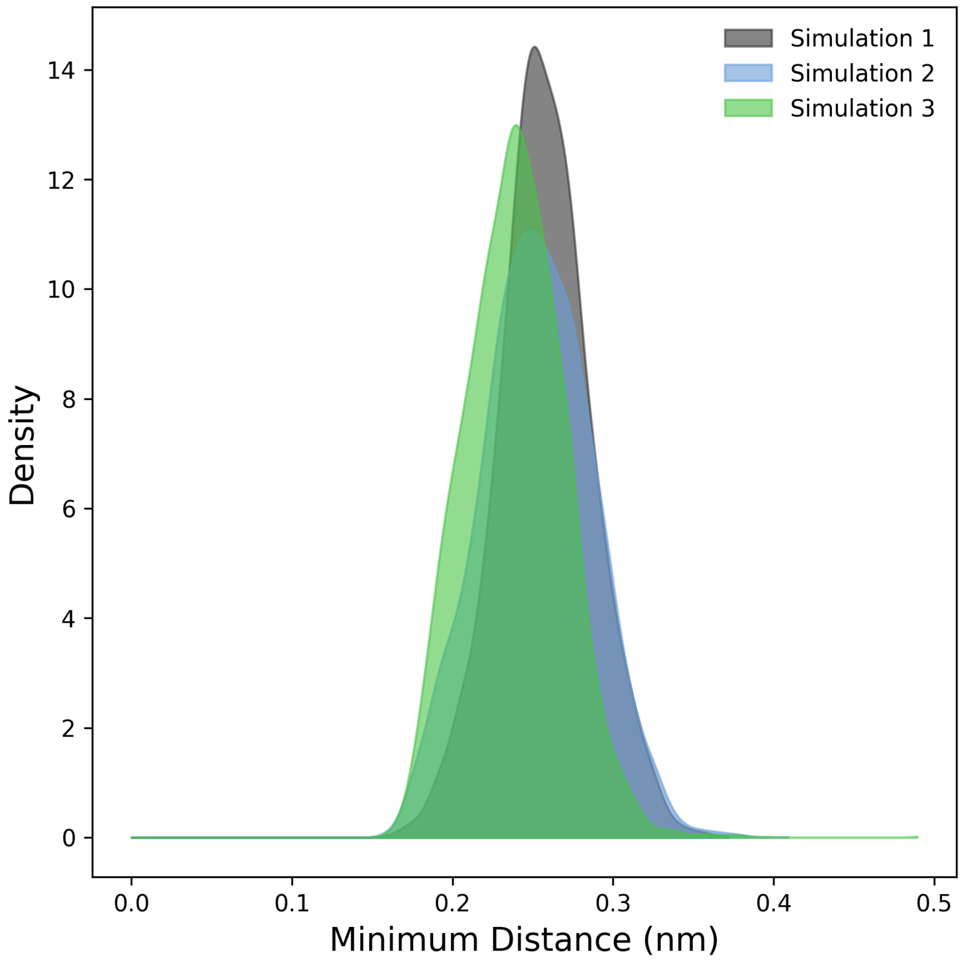

Interpretation guidance: This plot illustrates the density distribution derived from the temporal profile of the minimal distance between protein and ligand. Peaks in the distribution signify regions where the minimal distances occur more frequently, indicating preferred interaction distances. Broader peaks suggest a more variable and heterogeneous range of distances, while sharper, well-defined peaks reflect stable and consistent interaction proximities throughout the simulation.

12.2 Complete code

```
import numpy as np
import matplotlib.pyplot as plt
from scipy.stats import gaussian_kde
import os
```

```
def read_distance(file):
```

```
"""
Reads minimum distance data from a .xvg file.
Skips header lines and extracts time (in ns) and distance values.

Parameters:
-----------
file : str
    Path to the .xvg file

```

(continues on next page)

(continued from previous page)

```
    Returns:
    --------
    times : np.ndarray
        Array of time points in nanoseconds.
    distances : np.ndarray
        Array of minimum distances at each time point.
    """
    try:
        times = []
        distances = []
        with open(file, 'r') as f:
            for line in f:
                # Skip comments and empty lines
                if line.startswith(('#', '@', ';')) or line.strip() == '':
                    continue
                try:
                    values = line.split()
                    if len(values) >= 2:
                        time_ps, dist_val = map(float, values[:2])
                        times.append(time_ps / 1000.0)  # Convert from ps to ns
                        distances.append(dist_val)
                except ValueError:
                    # Ignore lines that cannot be converted to floats
                    continue

        # Check if data was read correctly
        if len(times) == 0 or len(distances) == 0:
            raise ValueError(f"File {file} does not contain valid data.")

        return np.array(times), np.array(distances)

    except Exception as e:
        print(f"Error reading file {file}: {e}")
        return None, None
```

```
def check_simulation_times(*time_arrays):
```

```
    """
    Checks if all simulation time arrays are consistent (equal).
    Raises an error if times do not match to avoid misaligned averaging.
    """
    for i in range(1, len(time_arrays)):
        if not np.allclose(time_arrays[0], time_arrays[i]):
            raise ValueError(f"Simulation times do not match between file 1 and file
↪{i+1}")
```

```
def plot_distance(results, output_folder, config=None):
```

```
    """
    Plots the mean minimum distance over time with shaded std deviation.

    Parameters:
    -----------
    results : list of tuples
        Each tuple is (time_array, mean_distance, std_distance) for one simulation␣
```

(continues on next page)

(continued from previous page)

```
↪group.
    output_folder : str
        Folder path to save the plots.
    config : dict, optional
        Plot configuration dictionary (colors, labels, axis labels, etc.)
    """
    plt.figure(figsize=config.get('figsize', (9, 6)))

    # Plot mean ± std for each simulation group
    for idx, (time, mean, std) in enumerate(results):
        label = config['labels'][idx] if config and 'labels' in config else f
↪'Simulation {idx+1}'
        color = config['colors'][idx] if config and 'colors' in config else None
        alpha = config.get('alpha', 0.2)
        plt.plot(time, mean, label=label, color=color, linewidth=2)
        plt.fill_between(time, mean - std, mean + std, color=color, alpha=alpha)

    plt.xlabel(config.get('xlabel', 'Time (ns)'), fontsize=config.get('label_fontsize',
↪ 12))
    plt.ylabel(config.get('ylabel', 'Minimum Distance (nm)'), fontsize=config.get(
↪'label_fontsize', 12))
    plt.legend(frameon=False, loc='upper right', fontsize=10)
    plt.tick_params(axis='both', which='major', labelsize=10)

    # Dynamically set x and y axis limits to start at 0 and cover data range with␣
↪margin
    max_time = max([np.max(time) for time, _, _ in results])
    max_val = max([np.max(mean + std) for _, mean, std in results])
    plt.xlim(0, max_time)
    plt.ylim(0, max_val * 1.05)  # Add 5% margin above max

    plt.tight_layout()

    # Create output folder if it doesn't exist
    os.makedirs(output_folder, exist_ok=True)

    # Save plots in TIFF and PNG formats
    plt.savefig(os.path.join(output_folder, 'distance_plot.tiff'), dpi=300)
    plt.savefig(os.path.join(output_folder, 'distance_plot.png'), dpi=300)

    plt.show()
```

```
def plot_distance_density(results, output_folder, config=None):
```

```
    """
    Plots kernel density estimates of the minimum distance distributions.

    Parameters:
    -----------
    results : list of tuples
        Each tuple is (time_array, mean_distance, std_distance).
        Only the mean_distance array is used here for density estimation.
    output_folder : str
        Folder path to save the density plots.
    config : dict, optional
        Plot configuration dictionary.
```

(continues on next page)

(continued from previous page)

```
"""
plt.figure(figsize=config.get('figsize', (6, 6)))

# Plot KDE for each simulation group's mean distance values
for idx, (_, mean, _) in enumerate(results):
    kde = gaussian_kde(mean)
    x_vals = np.linspace(0, max(mean), 1000)
    label = config['labels'][idx] if config and 'labels' in config else f
↪'Simulation {idx+1}'
    color = config['colors'][idx] if config and 'colors' in config else None
    alpha = config.get('alpha', 0.5)
    plt.fill_between(x_vals, kde(x_vals), color=color, alpha=alpha, label=label)

plt.xlabel(config.get('xlabel', 'Minimum Distance (nm)'), fontsize=config.get(
↪'label_fontsize', 12))
plt.ylabel(config.get('ylabel', 'Density'), fontsize=config.get('label_fontsize',␣
↪12))
plt.legend(frameon=False, loc='upper right', fontsize=10)
plt.tight_layout()

# Save density plots
plt.savefig(os.path.join(output_folder, 'distance_density.tiff'), dpi=300)
plt.savefig(os.path.join(output_folder, 'distance_density.png'), dpi=300)

plt.show()
```

```
def distance_analysis(output_folder, *simulation_file_groups, distance_config=None,
↪ density_config=None):
```

```
"""
Main function to process multiple simulation groups and generate minimum distance␣
↪plots.

Parameters:
-----------
output_folder : str
    Directory where the plots will be saved.
*simulation_file_groups : list of lists
    Each element is a list of file paths for replicates of a simulation group.
distance_config : dict, optional
    Configuration for the distance vs time plot.
density_config : dict, optional
    Configuration for the density plot.
"""
def process_group(file_paths):
    times = []
    distances = []
    for file in file_paths:
        time, dist_val = read_distance(file)
        times.append(time)
        distances.append(dist_val)
    check_simulation_times(*times)
    mean_distance = np.mean(distances, axis=0)
    std_distance = np.std(distances, axis=0)
    return times[0], mean_distance, std_distance
```

(continues on next page)

(continued from previous page)

```
results = []
for group in simulation_file_groups:
    if group:
        time, mean, std = process_group(group)
        results.append((time, mean, std))

if len(results) >= 1:
    plot_distance(results, output_folder, config=distance_config)
    plot_distance_density(results, output_folder, config=density_config)
else:
    raise ValueError("At least one simulation group is required.")
```

13 Protein–Ligand Hydrophobic Contacts

13.1 Overview

DynamiSpectra offers a robust and versatile analytical framework designed to quantify and monitor hydrophobic contacts between protein and ligand molecules throughout molecular dynamics simulations. Utilizing input data in standardized .xvg file format, this module enables researchers to characterize the extent and dynamics of hydrophobic interactions, which are fundamental for molecular recognition, binding affinity, and complex stability.

This analysis provides detailed insights into the temporal evolution of hydrophobic contacts, allowing identification of transient contacts, persistent hydrophobic patches, or disruption of interactions. By incorporating data from multiple simulation replicates, *DynamiSpectra* computes averaged contact profiles with corresponding variability measures, such as standard deviation, facilitating statistically meaningful interpretation. Moreover, the software supports analysis of individual simulation replicas, enabling flexible usage when replicate data are unavailable or unnecessary.

Command line in GROMACS to generate .xvg files for the analysis:

```
gmx select -f Simulation.xtc -s Simulation.tpr -n index.ndx -select 'group "LIG"␣
↪and within 0.6 of (resname ALA VAL LEU ILE PHE MET and group "Protein")' -os␣
↪Hydrophobic_contacts.xvg
```

```
def hydrophobic_analysis(output_folder, *simulation_file_groups, contact_
↪config=None, density_config=None)
```

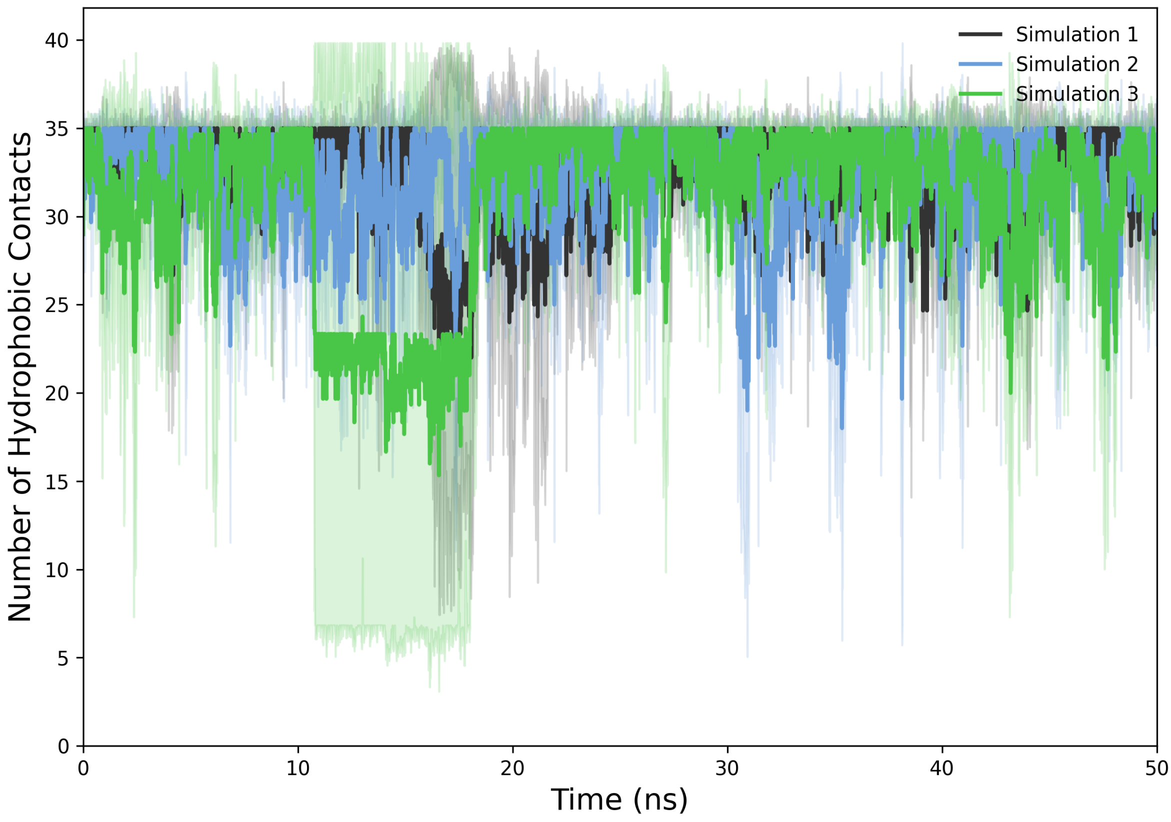

Interpretation guidance: The resulting plots illustrate the temporal progression of hydrophobic contacts between protein and ligand. A consistently high number of contacts indicates stable hydrophobic interactions and sustained binding affinity, whereas notable decreases or fluctuations may reflect conformational changes, disruption of hydrophobic patches, or transient binding events.

```
def plot_contact_density(results, output_folder, config=None)
```

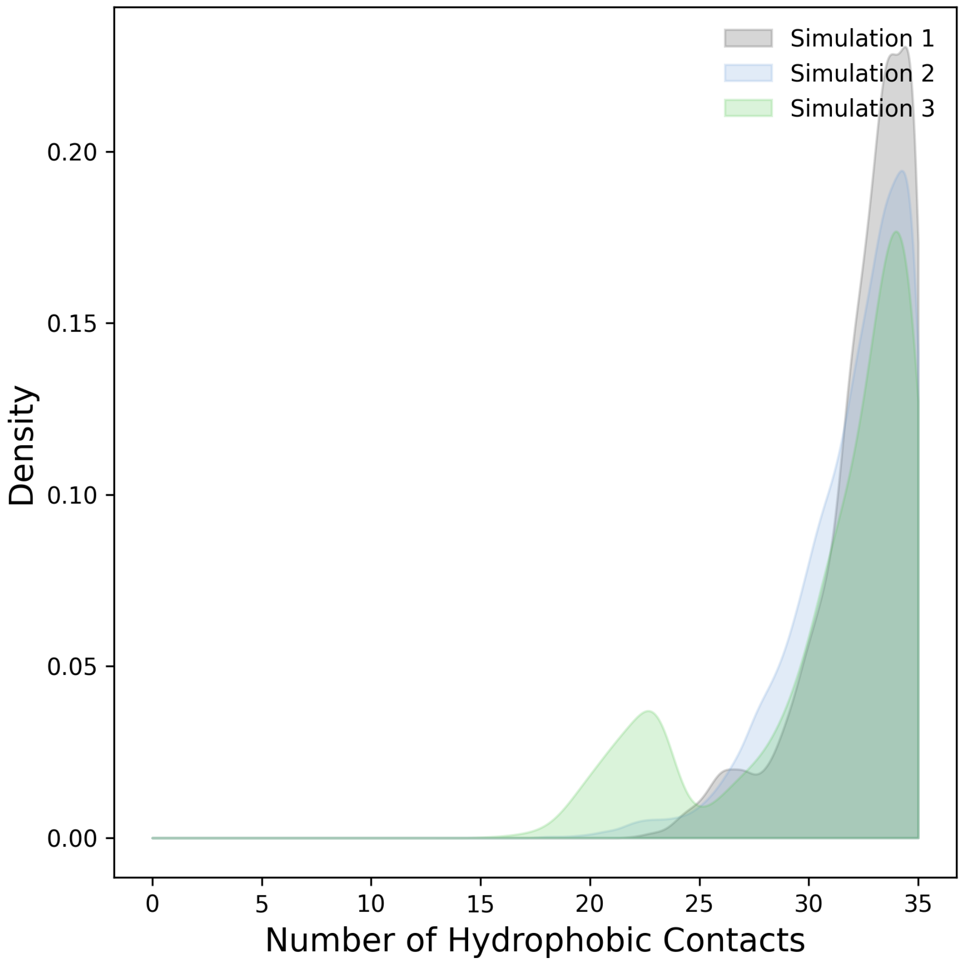

Interpretation guidance: This plot represents the density distribution derived from the temporal profile of minimal distances between protein and ligand. Peaks in the distribution indicate preferred interaction distances that occur more frequently during the simulation. Broader peaks suggest a more heterogeneous and variable range of distances, whereas sharper and well-defined peaks reflect stable and consistent spatial proximities maintained throughout the simulation.

13.2 Complete code

```
import numpy as np
import matplotlib.pyplot as plt
import os
from scipy.stats import gaussian_kde
```

```
def read_contacts(file):
```

```
"""
Reads a .xvg file containing hydrophobic contacts over time.

Parameters:
-----------
file : str
    Path to the .xvg file.

Returns:
```

(continues on next page)

(continued from previous page)

```
--------
times : np.ndarray
    Time values (converted from ps to ns).
contacts : np.ndarray
    Number of hydrophobic contacts at each time point.
"""
times, contacts = [], []
try:
    with open(file, 'r') as f:
        for line in f:
            # Skip comment, metadata, and empty lines
            if line.startswith(('#', '@', ';')) or line.strip() == '':
                continue
            values = line.split()
            if len(values) >= 2:
                # Extract time and contact number; convert ps to ns
                time_ps, contact = map(float, values[:2])
                times.append(time_ps / 1000.0)
                contacts.append(contact)
    # Raise an error if no valid data was read
    if not times or not contacts:
        raise ValueError(f"No valid data in file: {file}")
    return np.array(times), np.array(contacts)
except Exception as e:
    print(f"Error reading file {file}: {e}")
    return None, None
```

```
def check_simulation_times(*time_arrays):
```

```
"""
Checks if all time arrays are consistent between replicas.
Raises an error if not.
"""
for i in range(1, len(time_arrays)):
    if not np.allclose(time_arrays[0], time_arrays[i]):
        raise ValueError("Time arrays do not match between simulations")
```

```
def plot_contacts(results, output_folder, config=None):
```

```
"""
Generates a line plot of hydrophobic contacts over time with standard deviation␣
↪shading.

Parameters:
-----------
results : list of tuples
    Each tuple contains (time, mean_contacts, std_contacts) for a simulation group.
output_folder : str
    Path to save the output plots.
config : dict
    Plot customization options.
"""
if config is None:
    config = {}
colors = config.get('colors', None)
```

(continues on next page)

(continued from previous page)

```
labels = config.get('labels', None)
figsize = config.get('figsize', (9, 6))
alpha = config.get('alpha', 0.2)
label_fontsize = config.get('label_fontsize', 12)
tick_fontsize = config.get('tick_fontsize', 10)
linewidth = config.get('linewidth', 2)

plt.figure(figsize=figsize)

# Plot each simulation with shading for std deviation
for idx, (time, mean, std) in enumerate(results):
    color = colors[idx] if colors and idx < len(colors) else None
    label = labels[idx] if labels and idx < len(labels) else f"Simulation {idx+1}"
    plt.plot(time, mean, label=label, color=color, linewidth=linewidth)
    plt.fill_between(time, mean - std, mean + std, color=color, alpha=alpha)

# Set labels and appearance
plt.xlabel(config.get('xlabel', 'Time (ns)'), fontsize=label_fontsize)
plt.ylabel(config.get('ylabel', 'Hydrophobic Contacts'), fontsize=label_fontsize)
plt.legend(frameon=False, loc='upper right', fontsize=tick_fontsize)
plt.tick_params(axis='both', labelsize=tick_fontsize)

# Set x and y limits automatically
plt.xlim(0, max([np.max(t) for t, _, _ in results]))
plt.ylim(0, max([np.max(m + s) for _, m, s in results]) * 1.05)

# Final layout and save
plt.tight_layout()
os.makedirs(output_folder, exist_ok=True)
plt.savefig(os.path.join(output_folder, 'hydrophobic_contacts_plot.tiff'), dpi=300)
plt.savefig(os.path.join(output_folder, 'hydrophobic_contacts_plot.png'), dpi=300)
plt.show()
```

```
def plot_contact_density(results, output_folder, config=None):
```

```
"""
Generates a kernel density estimate (KDE) plot of hydrophobic contact␣
↪distributions.

Parameters:
-----------
results : list of tuples
    Each tuple contains (time, mean_contacts, std_contacts) for a simulation group.
output_folder : str
    Path to save the density plot.
config : dict
    Plot customization options.
"""
if config is None:
    config = {}
colors = config.get('colors', None)
labels = config.get('labels', None)
figsize = config.get('figsize', (6, 6))
alpha = config.get('alpha', 0.3)
label_fontsize = config.get('label_fontsize', 12)
```

(continues on next page)

(continued from previous page)

```
plt.figure(figsize=figsize)

# Plot KDE for each simulation
for idx, (_, mean, _) in enumerate(results):
    kde = gaussian_kde(mean)
    x_vals = np.linspace(0, max(mean), 1000)
    color = colors[idx] if colors and idx < len(colors) else None
    label = labels[idx] if labels and idx < len(labels) else f"Simulation {idx+1}"
    plt.fill_between(x_vals, kde(x_vals), color=color, alpha=alpha, label=label)

# Set labels and appearance
plt.xlabel(config.get('xlabel', 'Number of Contacts'), fontsize=label_fontsize)
plt.ylabel(config.get('ylabel', 'Density'), fontsize=label_fontsize)
plt.legend(frameon=False, loc='upper right')
plt.tight_layout()

# Save the plot
plt.savefig(os.path.join(output_folder, 'hydrophobic_contacts_density.tiff'),␣
↪dpi=300)
plt.savefig(os.path.join(output_folder, 'hydrophobic_contacts_density.png'),␣
↪dpi=300)
plt.show()
```

```
def hydrophobic_analysis(output_folder, *simulation_file_groups, contact_
↪config=None, density_config=None):
```

```
"""
Main function to perform hydrophobic contact analysis.

Parameters:
-----------
output_folder : str
    Folder where output plots will be saved.
*simulation_file_groups : list of lists
    Each list should contain .xvg paths for replicas of one simulation group.
contact_config : dict
    Optional customization for contact vs. time plot.
density_config : dict
    Optional customization for density (KDE) plot.
"""
def process_group(file_paths):
    """
    Reads and processes all replicas in a simulation group.
    Returns time, mean, and standard deviation arrays.
    """
    times, contacts = [], []
    for file in file_paths:
        time, contact = read_contacts(file)
        if time is not None and contact is not None:
            times.append(time)
            contacts.append(contact)
    check_simulation_times(*times)
    mean_contacts = np.mean(contacts, axis=0)
    std_contacts = np.std(contacts, axis=0)
    return times[0], mean_contacts, std_contacts
```

(continues on next page)

(continued from previous page)

```
results = []
for group in simulation_file_groups:
    if group:
        time, mean, std = process_group(group)
        results.append((time, mean, std))

if results:
    plot_contacts(results, output_folder, config=contact_config)
    plot_contact_density(results, output_folder, config=density_config)
else:
    raise ValueError("At least one simulation group is required.")
```

14 Inter-residue Distance Matrix

14.1 Overview

DynamiSpectra provides a detailed analytical tool to calculate and visualize inter-residue distance matrices within molecular systems during molecular dynamics simulations, using input data in .xpm file formats. This analysis allows researchers to examine the spatial relationships and dynamic proximities between residues of a single molecular entity, such as a protein, thereby offering insights into structural conformations and intra-molecular interactions.

The inter-residue distance matrix represents pairwise distances between residues across the entire molecular chain, enabling the identification of stable contacts, flexible regions, and conformational rearrangements.

Command line in GROMACS to generate .xvg files for the analysis:

```
gmx mdmat -f Simulation.xtc -s Simulation.tpr -mean Simulation.xpm -no Simulation.
↪xvg
```

```
def distance_matrix_analysis(xpm_file_path, output_path=None, plot=True,␣
↪config=None)
```

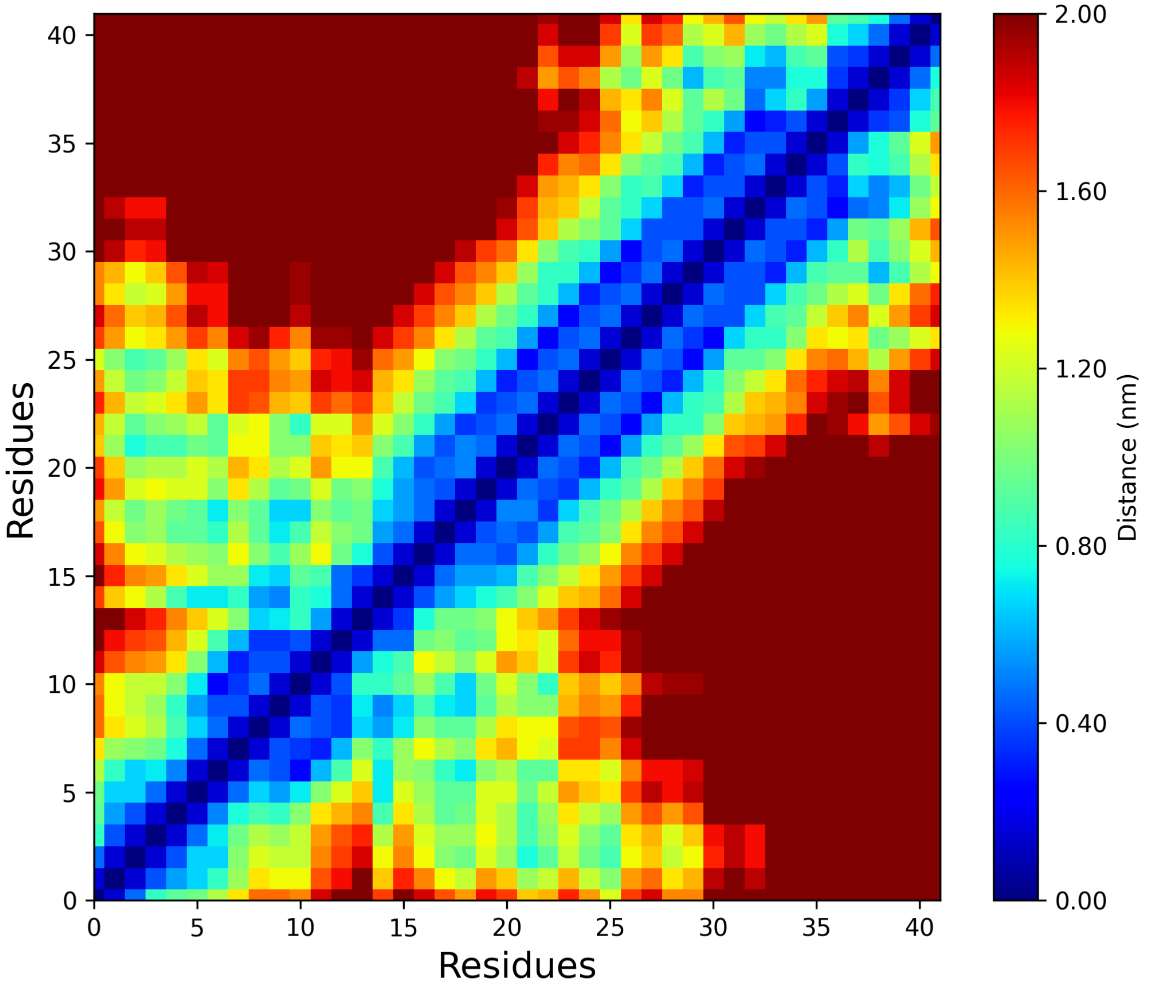

Interpretation guidance: The resulting distance matrix is represented as a colored map, where darker regions correspond to larger inter-residue distances and lighter regions indicate closer proximities. This visualization enables the identification of tightly packed or interacting residue pairs as lighter areas, while more distant residues appear as darker zones.

14.2 Complete code

```
import numpy as np
import matplotlib.pyplot as plt
import os
from matplotlib import cm
```

```
def read_xpm(file_path):
```

```
"""
Reads an XPM file and converts it to a numerical matrix.

Parameters:
-----------
file_path : str
    Path to the XPM file.

Returns:
```

(continues on next page)

(continued from previous page)

```
---------
matrix : np.ndarray
    Matrix with numeric values representing the image.
"""
with open(file_path, 'r') as f:
    lines = f.readlines()

# Extract only lines with pixel data
lines = [l.strip() for l in lines if l.strip().startswith('"')]
header_line = lines[0].strip().strip('",')
width, height, ncolors, chars_per_pixel = map(int, header_line.split())

# Build a color map from symbols to integers
color_map = {}
for i in range(1, ncolors + 1):
    line = lines[i].strip().strip('",')
    symbol = line[:chars_per_pixel]
    color_map[symbol] = i - 1

# Create matrix from symbols
matrix = []
for line in lines[ncolors + 1:]:
    line = line.strip().strip('",')
    row = [color_map[line[i:i+chars_per_pixel]] for i in range(0, len(line), chars_
↪per_pixel)]
    matrix.append(row)

return np.array(matrix)
```

```
def plot_distance_matrix(matrix, save_path=None,
                         xlabel='Residues', ylabel='Residues',
                         label_fontsize=12,
                         title='', title_fontsize=14,
                         colorbar_label='Distance (nm)', max_distance=1.5,
                         cmap='jet'):
```

```
"""
Plots a normalized distance matrix with a custom colormap.

Parameters:
-----------
matrix : np.ndarray
    Input distance matrix.
save_path : str
    Path to save the plot (without extension).
xlabel, ylabel : str
    Labels for x and y axes.
label_fontsize : int
    Font size for axis labels.
title : str
    Title of the plot.
title_fontsize : int
    Font size of the title.
colorbar_label : str
    Label for the colorbar.
max_distance : float
```

(continues on next page)

(continued from previous page)

```
    Max distance used to normalize the matrix.
cmap : str
    Colormap used for the plot.
"""
# Normalize the matrix by max value
matrix_norm = matrix / np.max(matrix) * max_distance

# Create the plot
plt.figure(figsize=(7, 6))
plt.imshow(np.fliplr(matrix_norm), cmap=cmap, origin='lower', aspect='auto')
plt.gca().invert_xaxis()

# Set axis limits and ticks
plt.xlim(0, matrix.shape[1]-1)
plt.ylim(0, matrix.shape[0]-1)
plt.xticks(np.arange(0, matrix.shape[1], step=5))
plt.yticks(np.arange(0, matrix.shape[0], step=5))

# Add colorbar
cbar = plt.colorbar()
cbar.set_label(colorbar_label)
cbar.set_ticks(np.linspace(0, max_distance, 6))
cbar.set_ticklabels([f"{x:.2f}" for x in np.linspace(0, max_distance, 6)])

# Labels and title
plt.xlabel(xlabel, fontsize=label_fontsize)
plt.ylabel(ylabel, fontsize=label_fontsize)
plt.title(title, fontsize=title_fontsize)

# Adjust layout
plt.tight_layout()

# Save plot if path provided
if save_path:
    base, _ = os.path.splitext(save_path)
    plt.savefig(f"{base}.png", dpi=300)
    plt.savefig(f"{base}.tiff", dpi=300)
    print(f"Plots saved as {base}.png and {base}.tiff")

# Show the plot
plt.show()
```

```
def distance_matrix_analysis(xpm_file_path, output_path=None, plot=True,␣
↪config=None):
```

```
"""
Main function to perform distance matrix analysis and plotting.

Parameters:
-----------
xpm_file_path : str
    Path to the input XPM file.
output_path : str
    Path (without extension) to save output plot.
plot : bool
    Whether to display the plot.
```

(continues on next page)

(continued from previous page)

```
config : dict (optional)
    Plot customization dictionary with keys:
        - xlabel, ylabel
        - label_fontsize
        - title, title_fontsize
        - colorbar_label
        - max_distance
        - cmap
"""
if config is None:
    config = {}

matrix = read_xpm(xpm_file_path)

if plot:
    plot_distance_matrix(
        matrix,
        save_path=output_path,
        xlabel=config.get('xlabel', 'Residues'),
        ylabel=config.get('ylabel', 'Residues'),
        label_fontsize=config.get('label_fontsize', 12),
        title=config.get('title', ''),
        title_fontsize=config.get('title_fontsize', 14),
        colorbar_label=config.get('colorbar_label', 'Distance (nm)'),
        max_distance=config.get('max_distance', 1.5),
        cmap=config.get('cmap', 'jet')
    )

return matrix
```

15 Phi and Psi Angles

15.1 Overview

DynamiSpectra provides a robust and flexible analytical framework for analyzing backbone dihedral angles φ (phi) and ψ (psi) of proteins throughout molecular dynamics simulations. Utilizing .xvg files generated from simulation outputs, this module enables detailed assessment of protein conformational preferences and structural dynamics by capturing the distribution and evolution of φ and ψ angles over time.

This analysis offers comprehensive insights into predominant conformations, conformational transitions, and rare structural states. The graphical outputs generated by *DynamiSpectra* facilitate clear visualization of protein backbone angular patterns, supporting both qualitative and quantitative interpretation of protein structural dynamics.

Command line in GROMACS to generate .xvg files for the analysis:

```
gmx rama -s Simulation.tpr -f Simulation.xtc -o rama_simulation.xvg
```

Note: *DynamiSpectra* generates individual phi and psi density plots for each simulation replica, as well as a combined density plot using data concatenated from all replicas. The concatenated density plot provides a comprehensive overview of the conformational space sampled across simulations, enabling identification of predominant structural states and improving statistical robustness compared to analyzing replicas separately.

```
def phipsi_analysis(output_folder, *simulation_file_groups, phipsi_config=None,␣
↪residue_name=None)
```

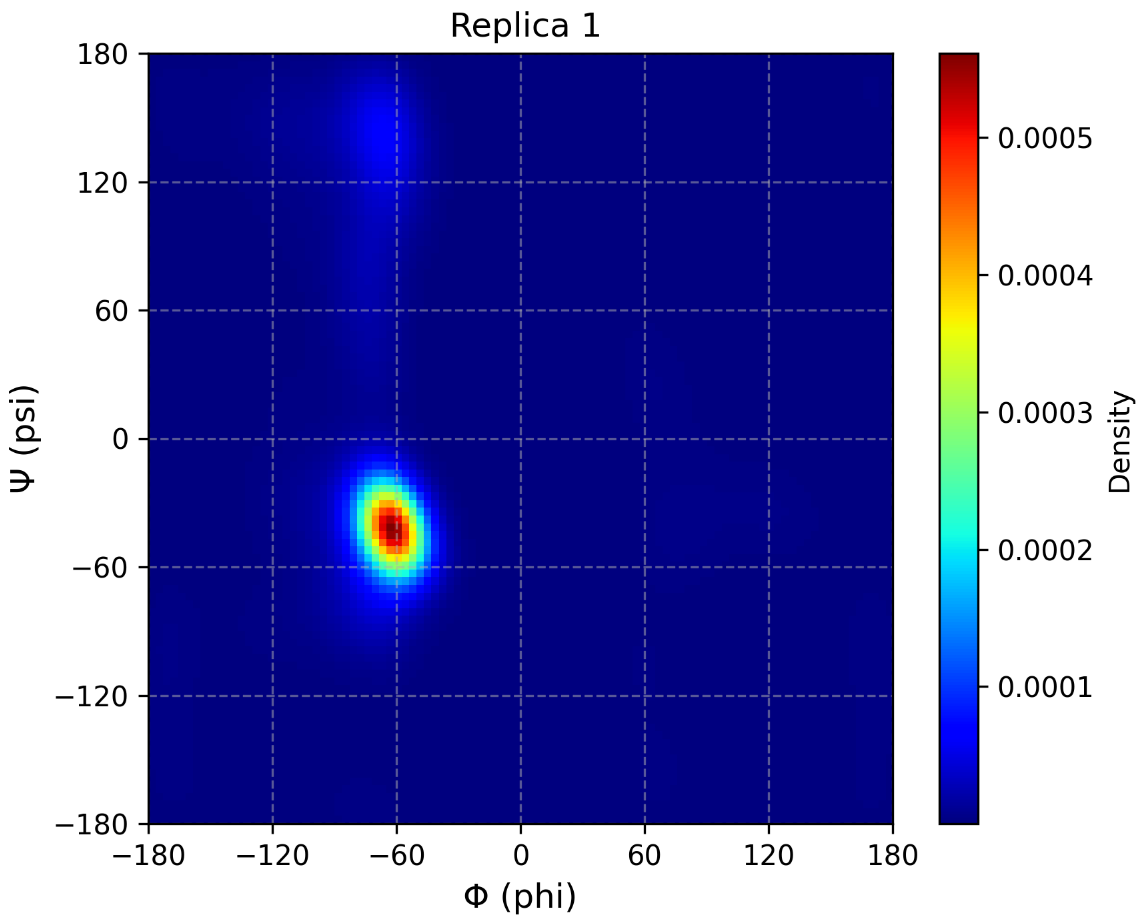

Interpretation guidance: This plot presents a color density map of the sampled backbone dihedral angles (phi and psi) of the protein or residue throughout the molecular dynamics simulation. Regions with stronger (more intense) colors indicate higher density, meaning the protein or residue spent more time adopting those angular conformations, reflecting preferred and stable states. Conversely, areas with weaker colors represent lower density regions with less frequent or transient conformations. This visualization provides valuable insights into the conformational flexibility and dynamic behavior of the protein during the simulation.

15.2 Complete code

```
import os
import numpy as np
import matplotlib.pyplot as plt
from scipy.stats import gaussian_kde
```

```
def read_rama(file_path, residue_name=None):
```

```
phi = []
psi = []
try:
    with open(file_path, 'r') as f:
        for line in f:
            if line.startswith(('#', '@')) or line.strip() == '':
                continue
            parts = line.strip().split()
```

(continues on next page)

(continued from previous page)

```
            if len(parts) >= 3:
                try:
                    phi_val = float(parts[0])
                    psi_val = float(parts[1])
                    res_name = parts[2]
                    if residue_name is None or res_name == residue_name:
                        phi.append(phi_val)
                        psi.append(psi_val)
                except ValueError:
                    continue
    return np.array(phi), np.array(psi)
except Exception as e:
    print(f"Failed to read {file_path}: {e}")
    return np.array([]), np.array([])
```

```
def kde_2d(phi, psi, grid_size=100, bandwidth=None):
```

```
values = np.vstack([phi, psi])
kde = gaussian_kde(values, bw_method=bandwidth)
x_grid = np.linspace(-180, 180, grid_size)
y_grid = np.linspace(-180, 180, grid_size)
X, Y = np.meshgrid(x_grid, y_grid)
grid_coords = np.vstack([X.ravel(), Y.ravel()])
Z = kde(grid_coords).reshape(grid_size, grid_size)
return X, Y, Z
```

```
def plot_phipsi_kde(results, output_folder, config=None, residue_name=None):
```

```
if config is None:
    config = {}

grid_size = config.get('grid_size', 100)
cmap = config.get('cmap', 'jet')
alpha = config.get('alpha', 1)
figsize_subplot = config.get('figsize_subplot', (6, 5))
label_fontsize = config.get('label_fontsize', 12)
group_names = config.get('group_names', [])

num_groups = len(results)

fig, axes = plt.subplots(1, num_groups, figsize=(figsize_subplot[0]*num_groups,␣
↪figsize_subplot[1]), squeeze=False)

for idx, (phis, psis) in enumerate(results):
    ax = axes[0, idx]
    X, Y, Z = kde_2d(phis, psis, grid_size=grid_size)

    im = ax.imshow(
        Z,
        extent=[-180, 180, -180, 180],
        origin='lower',
        cmap=cmap,
        alpha=alpha,
        aspect='auto'
    )
```

(continues on next page)

(continued from previous page)

```
    title = group_names[idx] if idx < len(group_names) else f'Simulation {idx+1}'
    ax.set_title(title)
    ax.set_xlabel('Φ (phi)', fontsize=label_fontsize)
    ax.set_ylabel('Ψ (psi)', fontsize=label_fontsize)
    ax.set_xlim(-180, 180)
    ax.set_ylim(-180, 180)
    ax.set_xticks(np.arange(-180, 181, 60))
    ax.set_yticks(np.arange(-180, 181, 60))
    ax.grid(True, linestyle='--', alpha=0.5)
    fig.colorbar(im, ax=ax, label='Density')

plt.tight_layout()
os.makedirs(output_folder, exist_ok=True)
file_prefix = residue_name.replace('-', '_') if residue_name else 'all'
plt.savefig(os.path.join(output_folder, f'phipsi_kde_subplots_{file_prefix}.tiff'),
↪ dpi=300)
plt.savefig(os.path.join(output_folder, f'phipsi_kde_subplots_{file_prefix}.png'),␣
↪dpi=300)
plt.show()
```

```
def plot_single_phipsi_kde(phi, psi, output_folder, config=None, label='Replica'):
```

```
if config is None:
    config = {}
grid_size = config.get('grid_size', 100)
cmap = config.get('cmap', 'jet')
alpha = config.get('alpha', 1)
figsize = config.get('figsize_subplot', (6, 5))
label_fontsize = config.get('label_fontsize', 12)

X, Y, Z = kde_2d(phi, psi, grid_size=grid_size)

plt.figure(figsize=figsize)
plt.imshow(
    Z,
    extent=[-180, 180, -180, 180],
    origin='lower',
    cmap=cmap,
    alpha=alpha,
    aspect='auto'
)
plt.title(label)
plt.xlabel('Φ (phi)', fontsize=label_fontsize)
plt.ylabel('Ψ (psi)', fontsize=label_fontsize)
plt.xlim(-180, 180)
plt.ylim(-180, 180)
plt.xticks(np.arange(-180, 181, 60))
plt.yticks(np.arange(-180, 181, 60))
plt.grid(True, linestyle='--', alpha=0.5)
plt.colorbar(label='Density')

os.makedirs(output_folder, exist_ok=True)
safe_label = label.replace(' ', '_').replace('-', '_')
plt.savefig(os.path.join(output_folder, f'phipsi_kde_{safe_label}.png'), dpi=300)
plt.savefig(os.path.join(output_folder, f'phipsi_kde_{safe_label}.tiff'), dpi=300)
```

(continues on next page)

(continued from previous page)

```
plt.show()
```

```
def phipsi_analysis(output_folder, *simulation_file_groups, phipsi_config=None,␣
↪residue_name=None):
```

```
results = []
group_names = []
if phipsi_config is None:
    phipsi_config = {}

if 'group_names' in phipsi_config:
    group_names = phipsi_config['group_names']

for idx, group in enumerate(simulation_file_groups):
    group_phi = []
    group_psi = []

    # Concatena todos os dados do grupo para o gráfico combinado
    for file in group:
        phi_vals, psi_vals = read_rama(file, residue_name=residue_name)
        group_phi.extend(phi_vals)
        group_psi.extend(psi_vals)
    results.append((np.array(group_phi), np.array(group_psi)))

    # Gera gráficos individuais para cada réplica e mostra no Jupyter
    for i, file in enumerate(group):
        phi_vals, psi_vals = read_rama(file, residue_name=residue_name)
        label = f"{group_names[idx] if idx < len(group_names) else f'Simulation
↪{idx+1}'} Replica {i+1}"
        plot_single_phipsi_kde(np.array(phi_vals), np.array(psi_vals), output_
↪folder,
                               config=phipsi_config, label=label)

if results:
    plot_phipsi_kde(results, output_folder, config=phipsi_config, residue_
↪name=residue_name)
else:
    raise ValueError("No valid rama.xvg data provided.")
```

16 Rotamers

16.1 Overview

DynamiSpectra provides a comprehensive framework for analyzing protein side chain rotameric states by examining the Chi1 and Chi2 dihedral angles during molecular dynamics simulations. Using input .xvg files, this module calculates circular means of these angles across multiple simulation replicas, allowing robust statistical analysis of side chain conformational dynamics.

The module generates three types of plots: a 2D kernel density estimate (KDE) to visualize preferred rotamer conformations, a dotplot showing mean Chi1 versus Chi2 angles over time, and histograms displaying the distribution of individual Chi1 and Chi2 angles. This enables detailed characterization of rotamer populations, transitions, and conformational preferences critical for understanding protein function and dynamics.

Command line in GROMACS to generate .xvg files for the analysis:

```
gmx angle -f Simulation.xtc -n index.ndx -type dihedral -ov Simulation.xvg
```

Note: The atoms that define the Chi1 and Chi2 dihedral angles must be specified in the index file (.ndx) used with the gmx angle command. Ensure that the correct atom quadruplets are selected for each angle to obtain accurate rotameric data.

```
def dihedral_kde_and_dotplot(output_folder, chi1_files, chi2_files, config=None,␣
↪time_window=None)
```

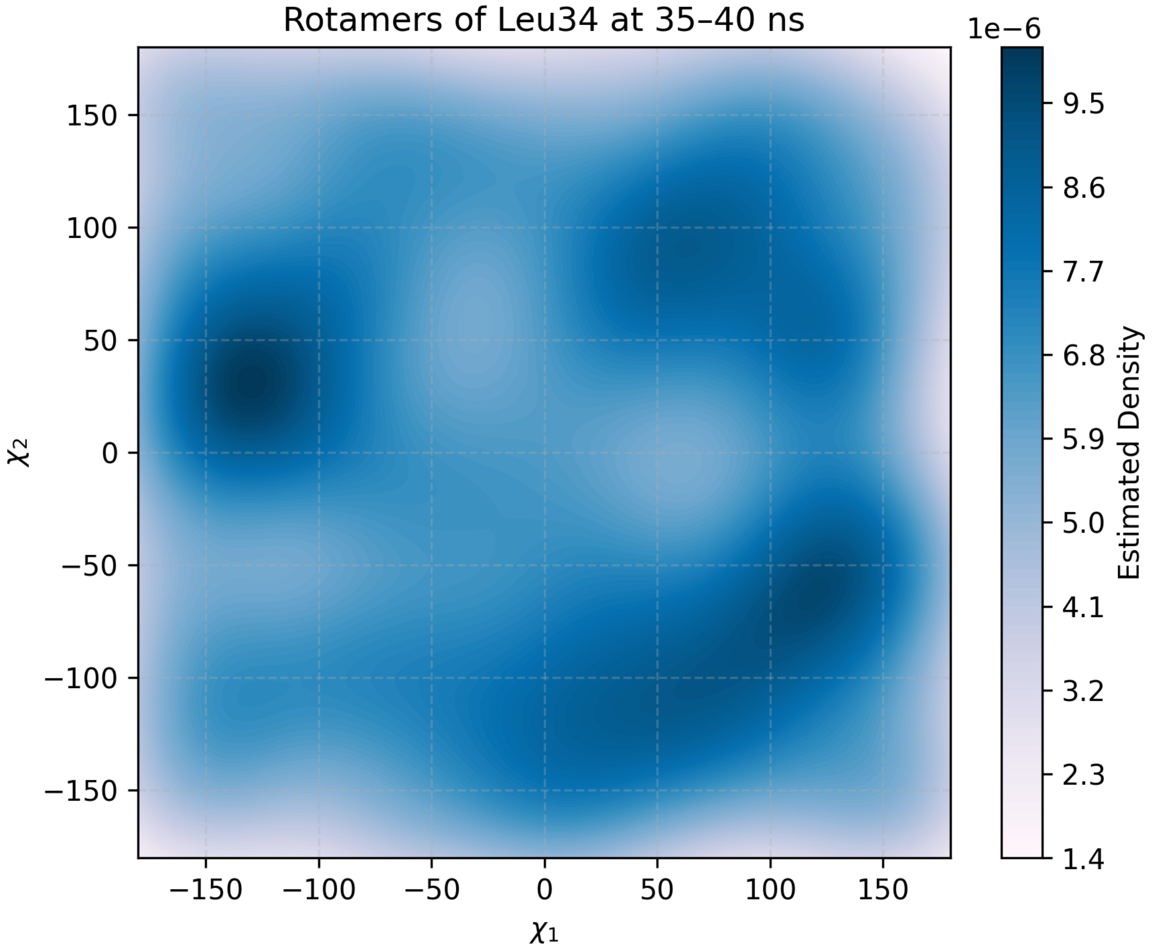

Interpretation guidence: This 2D kernel density estimate (KDE) plot shows the distribution of Chi1 and Chi2 side-chain dihedral angles over the simulation. Darker regions correspond to higher estimated densities, indicating that those angular combinations were more frequently sampled. These high-density zones reveal preferred rotameric states adopted by the residue, while lighter areas indicate less frequent conformations. This plot provides insight into side-chain conformational preferences and flexibility during the simulation.

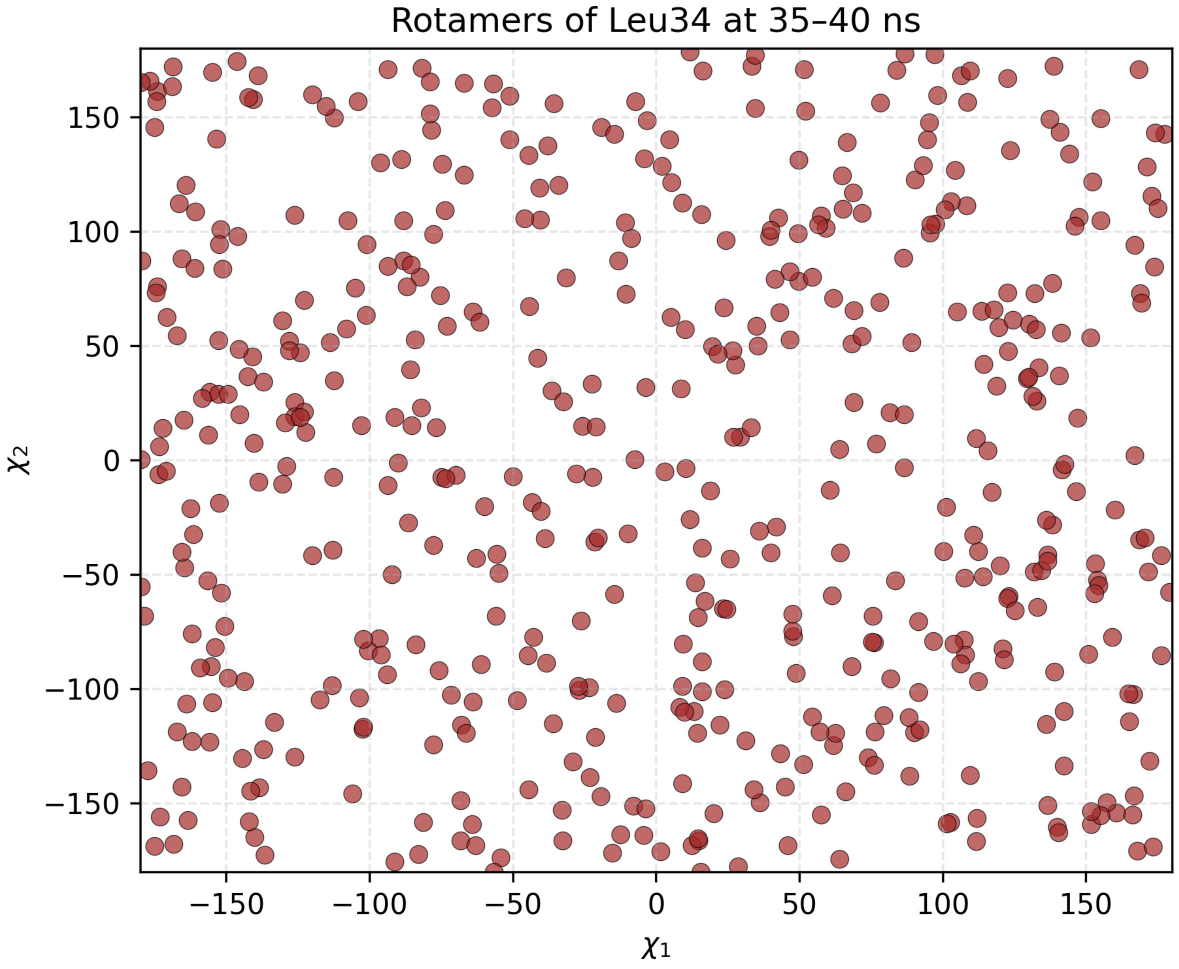

Interpretation guidence: This plot displays the mean Chi1 and Chi2 dihedral angles over the course of the simulation or within a defined time window. Each point represents the averaged side chain conformation across multiple replicas at a given time. Clusters or patterns in the plot indicate preferred rotameric states, while dispersion suggests conformational variability. This visualization is useful for identifying dominant side chain orientations and assessing the dynamic range of rotameric transitions within the selected period.

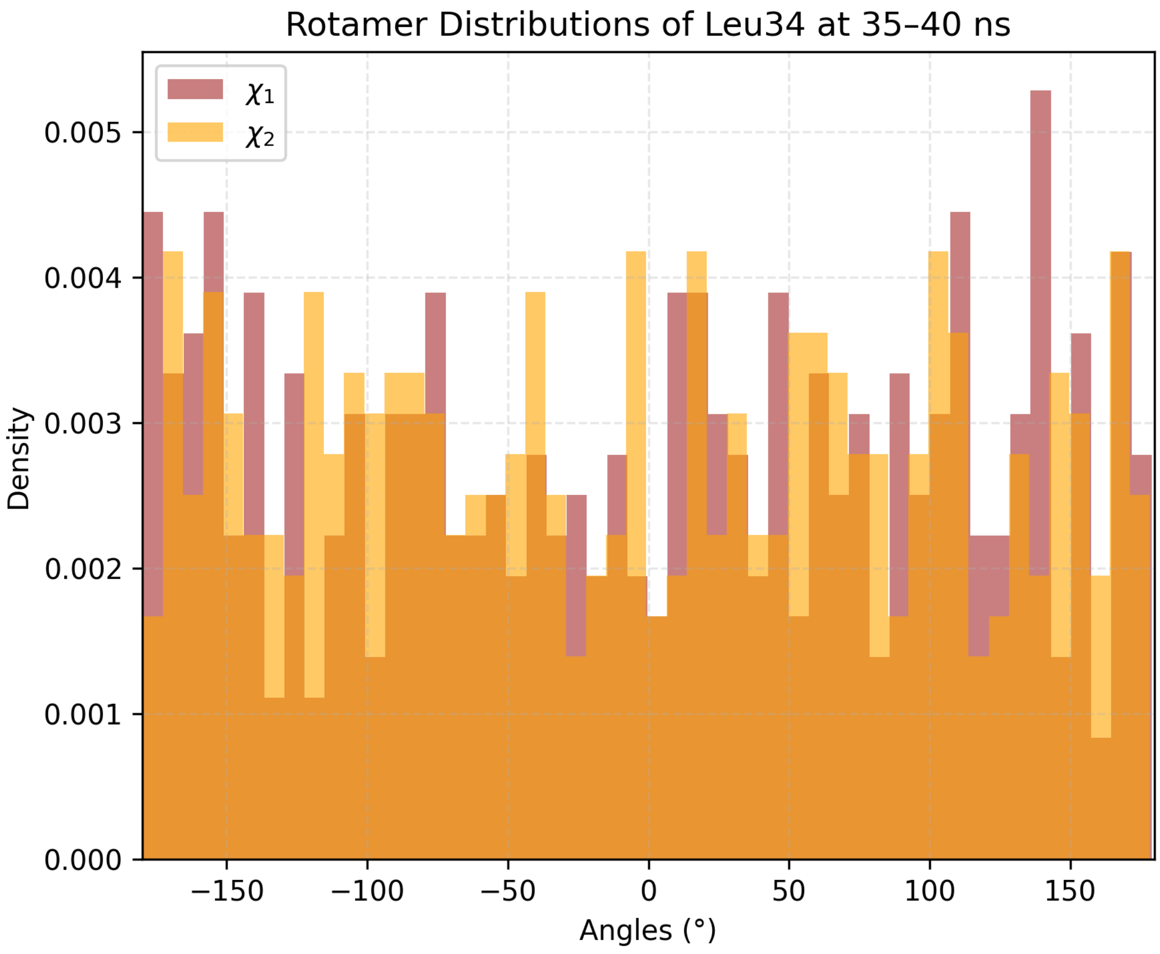

Interpretation guidance: This histogram depicts the distribution density of dihedral angles observed during the simulation or within a selected time window. Distinct peaks indicate preferred rotamer conformations, while broader or multiple peaks suggest increased conformational flexibility or transitions between states.

16.2 Complete code

```
import os
import numpy as np
import matplotlib.pyplot as plt
from scipy.stats import gaussian_kde
```

```
def read_angle_time_xvg(file_path):
```

```
"""
Reads a GROMACS .xvg file containing time and angle data.
Returns time and angle arrays.
"""
times = []
angles = []
with open(file_path, 'r') as f:
    for line in f:
        if line.startswith(('#', '@')) or line.strip() == '':
            continue
        try:
```

(continues on next page)

(continued from previous page)

```
            parts = line.strip().split()
            time = float(parts[0])
            angle = float(parts[1])
            times.append(time)
            angles.append(angle)
        except (IndexError, ValueError):
            continue
return np.array(times), np.array(angles)
```

```
def circular_mean_deg(angles_deg):
```

```
"""
Computes the circular mean of a set of angles (in degrees).
This is used to correctly handle periodic data such as dihedrals.
"""
angles_rad = np.deg2rad(angles_deg)
sin_sum = np.mean(np.sin(angles_rad), axis=0)
cos_sum = np.mean(np.cos(angles_rad), axis=0)
mean_angle_rad = np.arctan2(sin_sum, cos_sum)
mean_angle_deg = np.rad2deg(mean_angle_rad)
return mean_angle_deg
```

```
def mean_angles_across_replicas(file_list):
```

```
"""
Computes the circular mean angle at each time point across multiple replicas.
All trajectories are trimmed to the shortest one to ensure alignment.
"""
all_times = []
all_angles = []
for f in file_list:
    times, angles = read_angle_time_xvg(f)
    all_times.append(times)
    all_angles.append(angles)

min_len = min(len(t) for t in all_times)
all_times_cut = [t[:min_len] for t in all_times]
all_angles_cut = [a[:min_len] for a in all_angles]

common_times = all_times_cut[0]
all_angles_arr = np.array(all_angles_cut)  # shape: (replicates, timepoints)

mean_angles = circular_mean_deg(all_angles_arr)
return common_times, mean_angles
```

```
def dihedral_kde_and_dotplot(output_folder, chi1_files, chi2_files, config=None,␣
↪time_window=None):
```

```
"""
Generates KDE, dotplot, and histogram plots from Chi1 and Chi2 dihedral angles.
"""
cfg = {
    'figsize': (18, 5),
    'kde_title': 'Chi1 vs Chi2 (KDE)',
    'dot_title': 'Chi1 vs Chi2 (Dotplot)',
```

(continues on next page)

(continued from previous page)

```
    'hist_title': 'Distribution of Chi1 and Chi2 Angles',
    'xlabel': r'$\chi_1$',
    'ylabel': r'$\chi_2$',
    'hist_xlabel': 'Angle (degrees)',
    'hist_ylabel': 'Density',
    'hist_legend_labels': ['Chi1', 'Chi2'],
    'cmap': 'Oranges',
    'dot_color': 'brown',
    'dot_alpha': 0.7,
    'chi1_color': 'blue',
    'chi2_color': 'green',
    'colorbar_label': 'Estimated Density',
    'save_name': 'kde_dotplot_chi1_vs_chi2.png',
    'levels': 100,
    'bins': 50
}
if config:
    cfg.update(config)

# Read and compute mean angles from replicas
times1, chi1_mean = mean_angles_across_replicas(chi1_files)
times2, chi2_mean = mean_angles_across_replicas(chi2_files)

assert np.array_equal(times1, times2), "Chi1 and Chi2 replica times do not match!"
times = times1

# Filter by time window if defined
if time_window is not None:
    t_min, t_max = time_window
    idxs = np.where((times >= t_min) & (times <= t_max))[0]
    if len(idxs) == 0:
        print("No data in the defined time window!")
        return
    chi1_mean = chi1_mean[idxs]
    chi2_mean = chi2_mean[idxs]

# 2D KDE
values = np.vstack([chi1_mean, chi2_mean])
kernel = gaussian_kde(values, bw_method=0.3)
xgrid = np.linspace(-180, 180, 200)
ygrid = np.linspace(-180, 180, 200)
X, Y = np.meshgrid(xgrid, ygrid)
positions = np.vstack([X.ravel(), Y.ravel()])
Z = np.reshape(kernel(positions).T, X.shape)

# Plotting layout
fig, axs = plt.subplots(1, 3, figsize=cfg['figsize'])

# Panel 1: KDE Plot
kde = axs[0].contourf(X, Y, Z, levels=cfg['levels'], cmap=cfg['cmap'])
axs[0].set_title(cfg['kde_title'])
axs[0].set_xlabel(cfg['xlabel'])
axs[0].set_ylabel(cfg['ylabel'])
axs[0].set_xlim(-180, 180)
axs[0].set_ylim(-180, 180)
axs[0].grid(True, linestyle='--', alpha=0.3)
```

(continues on next page)

(continued from previous page)

```
cbar = fig.colorbar(kde, ax=axs[0])
cbar.set_label(cfg['colorbar_label'])

# Panel 2: Dotplot
axs[1].scatter(chi1_mean, chi2_mean, color=cfg['dot_color'], alpha=cfg['dot_alpha
↪'],
               edgecolors='k', linewidths=0.3, s=40)
axs[1].set_title(cfg['dot_title'])
axs[1].set_xlabel(cfg['xlabel'])
axs[1].set_ylabel(cfg['ylabel'])
axs[1].set_xlim(-180, 180)
axs[1].set_ylim(-180, 180)
axs[1].grid(True, linestyle='--', alpha=0.3)

# Panel 3: Histograms
axs[2].hist(chi1_mean, bins=cfg['bins'], alpha=0.6, label=cfg['hist_legend_labels
↪'][0],
            color=cfg['chi1_color'], density=True)
axs[2].hist(chi2_mean, bins=cfg['bins'], alpha=0.6, label=cfg['hist_legend_labels
↪'][1],
            color=cfg['chi2_color'], density=True)
axs[2].set_title(cfg['hist_title'])
axs[2].set_xlabel(cfg['hist_xlabel'])
axs[2].set_ylabel(cfg['hist_ylabel'])
axs[2].legend()
axs[2].grid(True, linestyle='--', alpha=0.3)
axs[2].set_xlim(-180, 180)

# Save figure
os.makedirs(output_folder, exist_ok=True)
save_path = os.path.join(output_folder, cfg['save_name'])
plt.tight_layout()
plt.savefig(save_path, dpi=300)
plt.show()
```

17 Ligand Density

17.1 Overview

DynamiSpectra provides a comprehensive and versatile analytical framework designed to quantify and monitor the spatial density distribution of ligand molecules within molecular dynamics simulations. Utilizing input data in standardized .xpm file format, this module enables researchers to characterize ligand localization, aggregation, and dynamic behavior within the simulated environment.

This analysis offers detailed insights into the spatial distribution of ligand density, facilitating the identification of preferential binding regions, ligand clustering, or dispersal events. The graphical outputs generated by *DynamiSpectra* enable straightforward visualization of ligand density patterns throughout the simulation, supporting qualitative and quantitative interpretation of ligand behavior.

Command line in GROMACS to generate .xvg files for the analysis:

```
gmx densmap -s Simulation.tpr -f Simulation.xtc -n index.ndx -o map.xpm -aver z
```

Explanation of Axes:

The generated density map is a 2D projection of ligand atomic positions onto the XY plane of the simulation box. This projection is done by averaging coordinates along the Z-axis over the trajectory frames.

- **X axis:** corresponds to the simulation box's X spatial coordinate (in nanometers)
- **Y axis:** corresponds to the simulation box's Y spatial coordinate (in nanometers)

This means that the labels "X" and "Y" on the plot directly represent physical coordinates in real space, consistent with the standard Cartesian coordinate system used in molecular dynamics.

```
def ligand_density_analysis(xpm_file_path, output_path=None, plot=True,
                            cmap='inferno', xlabel='X', ylabel='Y',
                            title='', colorbar_label='Relative density',
                            figsize=(6, 5), label_fontsize=12)
```

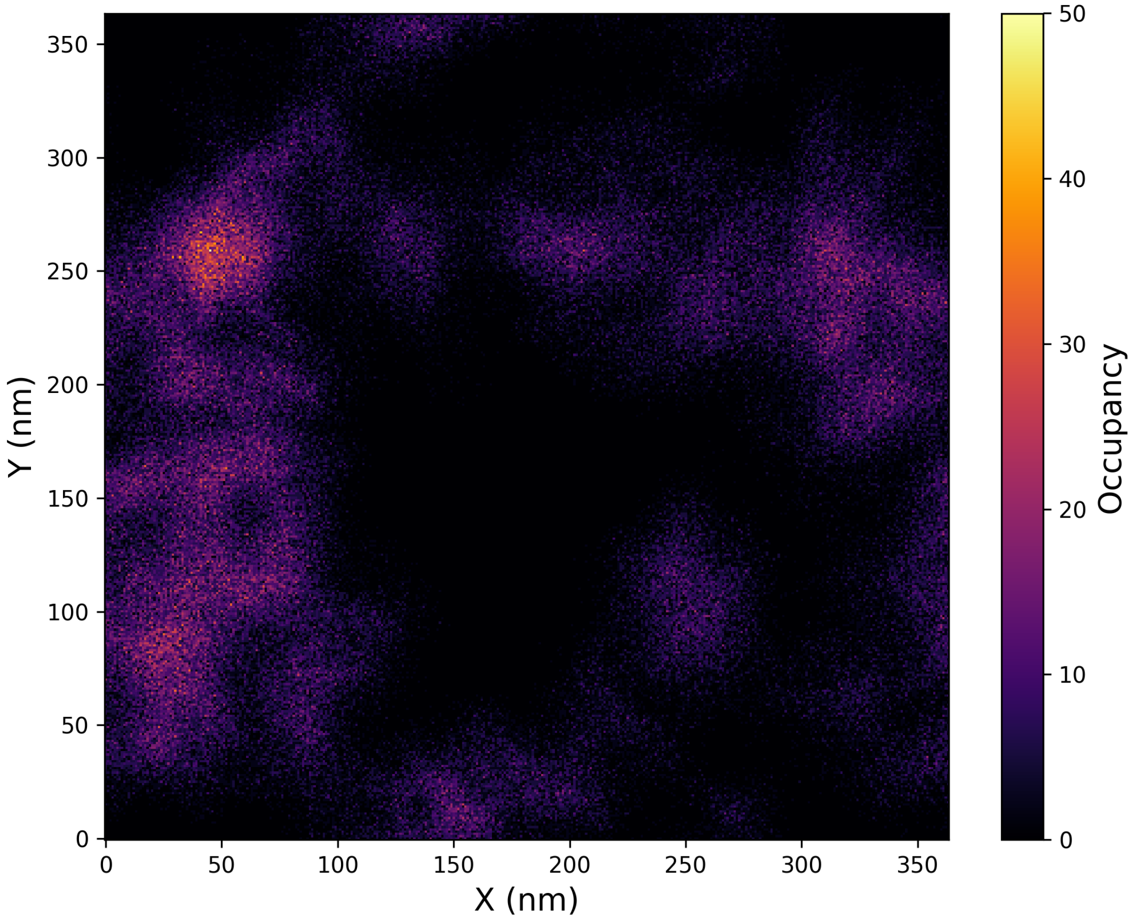

Interpretation guidance: This plot illustrates the relative density distribution of ligand atoms throughout the simulation. Regions with higher density values correspond to areas where the ligand spent more time, indicating preferred binding locations or stable interaction zones. Conversely, areas with lower density indicate less frequent ligand presence or greater positional variability.

17.2 Complete code

```
import numpy as np
import matplotlib.pyplot as plt
import os
```

```
def read_xpm(file_path):
```

```
"""
Reads a .xpm file and converts it into a 2D matrix of density values.

Parameters:
-----------
file_path : str
    Path to the .xpm file.

Returns:
--------
matrix : np.ndarray
    2D array of density values.
```

(continues on next page)

(continued from previous page)

```
"""
with open(file_path, 'r') as f:
    lines = f.readlines()

# Keep only lines containing matrix content (i.e., starting with quotes)
lines = [l.strip() for l in lines if l.strip().startswith('"')]

# Parse the header line with matrix dimensions and color mapping details
header_line = lines[0].strip().strip('",')
width, height, ncolors, chars_per_pixel = map(int, header_line.split())

# Build the color symbol to value mapping
color_map = {}
for i in range(1, ncolors + 1):
    line = lines[i].strip().strip('",')
    symbol = line[:chars_per_pixel]
    color_map[symbol] = i - 1  # Assign unique integer values for plotting

# Convert the symbol matrix into a numeric matrix
matrix = []
for line in lines[ncolors + 1:]:
    line = line.strip().strip('",')
    row = [color_map[line[i:i+chars_per_pixel]] for i in range(0, len(line), chars_
↪per_pixel)]
    matrix.append(row)

return np.array(matrix)
```

```
def plot_density(matrix, cmap='inferno', xlabel='X', ylabel='Y', title='',
                 colorbar_label='Relative density', figsize=(6, 5),
                 label_fontsize=12, save_path=None):
```

```
"""
Plots the ligand density matrix as a heatmap.

Parameters:
-----------
matrix : np.ndarray
    The ligand density matrix.
cmap : str
    Colormap used for the heatmap (e.g., 'inferno', 'jet').
xlabel, ylabel : str
    Axis labels for the plot.
title : str
    Title of the plot.
colorbar_label : str
    Label for the colorbar.
figsize : tuple
    Size of the figure (width, height in inches).
label_fontsize : int
    Font size used for all text labels.
save_path : str or None
    Path (without extension) where the plot will be saved.
"""
plt.figure(figsize=figsize)
img = plt.imshow(matrix, cmap=cmap, origin='lower', aspect='auto')
```

(continues on next page)

(continued from previous page)

```
cbar = plt.colorbar(img)
cbar.set_label(colorbar_label, fontsize=label_fontsize)
plt.xlabel(xlabel, fontsize=label_fontsize)
plt.ylabel(ylabel, fontsize=label_fontsize)
plt.title(title, fontsize=label_fontsize + 2)
plt.tight_layout()

# Save plots in both PNG and TIFF formats
if save_path:
    base, _ = os.path.splitext(save_path)
    plt.savefig(f"{base}.png", dpi=300)
    plt.savefig(f"{base}.tiff", dpi=300)
    print(f"Figure saved as: {base}.png and {base}.tiff")

plt.show()
```

```
def ligand_density_analysis(xpm_file_path, output_path=None, plot=True,
                            cmap='inferno', xlabel='X', ylabel='Y',
                            title='', colorbar_label='Relative density',
                            figsize=(6, 5), label_fontsize=12):
```

```
"""
Main function to perform ligand density analysis from an .xpm file.

Parameters:
-----------
xpm_file_path : str
    Path to the input .xpm file.
output_path : str or None
    Base output path (without extension) to save figures.
plot : bool
    If True, displays the density plot.
cmap : str
    Colormap to use for the heatmap visualization.
xlabel, ylabel, title, colorbar_label : str
    Custom text for axis labels, title, and colorbar.
figsize : tuple
    Figure size in inches (width, height).
label_fontsize : int
    Font size for axis labels and title.

Returns:
--------
matrix : np.ndarray
    2D array representing the ligand density.
"""
matrix = read_xpm(xpm_file_path)
if plot:
    plot_density(matrix, cmap=cmap, xlabel=xlabel, ylabel=ylabel,
                 title=title, colorbar_label=colorbar_label,
                 figsize=figsize, label_fontsize=label_fontsize,
                 save_path=output_path)
return matrix
```

18

Ligand Angle

18.1 Overview

DynamiSpectra provides a comprehensive and versatile analytical framework designed to quantify and monitor the spatial distribution and dynamics of ligand angular conformations throughout molecular dynamics simulations. Utilizing input data in standardized .xvg file format, this module enables researchers to characterize ligand conformational preferences, rotational flexibility, and dynamic behavior within the simulated environment.

This analysis delivers detailed insights into the distribution and evolution of ligand angles, facilitating identification of predominant conformations, conformational transitions, or rare states. The graphical outputs generated by *DynamiSpectra* support clear visualization of ligand angular patterns over time, enabling both qualitative and quantitative interpretation of ligand structural dynamics.

Command line in GROMACS to generate .xvg files for the analysis:

```
gmx angle -f Simulation.xtc -n Simulation.ndx -type dihedral -od diedro_ligand.xvg
```

Note: The .ndx file must include a group containing the specific atoms that define the dihedral angle of interest for the ligand. Proper selection of these atoms is essential for accurate calculation and analysis of ligand dihedral angles.

```
def angle_ligand_analysis(output_folder, *simulation_groups, time_config=None,␣
↪density_config=None, kde2d_config=None)
```

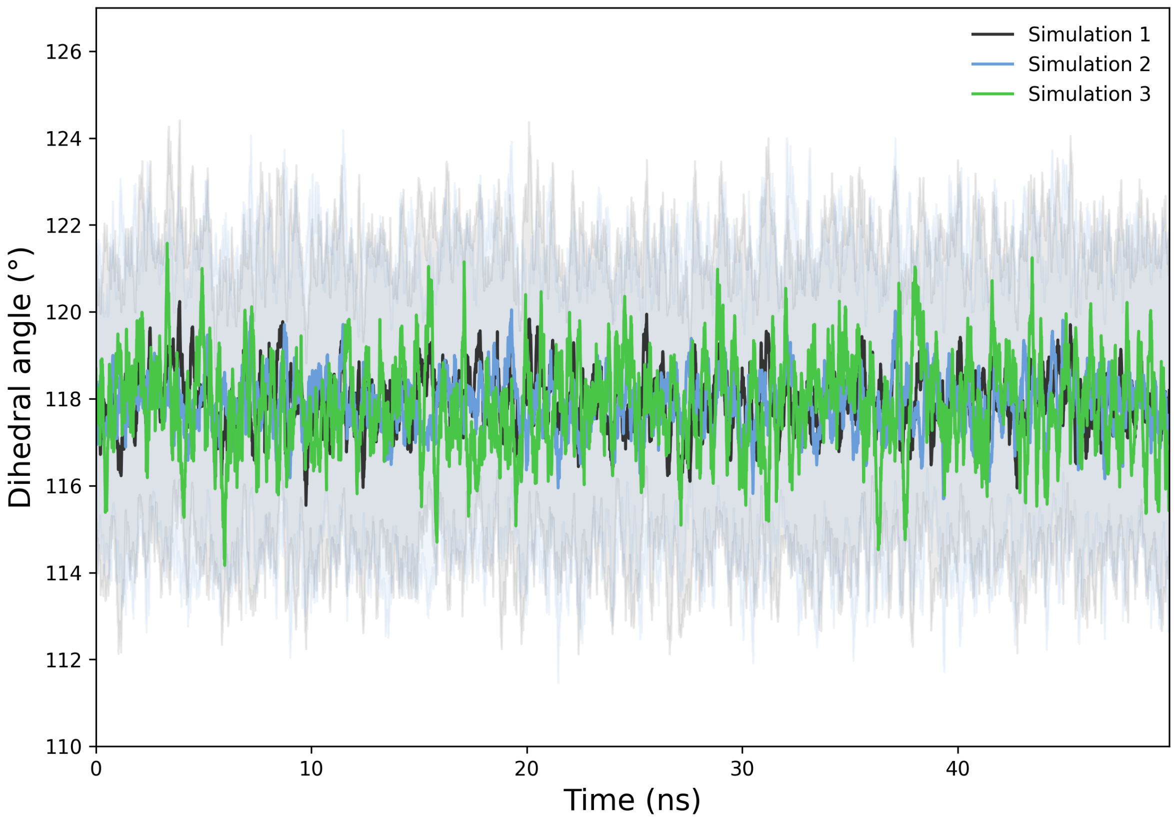

Interpretation guidance: This plot shows the temporal evolution of the ligand's dihedral angle across simulations. The consistent range and overlapping curves suggest similar torsional behavior. Fluctuations indicate dynamic flexibility within the sampled timescale.

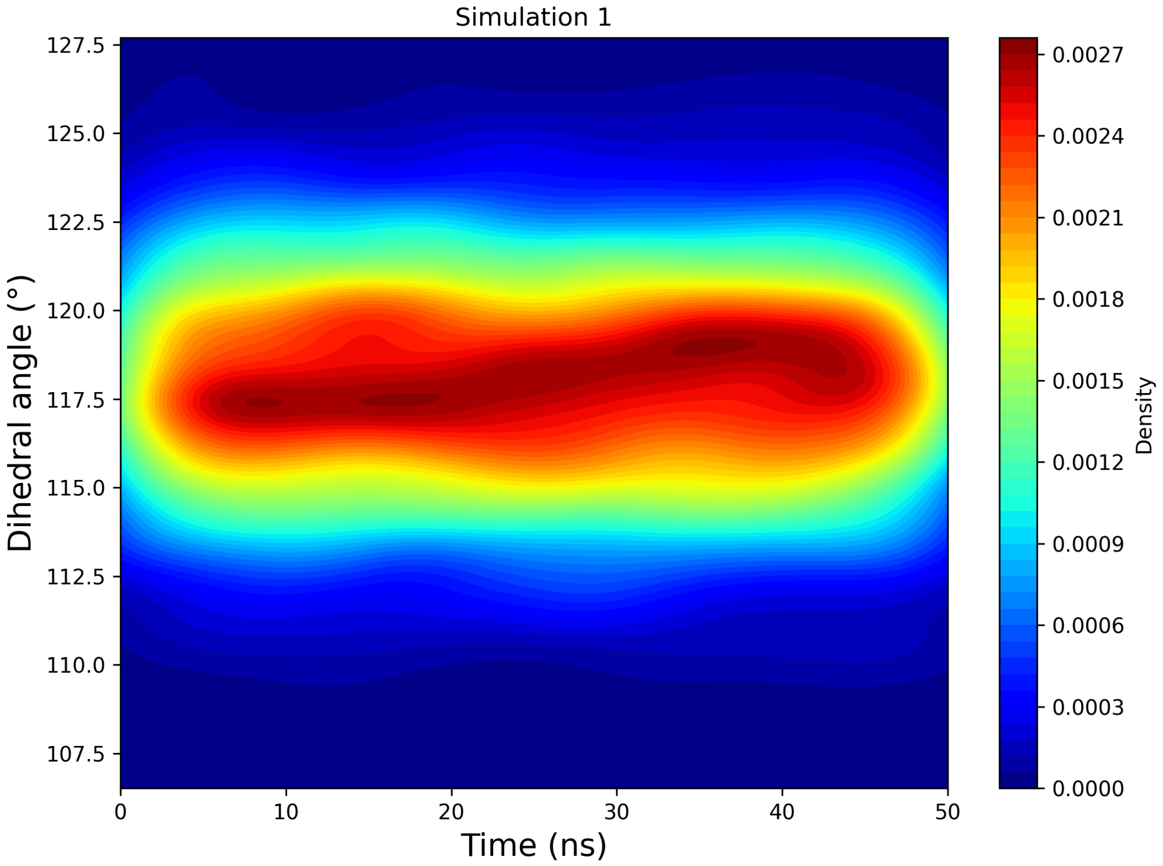

Interpretation guidance: This 2D KDE plot represents the temporal evolution of the ligand's dihedral angle, computed as the circular mean across all replicas within each simulation group. If a simulation includes six replicas, their angular profiles are combined through circular averaging to generate a single, smoothed density map. The result reveals dominant conformational states and transitions over time, highlighting regions of stability and fluctuation.

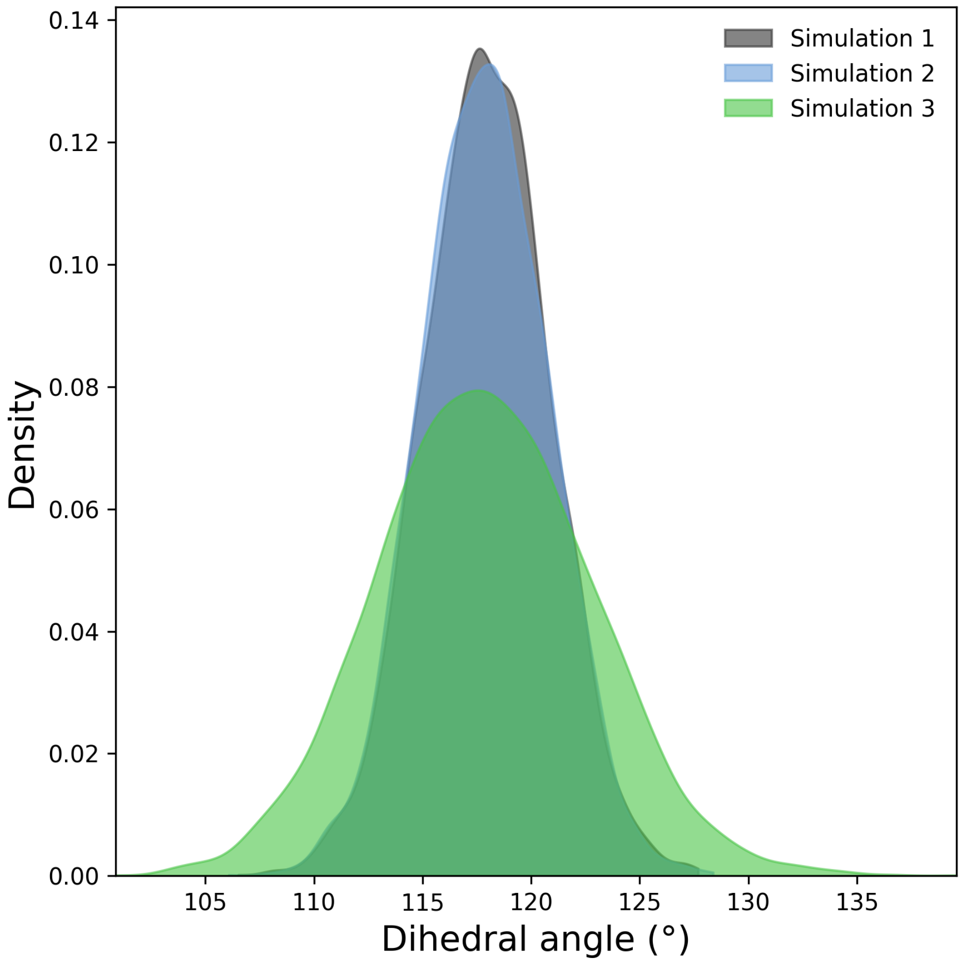

Interpretation guidance: The density plot compares the overall distribution of dihedral angles across simulations. A narrow peak suggests rigidity, while broader or multiple peaks indicate flexible or metastable conformations. Differences between curves reflect variability across replicates.

18.2 Complete code

```
import numpy as np
import matplotlib.pyplot as plt
from scipy.stats import gaussian_kde
import os
```

```
def read_angle(file):
```

```
try:
    print(f"Reading file: {file}")
    times, angles = [], []
    with open(file, 'r') as f:
        for line in f:
            if line.startswith(('#', '@', ';')) or line.strip() == '':
                continue
            try:
                time_ps, angle = map(float, line.split()[:2])
                times.append(time_ps * 0.001)  # convert ps to ns
                angles.append(angle)
```

(continues on next page)

(continued from previous page)

```
            except ValueError:
                print(f"Skipping invalid line: {line.strip()}")
    if not times or not angles:
        raise ValueError(f"No valid data in file: {file}")
    return np.array(times), np.array(angles)
except Exception as e:
    print(f"Error reading file {file}: {e}")
    return None, None
```

```
def check_simulation_times(*time_arrays):
```

```
for i in range(1, len(time_arrays)):
    if not np.allclose(time_arrays[0], time_arrays[i]):
        raise ValueError(f"Simulation times do not match between file 1 and file
↪{i+1}")
```

```
def downsample(data, step=10):
```

```
return data[::step]
```

```
def moving_average(data, window_size=20):
```

```
return np.convolve(data, np.ones(window_size)/window_size, mode='valid')
```

```
def plot_time_series(results, output_folder, config):
```

```
plt.figure(figsize=config.get('figsize', (8, 5)))

colors = config.get('colors', ['#1f77b4', '#ff7f0e', '#2ca02c'])
alpha = config.get('alpha', 0.2)
labels = config.get('labels', [f"Simulation {i+1}" for i in range(len(results))])
fontsize = config.get('label_fontsize', 12)
step = config.get('downsample_step', 10)
smooth_window = config.get('smooth_window', 20)

for i, (time, mean, std) in enumerate(results):
    time_ds = downsample(time, step)
    mean_ds = downsample(mean, step)
    std_ds = downsample(std, step)

    mean_smooth = moving_average(mean_ds, smooth_window)
    std_smooth = moving_average(std_ds, smooth_window)
    time_smooth = time_ds[:len(mean_smooth)]

    plt.plot(time_smooth, mean_smooth, label=labels[i], color=colors[i %
↪len(colors)])
    plt.fill_between(time_smooth, mean_smooth - std_smooth, mean_smooth + std_
↪smooth,
                     color=colors[i % len(colors)], alpha=alpha)

plt.xlim(time_smooth[0], time_smooth[-1])
ymin = min([np.min(mean - std) for _, mean, std in results])
ymax = max([np.max(mean + std) for _, mean, std in results])
plt.ylim(ymin, ymax)
```

(continues on next page)

(continued from previous page)

```
plt.xlabel(config.get('xlabel', 'Time (ns)'), fontsize=fontsize)
plt.ylabel(config.get('ylabel', 'Dihedral angle (°)'), fontsize=fontsize)
plt.legend(frameon=False, fontsize=10)
plt.tick_params(axis='both', which='major', labelsize=10)
plt.tight_layout()

os.makedirs(output_folder, exist_ok=True)
plt.savefig(os.path.join(output_folder, 'angle_over_time.png'), dpi=300)
plt.savefig(os.path.join(output_folder, 'angle_over_time.tiff'), dpi=300)
plt.show()
```

```
def plot_density(distributions, output_folder, config):
```

```
plt.figure(figsize=config.get('figsize', (6, 5)))

colors = config.get('colors', ['#1f77b4', '#ff7f0e', '#2ca02c'])
alpha = config.get('alpha', 0.4)
labels = config.get('labels', [f"Simulation {i+1}" for i in␣
↪range(len(distributions))])
fontsize = config.get('label_fontsize', 12)

for i, dist in enumerate(distributions):
    kde = gaussian_kde(dist)
    x = np.linspace(min(dist), max(dist), 1000)
    plt.fill_between(x, kde(x), color=colors[i % len(colors)], alpha=alpha,␣
↪label=labels[i])
    plt.xlim(min(dist), max(dist))

plt.ylim(0, None)
plt.xlabel(config.get('xlabel', 'Dihedral angle (°)'), fontsize=fontsize)
plt.ylabel(config.get('ylabel', 'Density'), fontsize=fontsize)
plt.legend(frameon=False, fontsize=10)
plt.tick_params(axis='both', which='major', labelsize=10)
plt.tight_layout()

os.makedirs(output_folder, exist_ok=True)
plt.savefig(os.path.join(output_folder, 'angle_density.png'), dpi=300)
plt.savefig(os.path.join(output_folder, 'angle_density.tiff'), dpi=300)
plt.show()
```

```
def plot_kde_2d_time_angle(times, angles, output_folder, config, label, color):
```

```
times_ds = times
angles_ds = angles

xmin, xmax = times_ds.min(), times_ds.max()
ymin, ymax = angles_ds.min(), angles_ds.max()

xx, yy = np.mgrid[xmin:xmax:300j, ymin:ymax:300j]
positions = np.vstack([xx.ravel(), yy.ravel()])
values = np.vstack([times_ds, angles_ds])
kernel = gaussian_kde(values)
f = np.reshape(kernel(positions).T, xx.shape)
```

(continues on next page)

(continued from previous page)

```
plt.figure(figsize=config.get('figsize', (8, 6)))
cmap = config.get('cmap', 'viridis')
cf = plt.contourf(xx, yy, f, levels=50, cmap=cmap)
plt.colorbar(cf, label='Density')

plt.xlabel(config.get('xlabel', 'Time (ns)'), fontsize=config.get('label_fontsize',
↪ 12))
plt.ylabel(config.get('ylabel', 'Dihedral angle (°)'), fontsize=config.get('label_
↪fontsize', 12))
plt.title(label)
plt.tight_layout()

os.makedirs(output_folder, exist_ok=True)
filename = f"{label.replace(' ', '_').lower()}_kde_2d.png"
plt.savefig(os.path.join(output_folder, filename), dpi=300)
plt.show()
```

```
def angle_ligand_analysis(output_folder, *simulation_groups, time_config=None,␣
↪density_config=None, kde2d_config=None):
```

```
if time_config is None:
    time_config = {}
if density_config is None:
    density_config = {}
if kde2d_config is None:
    kde2d_config = {}

def process_group(file_list):
    times_all, angles_all = [], []
    for file in file_list:
        time, angle = read_angle(file)
        if time is None or angle is None:
            raise ValueError(f"Failed to read valid data from {file}")
        times_all.append(time)
        angles_all.append(angle)
    check_simulation_times(*times_all)

    angles_rad = np.radians(angles_all)
    mean_angles_rad = np.arctan2(np.mean(np.sin(angles_rad), axis=0),
                                 np.mean(np.cos(angles_rad), axis=0))
    mean_angles = np.degrees(mean_angles_rad)
    std_angles = np.std(angles_all, axis=0)

    return times_all[0], mean_angles, std_angles, mean_angles, angles_all

time_results = []
all_distributions = []
labels = kde2d_config.get('labels', [f"Simulation {i+1}" for i in␣
↪range(len(simulation_groups))])
colors = kde2d_config.get('colors', ['#1f77b4', '#ff7f0e', '#2ca02c'])

for idx, group in enumerate(simulation_groups):
    time, mean, std, mean_for_density, replicates = process_group(group)
    time_results.append((time, mean, std))
    all_distributions.append(mean_for_density)
```

(continues on next page)

(continued from previous page)

```
    angles_matrix = np.array(replicates)
    angles_rad = np.radians(angles_matrix)
    mean_angles_rad = np.arctan2(np.mean(np.sin(angles_rad), axis=0),
                                 np.mean(np.cos(angles_rad), axis=0))
    mean_angles_deg = np.degrees(mean_angles_rad)

    plot_kde_2d_time_angle(time, mean_angles_deg, output_folder, kde2d_config,␣
↪labels[idx], colors[idx % len(colors)])

plot_time_series(time_results, output_folder, time_config)
plot_density(all_distributions, output_folder, density_config)
```

19 Principal Component Analysis

19.1 Overview

DynamiSpectra offers a robust and versatile analytical framework for performing principal component analysis (PCA) on molecular dynamics simulation data. Utilizing input in standardized .xvg file format, this module enables researchers to identify and characterize the dominant collective motions and conformational fluctuations of biomolecular systems.

Command line in GROMACS to generate .xvg files for the analysis:

```
gmx covar -f Simulation.xtc -s Simulation.tpr -o eingenvalues.xvg -v eingenvectors.
↪trr -av average.pdb
```

```
gmx anaeig -v eingenvectors.trr -f Simulation.xtc -s Simulation.tpr -first 1 -last␣
↪2 -2d 2dproj.xvg
```

Note: PCA analysis requires the eigenvalues.xvg and 2dproj.xvg files.

```
def pca_analysis(pca_file_path, eigenval_path, output_folder,
             title="PCA", figsize=(7, 6), cmap="viridis",
             point_size=30, alpha=0.8):
```

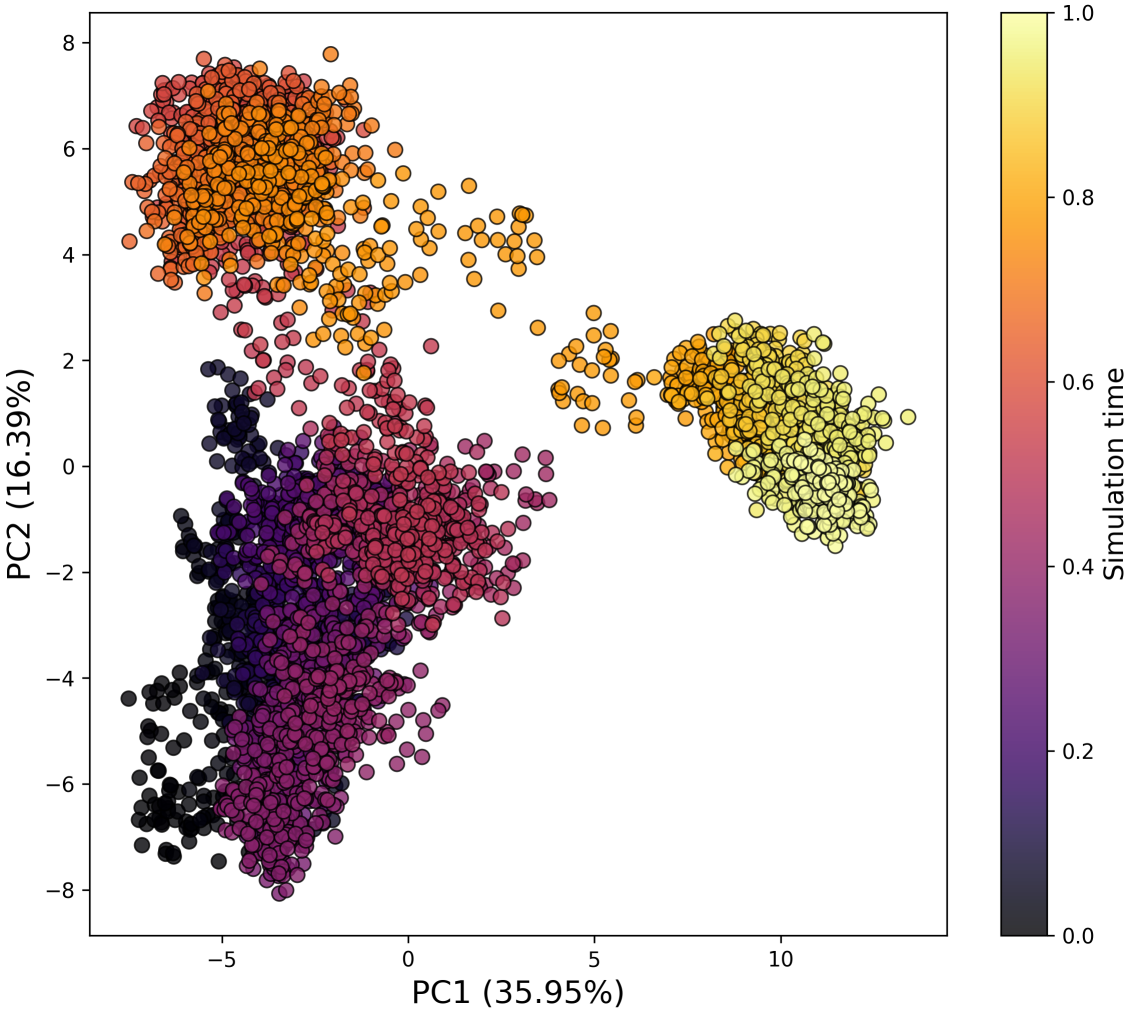

How to interpret: This plot illustrates the temporal or spatial distribution of motion projected along selected principal components (PCs). The first few PCs generally capture the most significant large-scale motions within the system. Dense clustering of data points may indicate stable conformational states, whereas more dispersed distributions suggest increased structural variability or transitions between different dynamic regimes.

19.2 Complete code

```
import numpy as np
import matplotlib.pyplot as plt
import os
```

```
def read_xvg(file_path):
```

```
"""
Reads PCA projection data from a .xvg file.

Parameters:
-----------
file_path : str
    Path to the .xvg file.

Returns:
```

(continues on next page)

(continued from previous page)

```
--------
data : numpy.ndarray
    Array with PC1 and PC2 values.
"""
data = []
with open(file_path, "r") as file:
    for line in file:
        # Skip comment and metadata lines
        if not line.startswith(("#", "@")):
            values = line.split()
            data.append([float(values[0]), float(values[1])])
return np.array(data)
```

```
def read_eigenvalues(file_path):
```

```
"""
Reads eigenvalues from a .xvg file.

Parameters:
-----------
file_path : str
    Path to the eigenvalues .xvg file.

Returns:
--------
eigenvalues : numpy.ndarray
    Array of eigenvalues.
"""
eigenvalues = []
with open(file_path, "r") as file:
    for line in file:
        # Skip comment and metadata lines
        if not line.startswith(("#", "@")):
            eigenvalues.append(float(line.split()[1]))
return np.array(eigenvalues)
```

```
def plot_pca(pca_data, eigenvalues, output_folder, title="PCA",
         figsize=(7, 6), cmap="viridis", point_size=30, alpha=0.8):
```

```
"""
Generates a PCA scatter plot with customization options.

Parameters:
-----------
pca_data : numpy.ndarray
    Array with PC1 and PC2 values.
eigenvalues : numpy.ndarray
    Array of eigenvalues.
output_folder : str
    Folder to save the output figure.
title : str
    Title of the plot.
figsize : tuple
    Size of the figure (width, height).
cmap : str
```

(continues on next page)

(continued from previous page)

```
    Color map for scatter points.
point_size : int or float
    Size of the scatter points.
alpha : float
    Transparency of the points (0 to 1).
"""
total_variance = np.sum(eigenvalues)
pc1_var = (eigenvalues[0] / total_variance) * 100
pc2_var = (eigenvalues[1] / total_variance) * 100

plt.figure(figsize=figsize)
scatter = plt.scatter(
    pca_data[:, 0], pca_data[:, 1],
    c=np.linspace(0, 1, len(pca_data)),  # Color by simulation time
    cmap=cmap,
    s=point_size,
    alpha=alpha,
    edgecolors='k',
    linewidths=0.8
)

plt.xlabel(f"PC1 ({pc1_var:.2f}%)", fontsize=12)
plt.ylabel(f"PC2 ({pc2_var:.2f}%)", fontsize=12)
plt.title(title, fontsize=14)
plt.colorbar(scatter, label="Simulation time")

plt.grid(False)
plt.tight_layout()

# Create the output folder if it does not exist
os.makedirs(output_folder, exist_ok=True)

# Save the figure in high resolution
plt.savefig(os.path.join(output_folder, 'pca_plot.png'), dpi=300)
plt.show()
```

```
def pca_analysis(pca_file_path, eigenval_path, output_folder,
                 title="PCA", figsize=(7, 6), cmap="viridis",
                 point_size=30, alpha=0.8):
```

```
"""
Runs PCA analysis and generates the scatter plot.

Parameters:
-----------
pca_file_path : str
    Path to the PCA projection file (usually 'pca_proj.xvg').
eigenval_path : str
    Path to the eigenvalues file (usually 'eigenval.xvg').
output_folder : str
    Folder to save the output plot.
title : str
    Plot title.
figsize : tuple
    Size of the figure.
cmap : str
```

(continues on next page)

(continued from previous page)

```
    Colormap for the scatter plot.
point_size : int or float
    Size of scatter points.
alpha : float
    Transparency of scatter points.
"""
pca_data = read_xvg(pca_file_path)
eigenvalues = read_eigenvalues(eigenval_path)
plot_pca(pca_data, eigenvalues, output_folder, title, figsize, cmap, point_size,␣
↪alpha)
```

20 Secondary Structure Probability

20.1 Overview

DynamiSpectra provides a comprehensive secondary structure analysis tool for molecular dynamics simulations based on .dat files. This module calculates the frame-wise probability of each structural state—such as α-helices, β-sheets, loops, turns, bends, and 3-helices—across simulation replicas. The results are visualized using boxplots, allowing for comparative analysis of secondary structure stability and population among different simulation groups.

```
def ss_analysis(output_folder, *simulation_files_groups, plot_config=None)
```

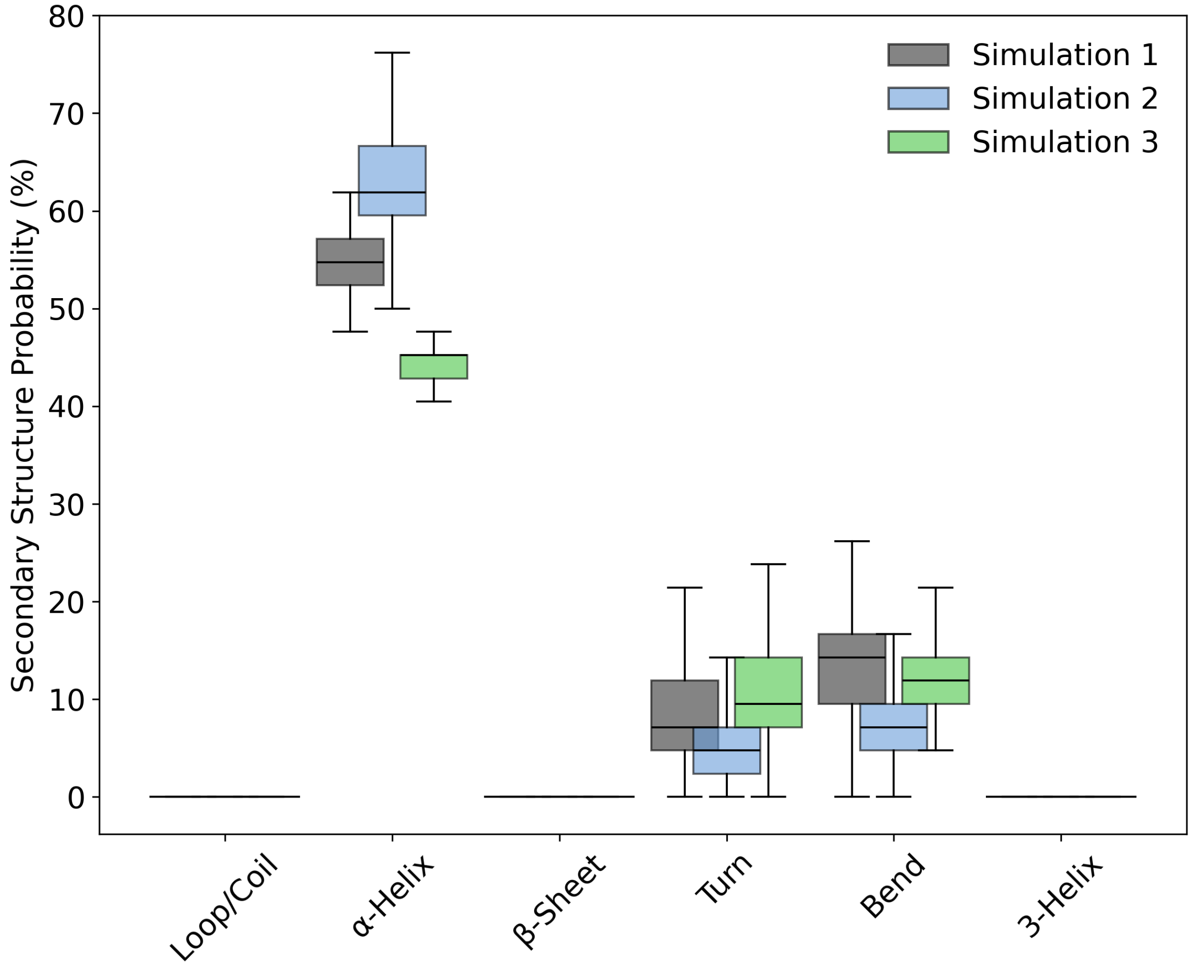

Interpretation guidance: This graph shows the probability distribution of each secondary structure type across the simulation. Variations in these probabilities can reflect structural stability or conformational changes over time. Consistent probabilities suggest stable elements, while shifts may indicate unfolding or rearrangements.

20.2 Complete code

```
import numpy as np
import matplotlib.pyplot as plt
from matplotlib.patches import Patch
import os
```

```
# Mapping DSSP secondary structure codes to numeric labels
state_mapping = {
'H': 1,   # Alpha helix
'E': 2,   # Beta sheet
'C': 0,   # Coil / Loop
'T': 3,   # Turn
'S': 4,   # Bend
'G': 5,   # 3-Helix
'~': -1,  # Unknown / missing data
'B': -1,  # Isolated beta-bridge (treated as unknown)
}

# Human-readable state names for plotting
state_names = {
0: 'Loop/Coil',
1: 'α-Helix',
2: 'β-Sheet',
3: 'Turn',
4: 'Bend',
5: '3-Helix',
}
```

```
def read_ss(file):
```

```
"""
Reads secondary structure assignments from a .dat file.

Parameters:
    file (str): Path to the secondary structure .dat file.

Returns:
    np.ndarray: 2D array with shape (frames x residues), numeric states.
"""
try:
    print(f"Reading file: {file}")
    ss_data = []
    with open(file, 'r') as f:
        for line in f:
            # Skip comment/header lines or empty lines
            if line.startswith(('#', '@', ';')) or line.strip() == '':
                continue
            # Map each character in line to numeric state, default -1 for unknown
            ss_line = [state_mapping.get(char, -1) for char in line.strip()]
            ss_data.append(ss_line)
    if len(ss_data) == 0:
```

(continues on next page)

(continued from previous page)

```
        raise ValueError(f"No valid data found in {file}")
    return np.array(ss_data)
except Exception as e:
    print(f"Error reading {file}: {e}")
    return None
```

```
def calculate_probabilities(ss_data):
```

```
"""
Calculate the per-frame probability of each secondary structure state.

Parameters:
    ss_data (np.ndarray): 2D array of secondary structure states (frames x␣
↪residues).

Returns:
    dict: Keys are state names, values are 1D arrays of per-frame probabilities.
"""
probabilities = {name: [] for name in state_names.values()}
for code, name in state_names.items():
    # Calculate fraction of residues in each state per frame
    probabilities[name] = np.sum(ss_data == code, axis=1) / ss_data.shape[1]
return probabilities
```

```
def plot_ss_boxplot(probabilities_list, labels, colors, output_folder,
                    alpha=0.7, axis_label_size=12, y_axis_label='Probability (%)',␣
↪figsize=(7, 6)):
```

```
"""
Generate a boxplot comparing secondary structure probabilities across simulations.

Parameters:
    probabilities_list (list of dicts): List of probability dicts from each␣
↪simulation group.
    labels (list of str): Labels for each simulation group.
    colors (list of str): Colors for each simulation group.
    output_folder (str): Directory to save plots.
    alpha (float): Transparency for boxplot fill colors.
    axis_label_size (int): Font size for axis labels.
    y_axis_label (str): Label for Y axis.
    figsize (tuple): Figure size (width, height).
"""
x_labels = list(state_names.values())
x = np.arange(len(x_labels))

plt.figure(figsize=figsize)

def plot_boxplot(data, positions, color):
    """
    Helper to plot one set of boxplots with given positions and color.
    """
    box = plt.boxplot(data, positions=positions, widths=0.4, patch_artist=True,
                      labels=[''] * len(positions), showfliers=False)
    for patch in box['boxes']:
        patch.set_facecolor(color)
```

(continues on next page)

(continued from previous page)

```
        patch.set_alpha(alpha)
    for median in box['medians']:
        median.set_color('black')
    return box

# Plot boxplots for each simulation group side-by-side
for i, (probs, label, color) in enumerate(zip(probabilities_list, labels, colors)):
    data = [probs[name] * 100 for name in x_labels]  # Convert fraction to␣
↪percentage
    plot_boxplot(data, x - 0.25 + i * 0.25, color)

plt.xlabel('', fontsize=axis_label_size)
plt.ylabel(y_axis_label, fontsize=axis_label_size)
plt.title('', fontsize=14, fontweight='bold')
plt.xticks(x, x_labels, rotation=45, fontsize=axis_label_size)
plt.yticks(fontsize=axis_label_size)
plt.grid(False)

# Create legend patches for simulation groups
legend_elements = [
    Patch(facecolor=color, edgecolor='black', linewidth=1.2, alpha=alpha,␣
↪label=label)
    for label, color in zip(labels, colors)
]
plt.legend(handles=legend_elements, frameon=False, fontsize=axis_label_size, loc=
↪'upper right')

plt.tight_layout()
os.makedirs(output_folder, exist_ok=True)
plt.savefig(os.path.join(output_folder, 'secondary_structure_boxplot.png'),␣
↪dpi=300, bbox_inches='tight')
plt.savefig(os.path.join(output_folder, 'secondary_structure_boxplot.tiff'),␣
↪dpi=300, bbox_inches='tight')
plt.show()
```

```
def ss_analysis(output_folder, *simulation_files_groups, plot_config=None):
```

```
"""
Main function to perform secondary structure analysis.

Parameters:
    output_folder (str): Directory to save generated plots.
    *simulation_files_groups: Variable number of groups of replicate file paths␣
↪(lists of str).
    plot_config (dict, optional): Configuration for plotting, supports:
        - labels (list of str): Names of simulation groups.
        - colors (list of str): Colors for groups.
        - alpha (float): Transparency for boxplots.
        - axis_label_size (int): Font size for axis labels.
        - y_axis_label (str): Label for Y axis.
        - figsize (tuple): Figure size (width, height).
"""
if plot_config is None:
    plot_config = {}

num_groups = len(simulation_files_groups)
```

(continues on next page)

(continued from previous page)

```
labels = plot_config.get('labels', [f'Simulation {i+1}' for i in range(num_
↪groups)])
colors = plot_config.get('colors', ['#333333', '#6A9EDA', '#54b36a'][:num_groups])
alpha = plot_config.get('alpha', 0.7)
axis_label_size = plot_config.get('axis_label_size', 12)
y_axis_label = plot_config.get('y_axis_label', 'Probability (%)')
figsize = plot_config.get('figsize', (7, 6))

def process_group(file_paths):
    """
    Read and aggregate secondary structure data from replicate files in one␣
↪simulation group.

    Parameters:
        file_paths (list of str): Paths to replicate .dat files.

    Returns:
        np.ndarray: Secondary structure data array (frames x residues) from the␣
↪first replicate.
                    (Assuming replicates have the same data format.)
    """
    ss_data = []
    for file in file_paths:
        data = read_ss(file)
        if data is not None:
            ss_data.append(data)
    # For simplicity, use the first replicate data (assuming replicates have the␣
↪same data)
    return ss_data[0] if ss_data else None

probabilities_list = []
for group in simulation_files_groups:
    if group:
        ss_data = process_group(group)
        if ss_data is not None:
            probabilities = calculate_probabilities(ss_data)
            probabilities_list.append(probabilities)

plot_ss_boxplot(probabilities_list, labels, colors, output_folder,
                alpha=alpha, axis_label_size=axis_label_size,
                y_axis_label=y_axis_label, figsize=figsize)
```

21

Secondary Structure Fraction

21.1 Overview

DynamiSpectra provides a comprehensive secondary structure analysis module tailored for molecular dynamics simulations. This module processes simulation data to quantify the fraction of each secondary structure type—such as α-helices, β-sheets, and loops—across all trajectory frames. The resulting time-resolved plots enable detailed visualization of secondary structure composition dynamics throughout the simulation.

Command line in GROMACS to generate .xvg files for the analysis:

```
gmx dssp -s Simulation.tpr -f Simulation.xtc -o ss.dat
```

```
def fractions_ss_analysis(file_path, output_folder, plot_config=None)
```

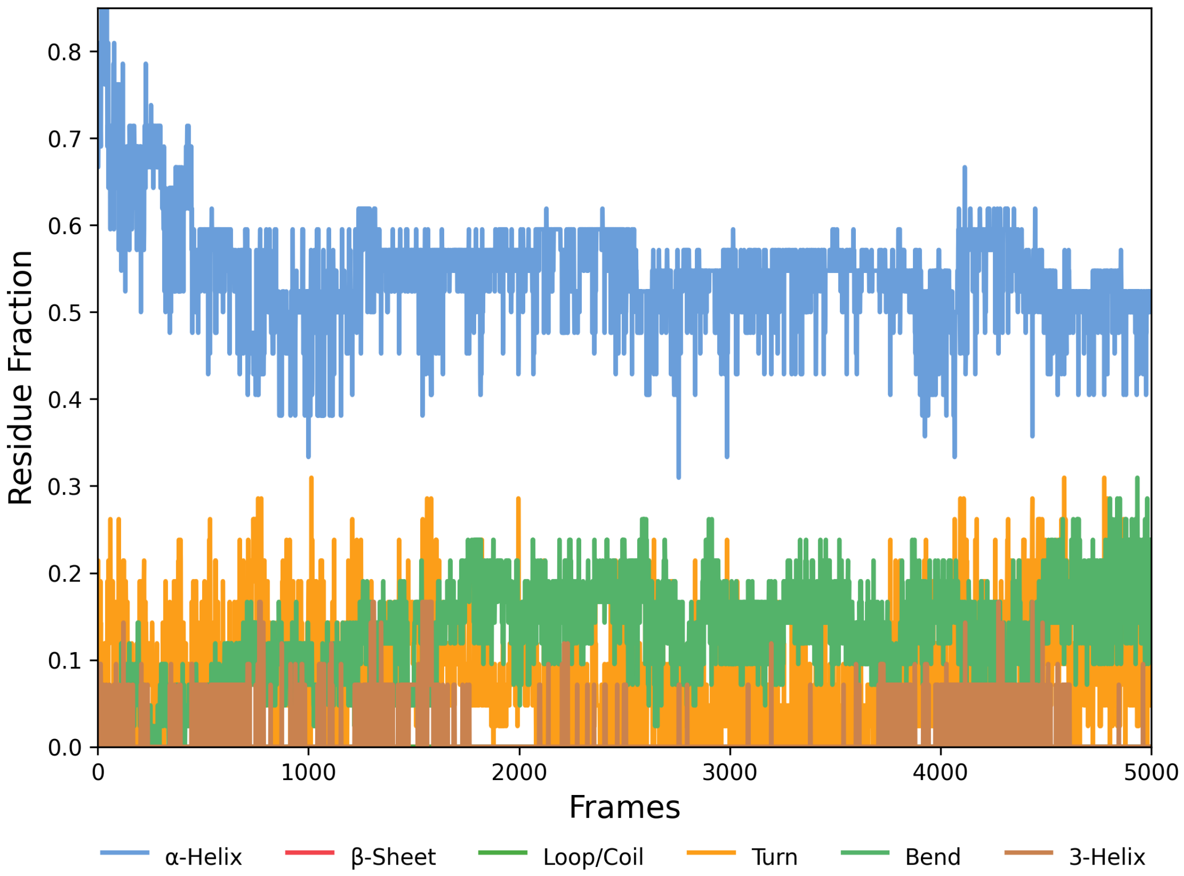

Interpretation guidance: The presented graph illustrates the temporal evolution of the fractions of residues adopting different secondary structure elements. Monitoring these fluctuations facilitates assessment of structural stability and conformational dynamics within the system. Consistent patterns may indicate stable folding, while variations can reveal conformational transitions or local instabilities. Comparative analysis across simulations aids in identifying reproducibility and the effects of differing initial conditions or parameters. Observed changes often correspond to the formation or disruption of critical structural motifs, thereby providing valuable insights into the molecular behavior under investigation.

Note: The .dat file contains raw data from the molecular dynamics simulation, classifying the secondary structure of each protein residue across all frames. The code reads this file and, for each frame, counts residues in each secondary structure type (e.g., α-helices, β-sheets, loops). It then calculates the fraction of each structure by dividing the count by the total number of residues. These fractions are plotted over time, showing how secondary structure proportions evolve during the simulation.

21.2 Complete code

```
import pandas as pd
import matplotlib.pyplot as plt
import os
```

```
def load_data(file_path):
```

```
"""
Loads the secondary structure .dat file into a DataFrame.

Parameters:
-----------
```

(continues on next page)

(continued from previous page)

```
file_path : str
    Path to the .dat file.

Returns:
--------
df : pd.DataFrame
    DataFrame with one row per frame containing secondary structure string.
"""
try:
    df = pd.read_csv(file_path, header=None)
    print("First few rows of the file:")
    print(df.head())
    return df
except Exception as e:
    print(f"Error loading the file: {e}")
    return None
```

```
def calculate_fractions(df):
```

```
"""
Calculates the fraction of each secondary structure over time.

Parameters:
-----------
df : pd.DataFrame
    DataFrame with one row per frame containing the structure string.

Returns:
--------
results_df : pd.DataFrame
    DataFrame with fraction values for each structure type over time.
"""
time = []
helix_fraction = []
sheet_fraction = []
coil_fraction = []
turn_fraction = []
bend_fraction = []
three_helix_fraction = []

# Iterate over each time step (row)
for index, row in df.iterrows():
    sequence = row[0]
    total_residues = len(sequence)

    # Calculate fraction of each structure
    helix_fraction.append(sequence.count("H") / total_residues)
    sheet_fraction.append(sequence.count("E") / total_residues)
    coil_fraction.append(sequence.count("C") / total_residues)
    turn_fraction.append(sequence.count("T") / total_residues)
    bend_fraction.append(sequence.count("S") / total_residues)
    three_helix_fraction.append(sequence.count("G") / total_residues)
    time.append(index)

# Create DataFrame with results
results_df = pd.DataFrame({
```

(continues on next page)

(continued from previous page)

```
    "Time": time,
    "Helix Fraction": helix_fraction,
    "Sheet Fraction": sheet_fraction,
    "Coil Fraction": coil_fraction,
    "Turn Fraction": turn_fraction,
    "Bend Fraction": bend_fraction,
    "3-Helix Fraction": three_helix_fraction
})

print("First few rows of the results:")
print(results_df.head())
return results_df
```

```
def plot_results(results_df, output_folder, plot_config):
```

```
"""
Plots the fractions of each secondary structure type over time.

Parameters:
-----------
results_df : pd.DataFrame
    DataFrame with computed fractions.
output_folder : str
    Path to save the output plots and Excel file.
plot_config : dict
    Dictionary with plot customization (title, labels, colors, etc).
"""
# Retrieve plot configuration
title = plot_config.get('title', 'Secondary Structure Fractions')
xlabel = plot_config.get('xlabel', 'Frames')
ylabel = plot_config.get('ylabel', 'Fraction of Residues')
figsize = plot_config.get('figsize', (7, 6))
fontsize = plot_config.get('fontsize', 12)
xlim = plot_config.get('xlim', None)
ylim = plot_config.get('ylim', (0, 0.85))

plt.figure(figsize=figsize)

# Plot each structure with distinct color and label
plt.plot(results_df["Time"], results_df["Helix Fraction"], label="α-Helix", color="
↪#6A9EDA", linewidth=2)
plt.plot(results_df["Time"], results_df["Sheet Fraction"], label="β-Sheet", color="
↪#f2444d", linewidth=2)
plt.plot(results_df["Time"], results_df["Coil Fraction"], label="Loop/Coil", color=
↪"#4bab44", linewidth=2)
plt.plot(results_df["Time"], results_df["Turn Fraction"], label="Turn", color="
↪#fc9e19", linewidth=2)
plt.plot(results_df["Time"], results_df["Bend Fraction"], label="Bend", color="
↪#54b36a", linewidth=2)
plt.plot(results_df["Time"], results_df["3-Helix Fraction"], label="3-Helix",␣
↪color="#c9824f", linewidth=2)

# Set axis labels and title
plt.xlabel(xlabel, fontsize=fontsize)
plt.ylabel(ylabel, fontsize=fontsize)
plt.title(title)
```

(continues on next page)

(continued from previous page)

```
plt.grid(False)

if xlim:
    plt.xlim(xlim)
if ylim:
    plt.ylim(ylim)

# Set legend at bottom center with multiple columns
plt.legend(loc="lower center", bbox_to_anchor=(0.5, -0.2), ncol=6, frameon=False,
           markerscale=2, handlelength=2, handleheight=2)

# Create output folder if it doesn't exist
os.makedirs(output_folder, exist_ok=True)

# Save plots
png_path = os.path.join(output_folder, 'secondary_structure_fractions.png')
tiff_path = os.path.join(output_folder, 'secondary_structure_fractions.tiff')
plt.savefig(png_path, dpi=300, bbox_inches='tight')
plt.savefig(tiff_path, dpi=300, bbox_inches='tight')
plt.show()

# Save data to Excel
excel_path = os.path.join(output_folder, 'secondary_structure_fractions.xlsx')
results_df.to_excel(excel_path, index=False)
print(f"Excel file saved: {excel_path}")
```

```
def fractions_ss_analysis(file_path, output_folder, plot_config=None):
```

```
"""
Main function to perform secondary structure fraction analysis.

Parameters:
-----------
file_path : str
    Path to the input .dat file with secondary structure data.
output_folder : str
    Directory where plots and result files will be saved.
plot_config : dict, optional
    Customization for the plot appearance:
        - title, xlabel, ylabel
        - figsize, fontsize
        - xlim, ylim
"""
if plot_config is None:
    plot_config = {}

df = load_data(file_path)
if df is not None:
    results_df = calculate_fractions(df)
    plot_results(results_df, output_folder, plot_config)
```

22 System Pressure

22.1 Overview

DynamiSpectra provides an analytical tool to evaluate pressure profiles across molecular dynamics simulations using .xvg files. The module calculates the mean pressure from multiple replicates and displays a time series plot with the standard deviation as a shaded region, helping to assess stability and fluctuations over time.

In addition to ensemble analysis, *DynamiSpectra* supports individual plots for each replicate and generates kernel density plots to visualize the distribution of pressure values. These outputs offer insight into system equilibrium, variability between replicates, and potential instabilities during the simulation.

Command line in GROMACS to generate .xvg files for the analysis:

```
gmx energy -f Simulation.edr -o pressure_simulation.xvg
```

```
def pressure_analysis(output_folder, *simulation_file_groups, pressure_config=None,
↪ density_config=None)
```

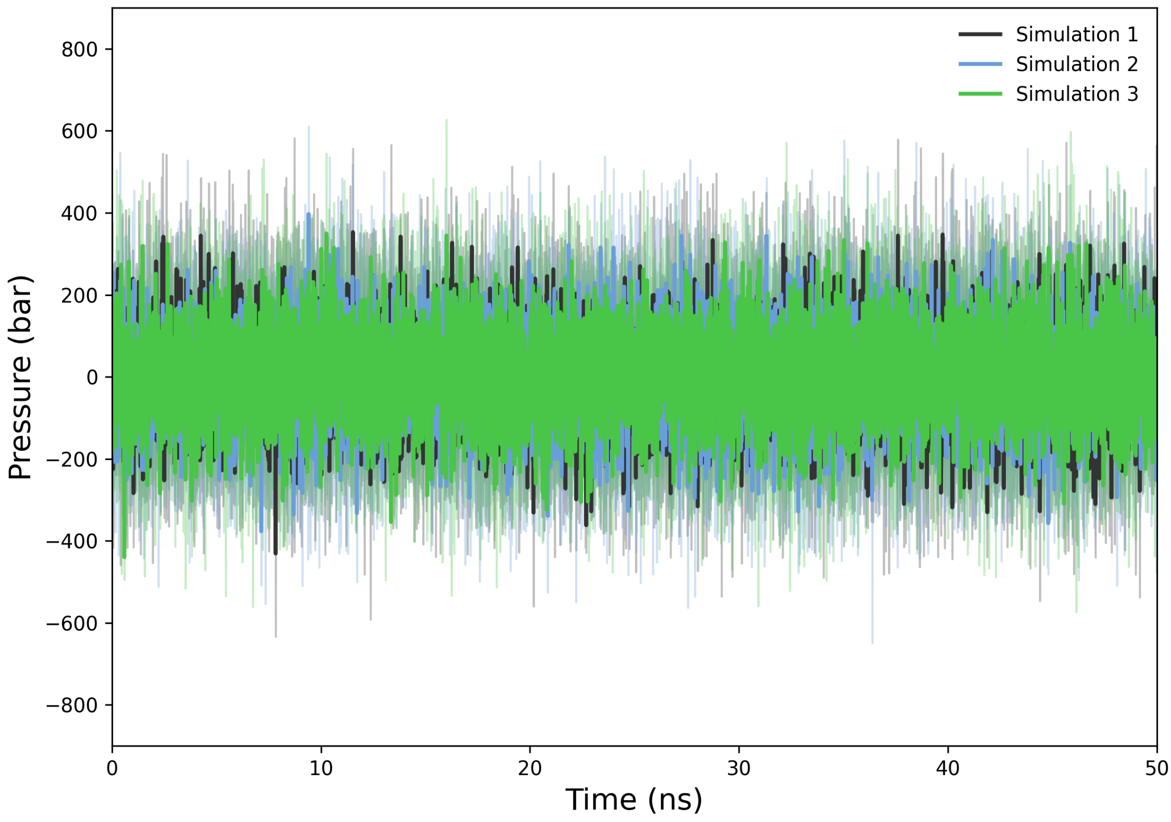

Interpretation guidance: The resulting plots illustrate the temporal evolution of system pressure. A stable pressure profile over time suggests that the system has reached equilibrium and is maintaining consistent thermodynamic conditions. In contrast, large fluctuations or drifts may indicate issues with system stability, improper equilibration, or external perturbations influencing the simulation dynamics.

```
def plot_pressure_density(results, output_folder, config=None)
```

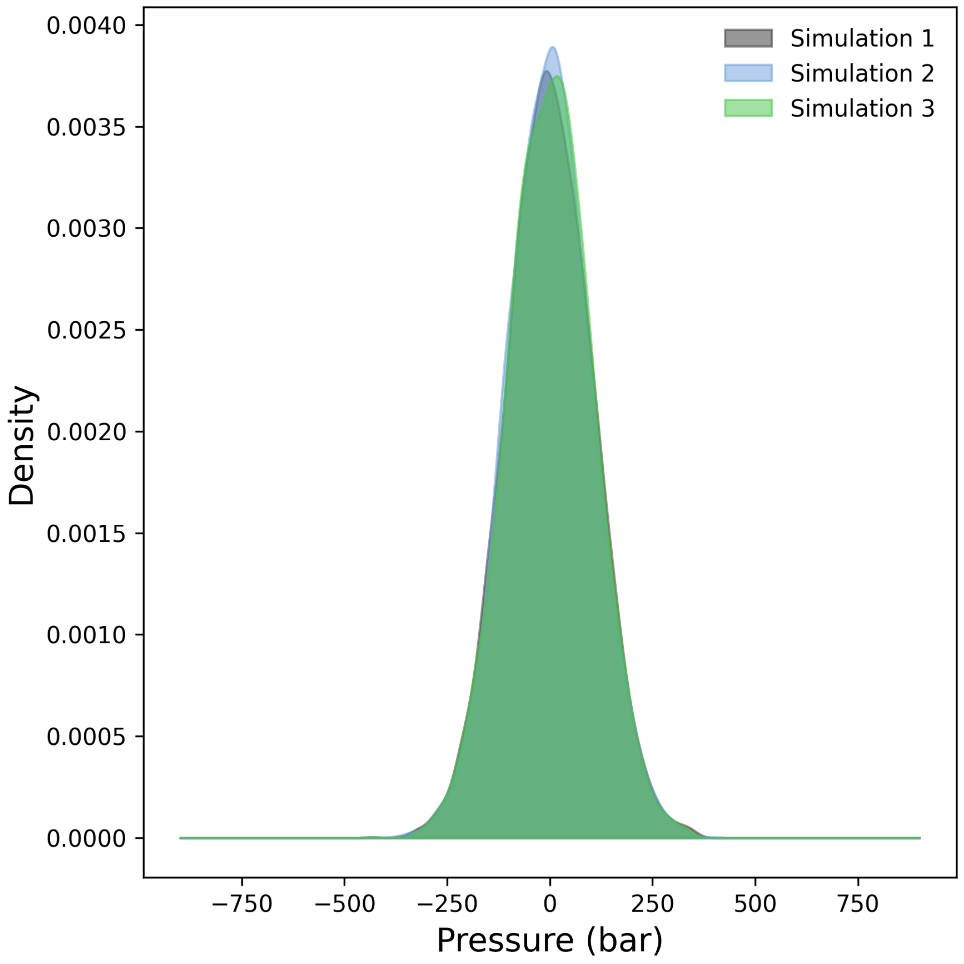

Interpretation guidance: This plot represents the pressure distribution derived from the temporal profile of system pressure. Peaks in the distribution indicate frequently sampled pressure values, reflecting the most common thermodynamic states of the system. Broader peaks suggest greater variability or fluctuations in pressure, while sharper, well-defined peaks indicate stable and consistent pressure throughout the simulation.

22.2 Complete code

```
import numpy as np
import matplotlib.pyplot as plt
from scipy.stats import gaussian_kde
import os
```

```
def read_pressure(file):
```

```
"""
Reads pressure data from a .xvg file.

Parameters:
-----------
file : str
    Path to the .xvg pressure data file.

Returns:
--------
```

(continues on next page)

(continued from previous page)

```
times : np.ndarray
    Time points converted from picoseconds to nanoseconds.
pressures : np.ndarray
    Corresponding pressure values in bar.
"""
try:
    times = []
    pressures = []
    with open(file, 'r') as f:
        for line in f:
            # Skip comment or empty lines
            if line.startswith(('#', '@', ';')) or line.strip() == '':
                continue
            try:
                values = line.split()
                if len(values) >= 2:
                    time_ps, pressure_val = map(float, values[:2])
                    times.append(time_ps / 1000.0)  # Convert ps to ns
                    pressures.append(pressure_val)
            except ValueError:
                # Ignore lines with parsing errors
                continue
    if len(times) == 0 or len(pressures) == 0:
        raise ValueError(f"File {file} does not contain valid data.")
    return np.array(times), np.array(pressures)
except Exception as e:
    print(f"Error reading file {file}: {e}")
    return None, None
```

```
def check_simulation_times(*time_arrays):
```

```
"""
Checks that all simulation time arrays match closely.

Parameters:
-----------
time_arrays : list of np.ndarray
    Variable number of time arrays to compare.

Raises:
-------
ValueError if any time arrays differ significantly.
"""
for i in range(1, len(time_arrays)):
    if not np.allclose(time_arrays[0], time_arrays[i]):
        raise ValueError(f"Simulation times do not match between file 1 and file
↪{i+1}")
```

```
def plot_pressure(results, output_folder, config=None):
```

```
"""
Plots time series of mean pressure with shaded standard deviation.

Parameters:
-----------
```

(continues on next page)

(continued from previous page)

```
results : list of tuples
    Each tuple contains (times, mean_pressure, std_pressure) arrays.
output_folder : str
    Directory to save plots.
config : dict, optional
    Plot configuration dictionary for labels, colors, alpha, figure size, axis␣
↪labels, etc.
"""
plt.figure(figsize=config.get('figsize', (9, 6)))

for idx, (time, mean, std) in enumerate(results):
    label = config['labels'][idx] if config and 'labels' in config else f
↪'Simulation {idx+1}'
    color = config['colors'][idx] if config and 'colors' in config else None
    alpha = config.get('alpha', 0.2)

    # Plot mean pressure line
    plt.plot(time, mean, label=label, color=color, linewidth=2)
    # Fill between mean ± std for shaded error region
    plt.fill_between(time, mean - std, mean + std, color=color, alpha=alpha)

plt.xlabel(config.get('xlabel', 'Time (ns)'), fontsize=config.get('label_fontsize',
↪ 12))
plt.ylabel(config.get('ylabel', 'Pressure (bar)'), fontsize=config.get('label_
↪fontsize', 12))
plt.legend(frameon=False, loc='upper right', fontsize=10)
plt.tick_params(axis='both', which='major', labelsize=10)

max_time = max([np.max(time) for time, _, _ in results])
plt.xlim(0, max_time)
if config and 'ylim' in config:
    plt.ylim(config['ylim'])

plt.tight_layout()
os.makedirs(output_folder, exist_ok=True)
plt.savefig(os.path.join(output_folder, 'pressure_plot.tiff'), dpi=300)
plt.savefig(os.path.join(output_folder, 'pressure_plot.png'), dpi=300)
plt.show()
```

```
def plot_pressure_density(results, output_folder, config=None):
```

```
"""
Plots kernel density estimates (KDE) of the pressure distributions.

Parameters:
-----------
results : list of tuples
    Each tuple contains (times, mean_pressure, std_pressure) arrays.
output_folder : str
    Directory to save plots.
config : dict, optional
    Plot configuration dictionary for labels, colors, alpha, figure size, axis␣
↪labels, etc.
"""
plt.figure(figsize=config.get('figsize', (6, 6)))
```

(continues on next page)

(continued from previous page)

```
for idx, (_, mean, _) in enumerate(results):
    kde = gaussian_kde(mean)  # Estimate density of mean pressures
    x_min = config.get('x_min', np.min(mean))
    x_max = config.get('x_max', np.max(mean))
    x_vals = np.linspace(x_min, x_max, 1000)

    label = config['labels'][idx] if config and 'labels' in config else f
↪'Simulation {idx+1}'
    color = config['colors'][idx] if config and 'colors' in config else None
    alpha = config.get('alpha', 0.5)

    # Fill area under KDE curve for density plot
    plt.fill_between(x_vals, kde(x_vals), color=color, alpha=alpha, label=label)

plt.xlabel(config.get('xlabel', 'Pressure (bar)'), fontsize=config.get('label_
↪fontsize', 12))
plt.ylabel(config.get('ylabel', 'Density'), fontsize=config.get('label_fontsize',␣
↪12))
plt.legend(frameon=False, loc='upper right', fontsize=10)
plt.tight_layout()

os.makedirs(output_folder, exist_ok=True)
plt.savefig(os.path.join(output_folder, 'pressure_density.tiff'), dpi=300)
plt.savefig(os.path.join(output_folder, 'pressure_density.png'), dpi=300)
plt.show()
```

```
def pressure_analysis(output_folder, *simulation_file_groups, pressure_config=None,
↪ density_config=None):
```

```
"""
Main function to analyze pressure data from multiple simulation groups.

Parameters:
-----------
output_folder : str
    Directory to save output plots.
simulation_file_groups : list of lists
    Variable number of simulation groups, each a list of .xvg pressure file paths.
pressure_config : dict, optional
    Configuration for time series pressure plot.
density_config : dict, optional
    Configuration for pressure density KDE plot.
"""
def process_group(file_paths):
    """
    Processes one simulation group of replicate files:
    - Reads time and pressure data
    - Checks time consistency
    - Calculates mean and standard deviation of pressure

    Returns:
    --------
    times : np.ndarray
        Time points (ns).
    mean_pressure : np.ndarray
        Mean pressure values.
```

(continues on next page)

(continued from previous page)

```
    std_pressure : np.ndarray
        Standard deviation of pressure.
    """
    times = []
    pressures = []
    for file in file_paths:
        time, pressure_val = read_pressure(file)
        times.append(time)
        pressures.append(pressure_val)
    check_simulation_times(*times)
    pressures_array = np.array(pressures)
    mean_pressure = np.mean(pressures_array, axis=0)
    std_pressure = np.std(pressures_array, axis=0)
    return times[0], mean_pressure, std_pressure

results = []
for group in simulation_file_groups:
    if group:
        time, mean, std = process_group(group)
        results.append((time, mean, std))

if len(results) >= 1:
    plot_pressure(results, output_folder, config=pressure_config)
    plot_pressure_density(results, output_folder, config=density_config)
else:
    raise ValueError("At least one simulation group is required.")
```

23

System Temperature

23.1 Overview

DynamiSpectra offers a dedicated module for analyzing system temperature across molecular dynamics simulations using .xvg files. This module computes the mean temperature from multiple replicates and presents a time-series plot with the standard deviation as a shaded region, enabling assessment of temperature stability and fluctuations throughout the simulation.

Alongside the time-resolved analysis, DynamiSpectra also generates kernel density plots to depict the overall temperature distribution. These outputs help identify equilibration status, detect anomalies across replicates, and verify the maintenance of target temperatures under simulation conditions.

Command line in GROMACS to generate .xvg files for the analysis:

```
gmx energy -f Simulation.edr -o Termperature_simulation.xvg
```

```
def temperature_analysis(output_folder, *simulation_file_groups, temp_config=None,␣
↪density_config=None)
```

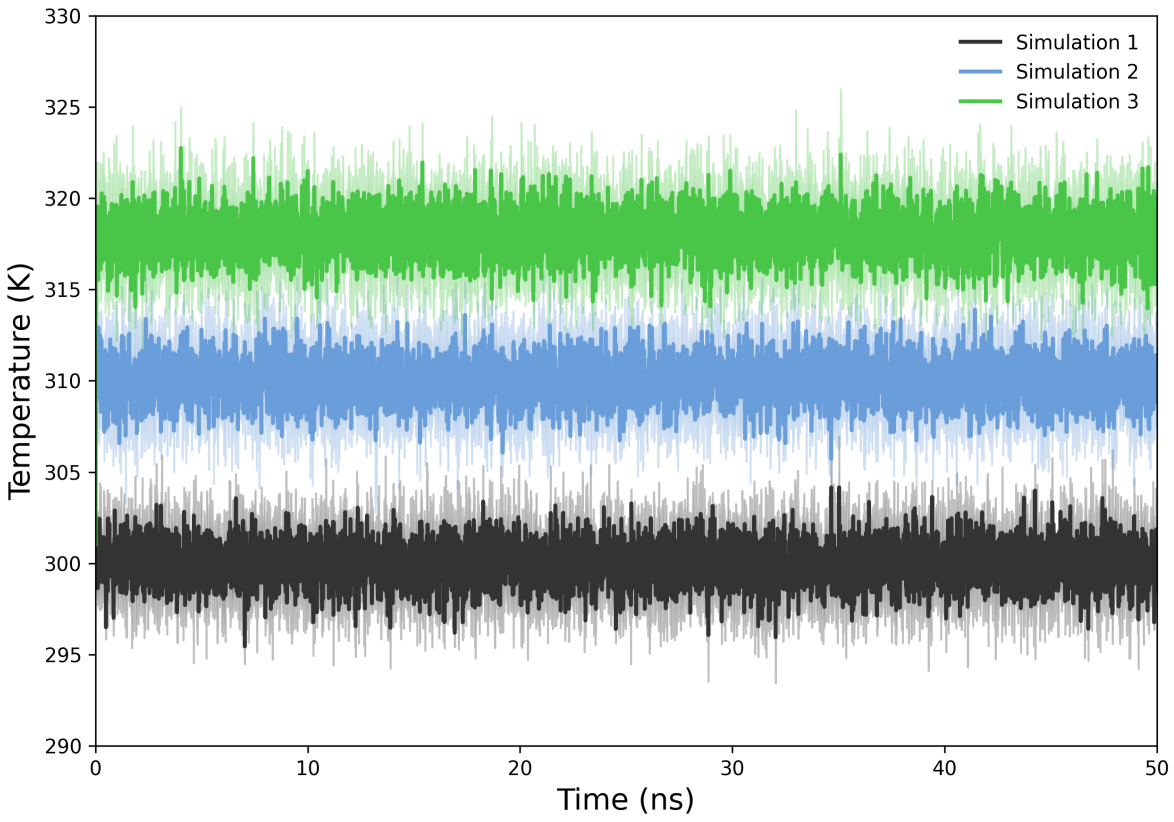

Interpretation guidance: The resulting plots show the temporal evolution of system temperature. A stable temperature profile indicates proper equilibration and control of the simulation ensemble. Significant fluctuations or trends may suggest insufficient thermal coupling, poor equilibration, or external perturbations affecting thermal stability.

```
def plot_temperature_density(results, output_folder, config=None)
```

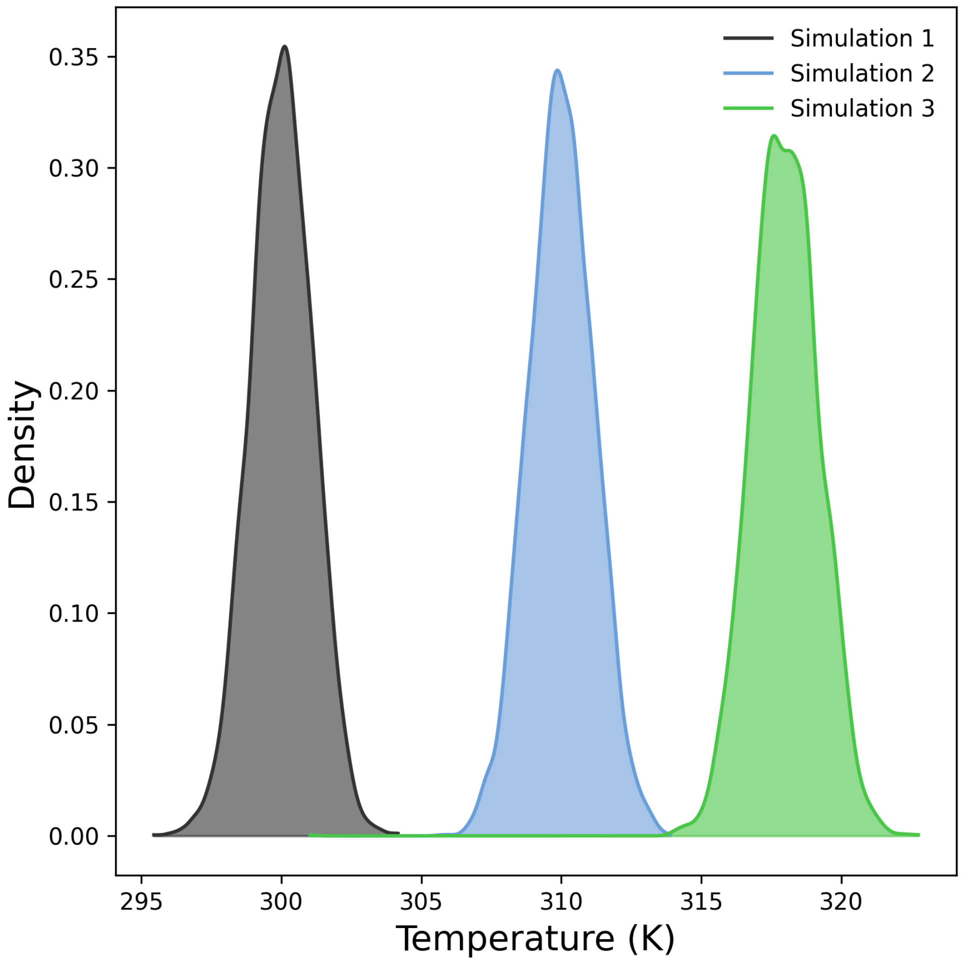

Interpretation guidance: This plot represents the distribution of temperature values throughout the simulation. Sharp, narrow peaks indicate stable and consistent thermal behavior, while broader peaks suggest greater variability or temperature fluctuations. Comparing distributions across simulations can help identify differences in thermal stability or ensemble control.

23.2 Complete code

```
import numpy as np
import matplotlib.pyplot as plt
from scipy.stats import gaussian_kde
import os
```

```
def read_temperature(file):
```

```
"""
Reads temperature data from a .xvg file and returns time (in ns) and temperature␣
↪values.
"""
try:
    times = []
    temps = []
    with open(file, 'r') as f:
        for line in f:
            # Skip comments and empty lines
```

(continues on next page)

(continued from previous page)

```
            if line.startswith(('#', '@', ';')) or line.strip() == '':
                continue
            try:
                values = line.split()
                if len(values) >= 2:
                    time_ps, temp_val = map(float, values[:2])
                    times.append(time_ps / 1000.0)  # Convert ps to ns
                    temps.append(temp_val)
            except ValueError:
                # Skip lines with parsing errors
                continue
    if len(times) == 0 or len(temps) == 0:
        raise ValueError(f"File {file} does not contain valid data.")
    return np.array(times), np.array(temps)
except Exception as e:
    print(f"Error reading file {file}: {e}")
    return None, None
```

```
def check_simulation_times(*time_arrays):
```

```
for i in range(1, len(time_arrays)):
    if not np.allclose(time_arrays[0], time_arrays[i]):
        raise ValueError(f"Simulation times do not match between file 1 and file
↪{i+1}")
```

```
def plot_temperature(results, output_folder, config=None):
```

```
plt.figure(figsize=config.get('figsize', (9, 6)))

# Loop over each simulation group's results
for idx, (time, mean, std) in enumerate(results):
    label = config['labels'][idx] if config and 'labels' in config else f
↪'Simulation {idx+1}'
    color = config['colors'][idx] if config and 'colors' in config else None
    alpha = config.get('alpha', 0.2) if config else 0.2

    # Plot mean temperature line
    plt.plot(time, mean, label=label, color=color, linewidth=2)
    # Fill area between mean ± std deviation
    plt.fill_between(time, mean - std, mean + std, color=color, alpha=alpha)

# Set axis labels and font sizes
plt.xlabel(config.get('xlabel', 'Time (ns)'), fontsize=config.get('label_fontsize',
↪ 12))
plt.ylabel(config.get('ylabel', 'Temperature (K)'), fontsize=config.get('label_
↪fontsize', 12))

plt.legend(frameon=False, loc='upper right', fontsize=10)
plt.tick_params(axis='both', which='major', labelsize=10)

# Set x-axis limits from 0 to max time
max_time = max([np.max(time) for time, _, _ in results])
plt.xlim(0, max_time)
# Set fixed y-axis range for temperature visualization
plt.ylim(290, 310)
```

(continues on next page)

(continued from previous page)

```
plt.tight_layout()
os.makedirs(output_folder, exist_ok=True)
plt.savefig(os.path.join(output_folder, 'temperature_plot.tiff'), dpi=300)
plt.savefig(os.path.join(output_folder, 'temperature_plot.png'), dpi=300)
plt.show()
```

```
def plot_temperature_density(results, output_folder, config=None):
```

```
plt.figure(figsize=config.get('figsize', (6, 6)))

# Loop over each simulation group's mean temperature for KDE
for idx, (_, mean, _) in enumerate(results):
    kde = gaussian_kde(mean)
    x_vals = np.linspace(290, 310, 1000)  # Define KDE evaluation range
    label = config['labels'][idx] if config and 'labels' in config else f
↪'Simulation {idx+1}'
    color = config['colors'][idx] if config and 'colors' in config else None
    alpha = config.get('alpha', 0.5) if config else 0.5

    # Plot filled KDE curve
    plt.fill_between(x_vals, kde(x_vals), color=color, alpha=alpha, label=label)

# Set axis labels and font sizes
plt.xlabel(config.get('xlabel', 'Temperature (K)'), fontsize=config.get('label_
↪fontsize', 12))
plt.ylabel(config.get('ylabel', 'Density'), fontsize=config.get('label_fontsize',␣
↪12))

plt.legend(frameon=False, loc='upper right', fontsize=10)
plt.tight_layout()
os.makedirs(output_folder, exist_ok=True)
plt.savefig(os.path.join(output_folder, 'temperature_density.tiff'), dpi=300)
plt.savefig(os.path.join(output_folder, 'temperature_density.png'), dpi=300)
plt.show()
```

```
def temperature_analysis(output_folder, *simulation_file_groups, temp_config=None,␣
↪density_config=None):
```

```
"""
Processes multiple groups of temperature replicates, calculates mean and std, and␣
↪plots results.

Parameters:
- output_folder: str, directory to save plots
- simulation_file_groups: lists of replicate file paths for each simulation group
- temp_config: dict (optional), plot config for time series
- density_config: dict (optional), plot config for KDE density
"""
results = []

# Process each simulation group (each group has multiple replicate files)
for group in simulation_file_groups:
    if group:
        times = []
```

(continues on next page)

(continued from previous page)

```
        temps = []
        for file in group:
            time, temp_val = read_temperature(file)
            if time is None or temp_val is None:
                raise ValueError(f"Error reading file: {file}")
            times.append(time)
            temps.append(temp_val)
        # Check that all replicates have matching time arrays
        check_simulation_times(*times)
        temps_array = np.array(temps)
        mean_temp = np.mean(temps_array, axis=0)
        std_temp = np.std(temps_array, axis=0)
        results.append((times[0], mean_temp, std_temp))

# Plot results if any simulation groups were processed
if len(results) >= 1:
    plot_temperature(results, output_folder, config=temp_config)
    plot_temperature_density(results, output_folder, config=density_config)
else:
    raise ValueError("At least one simulation group is required.")
```

24 System Density

24.1 Overview

DynamiSpectra offers a comprehensive analytical framework to evaluate the density distribution within molecular systems during molecular dynamics simulations, utilizing input data in .xvg file format. This analysis enables detailed insights into the spatial arrangement and concentration of particles in the system.

The software's graphical interface allows users to visualize the average density profiles derived from multiple simulation replicates, with the corresponding standard deviation depicted as a shaded region to represent variability. Additionally, *DynamiSpectra* supports the analysis of individual simulation replicas, enabling plots to be generated from single datasets when multiple replicates are not available or not required.

Command line in GROMACS to generate .xvg files for the analysis:

```
gmx energy -f Simulation.edr -o Density.xvg
```

```
def density_analysis(output_folder, *simulation_file_groups, density_config=None,␣
↪distribution_config=None)
```

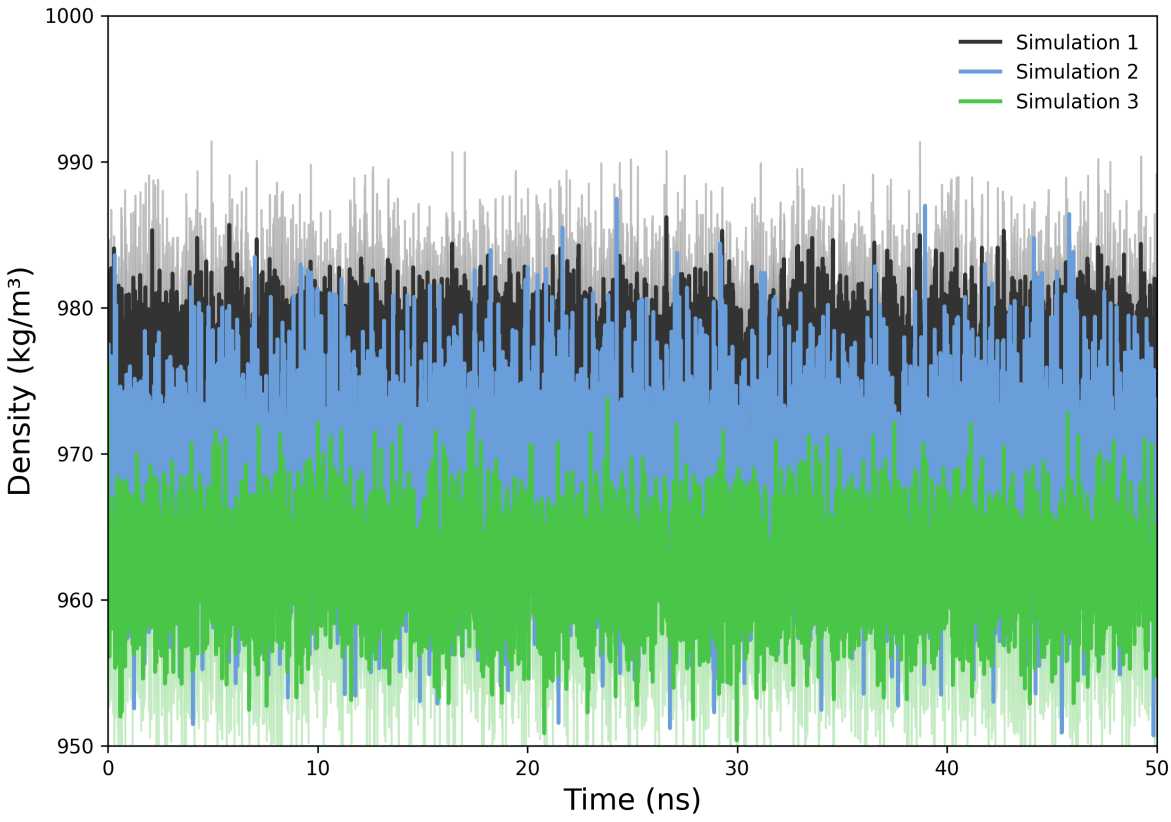

Interpretation guidance: The resulting plots illustrate the temporal evolution of system density. A consistent profile indicates stable particle distribution throughout the simulation, while significant fluctuations may reflect structural rearrangements, phase transitions, or other dynamic processes affecting system compactness or organization of the system.

```
def plot_density_distribution(results, output_folder, config=None)
```

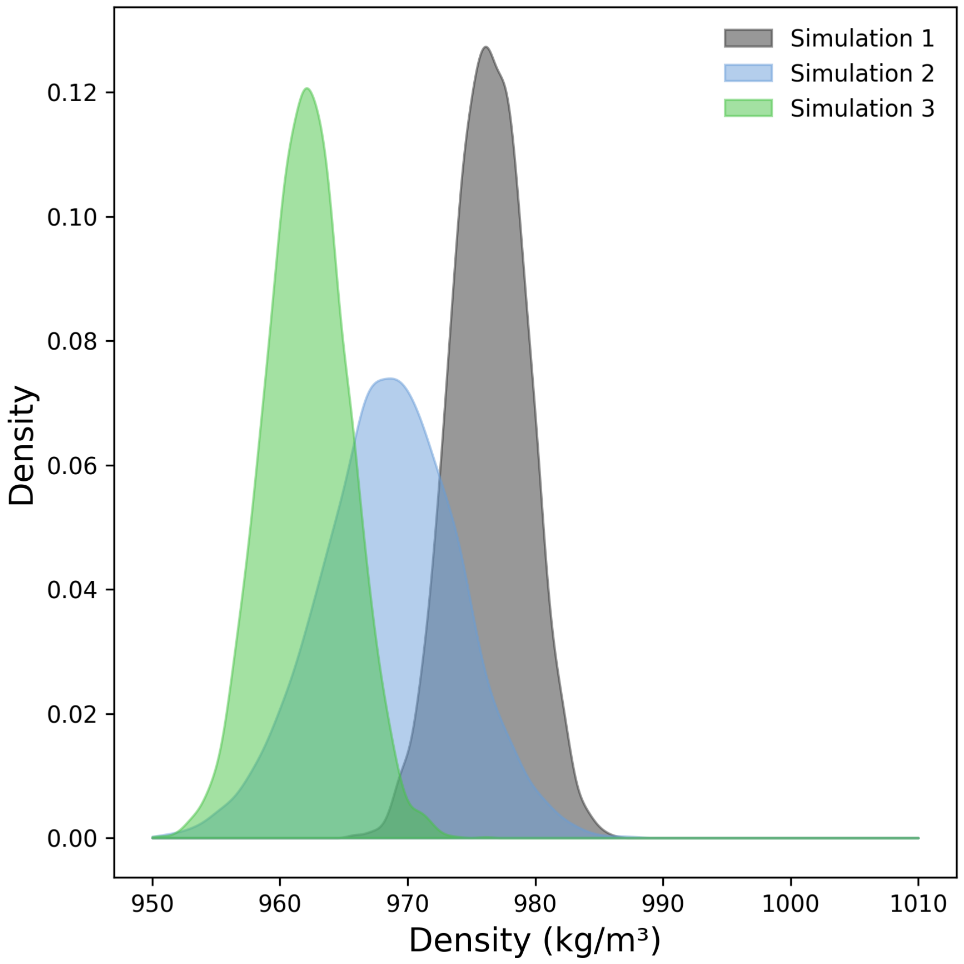

Interpretation guidance: This plot represents the density distribution derived from the temporal profile of system density. Peaks in the distribution indicate regions of higher particle concentration within the system. Broader peaks suggest a more heterogeneous or variable distribution, whereas sharper, well-defined peaks correspond to stable and consistent density regions throughout the simulation.

24.2 Complete code

```
import numpy as np
import matplotlib.pyplot as plt
from scipy.stats import gaussian_kde
import os
```

```
def read_density(file):
```

```
"""
Reads density data from a .xvg file and returns time (in ns) and density values.
"""
try:
    times = []
    densities = []
    with open(file, 'r') as f:
        for line in f:
            # Skip comments and formatting lines
            if line.startswith(('#', '@', ';')) or line.strip() == '':
```

(continues on next page)

(continued from previous page)

```
                continue
            try:
                values = line.split()
                if len(values) >= 2:
                    # Extract time (ps) and density, then convert time to ns
                    time_ps, density_val = map(float, values[:2])
                    times.append(time_ps / 1000.0)
                    densities.append(density_val)
            except ValueError:
                # Ignore lines that cannot be converted to floats
                continue
    # Raise error if no valid data found
    if len(times) == 0 or len(densities) == 0:
        raise ValueError(f"File {file} does not contain valid data.")
    return np.array(times), np.array(densities)
except Exception as e:
    print(f"Error reading file {file}: {e}")
    return None, None
```

```
def check_simulation_times(*time_arrays):
```

```
"""
Checks if all provided time arrays are consistent (equal within tolerance).
Raises an error if any discrepancy is found.
"""
for i in range(1, len(time_arrays)):
    if not np.allclose(time_arrays[0], time_arrays[i]):
        raise ValueError(f"Simulation times do not match between file 1 and file
↪{i+1}")
```

```
def plot_density(results, output_folder, config=None):
```

```
"""
Plots mean density over time with shaded standard deviation area for multiple␣
↪simulations.

Parameters:
-----------
results : list of tuples
    Each tuple contains (time_array, mean_density, std_density).
output_folder : str
    Path where plots will be saved.
config : dict, optional
    Dictionary with plot customization (colors, labels, figure size, etc.).
"""
plt.figure(figsize=config.get('figsize', (9, 6)))
for idx, (time, mean, std) in enumerate(results):
    # Set label and color for each simulation
    label = config['labels'][idx] if config and 'labels' in config else f
↪'Simulation {idx+1}'
    color = config['colors'][idx] if config and 'colors' in config else None
    alpha = config.get('alpha', 0.2)

    # Plot mean density line and shaded std deviation area
    plt.plot(time, mean, label=label, color=color, linewidth=2)
```

(continues on next page)

(continued from previous page)

```
    plt.fill_between(time, mean - std, mean + std, color=color, alpha=alpha)

# Set plot labels and legend
plt.xlabel(config.get('xlabel', 'Time (ns)'), fontsize=config.get('label_fontsize',
↪ 12))
plt.ylabel(config.get('ylabel', 'Density (kg/m³)'), fontsize=config.get('label_
↪fontsize', 12))
plt.legend(frameon=False, loc='upper right', fontsize=10)
plt.tick_params(axis='both', which='major', labelsize=10)

# Set axis limits dynamically if specified
max_time = max([np.max(time) for time, _, _ in results])
plt.xlim(0, max_time)
if 'ylim' in config:
    plt.ylim(config['ylim'])

# Save plots to output folder
plt.tight_layout()
os.makedirs(output_folder, exist_ok=True)
plt.savefig(os.path.join(output_folder, 'density_plot.tiff'), dpi=300)
plt.savefig(os.path.join(output_folder, 'density_plot.png'), dpi=300)
plt.show()
```

```
def plot_density_distribution(results, output_folder, config=None):
```

```
"""
Plots KDE-based smooth distributions of mean density values for multiple␣
↪simulations.

Parameters:
-----------
results : list of tuples
    Each tuple contains (time_array, mean_density, std_density).
    Only mean_density is used for KDE estimation.
output_folder : str
    Path where plots will be saved.
config : dict, optional
    Dictionary with plot customization (colors, labels, figure size, etc.).
"""
plt.figure(figsize=config.get('figsize', (6, 6)))
for idx, (_, mean, _) in enumerate(results):
    # Kernel Density Estimation for smooth density distribution
    kde = gaussian_kde(mean)
    x_min = config.get('x_min', np.min(mean))
    x_max = config.get('x_max', np.max(mean))
    x_vals = np.linspace(x_min, x_max, 1000)

    # Plot KDE fill for each simulation
    label = config['labels'][idx] if config and 'labels' in config else f
↪'Simulation {idx+1}'
    color = config['colors'][idx] if config and 'colors' in config else None
    alpha = config.get('alpha', 0.5)
    plt.fill_between(x_vals, kde(x_vals), color=color, alpha=alpha, label=label)

# Set plot labels and legend
plt.xlabel(config.get('xlabel', 'Density (kg/m³)'), fontsize=config.get('label_
```

(continues on next page)

(continued from previous page)

```
↪fontsize', 12))
plt.ylabel(config.get('ylabel', 'Density'), fontsize=config.get('label_fontsize',␣
↪12))
plt.legend(frameon=False, loc='upper right', fontsize=10)
plt.tight_layout()

# Save KDE plots to output folder
plt.savefig(os.path.join(output_folder, 'density_distribution.tiff'), dpi=300)
plt.savefig(os.path.join(output_folder, 'density_distribution.png'), dpi=300)
plt.show()
```

```
def density_analysis(output_folder, *simulation_file_groups, density_config=None,␣
↪distribution_config=None):
```

```
"""
Processes multiple simulation groups with density replicate files,
computes mean and standard deviation, and generates plots.

Parameters:
-----------
output_folder : str
    Directory path where plots will be saved.
*simulation_file_groups : list of lists
    Each argument is a list of file paths for replicate density data of one␣
↪simulation.
density_config : dict, optional
    Configuration dictionary for time series density plot.
distribution_config : dict, optional
    Configuration dictionary for density distribution (KDE) plot.
"""
def process_group(file_paths):
    times = []
    densities = []
    for file in file_paths:
        # Read density data from each replicate file
        time, density_val = read_density(file)
        times.append(time)
        densities.append(density_val)

    # Check that all replicates have consistent time points
    check_simulation_times(*times)

    # Calculate mean and standard deviation across replicates
    density_array = np.array(densities)
    mean_density = np.mean(density_array, axis=0)
    std_density = np.std(density_array, axis=0)
    return times[0], mean_density, std_density

results = []
for group in simulation_file_groups:
    if group:
        time, mean, std = process_group(group)
        results.append((time, mean, std))

# Generate plots if at least one simulation group is processed
if len(results) >= 1:
```

(continues on next page)

(continued from previous page)

```
    plot_density(results, output_folder, config=density_config)
    plot_density_distribution(results, output_folder, config=distribution_config)
else:
    raise ValueError("At least one simulation group with replicate files is␣
↪required.")
```

25 Contributing

Contributions are welcome, and they are greatly appreciated! Every little bit helps, and credit will always be given.

25.1 Bug reports

When reporting a bug please include:

- Your operating system name and version.
- Any details about your local setup that might be helpful in troubleshooting.
- Detailed steps to reproduce the bug.

25.2 Documentation improvements

DynamiSpectra could always use more documentation, whether as part of the official DynamiSpectra docs, in docstrings, or even on the web in blog posts, articles, and such.

25.3 Feature requests and feedback

The best way to send feedback is to file an issue at https://github.com/Conradoou/DynamiSpectra/issues.

If you are proposing a feature:

- Explain in detail how it would work.
- Keep the scope as narrow as possible, to make it easier to implement.
- Remember that this is a volunteer-driven project, and that code contributions are welcome.

26

Authors

- Iverson Conrado Bezerra - https://github.com/Conradoou
- Orcid - https://orcid.org/0000-0003-0524-8913

27

Changelog

27.1 1.1.0 (2025-07-08)

- Added analysis of protein-ligand interactions: - Contact maps - Hydrophobic contacts - Salt bridges
- Added new system-level analyses: - System density - Temperature - Pressure
- Added advanced structure-based analyses: - Ligand dihedral angles - Protein side chain rotamers (Chi1 vs Chi2) - Phi and Psi backbone angles
- Added distance matrix analysis.

27.2 1.0.6 (2025-05-01)

- Initial release with core trajectory analysis features: - RMSD (Root Mean Square Deviation) - RMSF (Root Mean Square Fluctuation) - Radius of Gyration (Rg) - Hydrogen bonds (Hbond) - Secondary structure analysis - Ligand density (3D and projected)

28 Indices and tables

- genindex
- modindex
- search
